# On the robustness of gender differences in economic behavior

**DOI:** 10.1038/s41598-022-25141-1

**Published:** 2022-12-15

**Authors:** Helena Fornwagner, Brit Grosskopf, Alexander Lauf, Vanessa Schöller, Silvio Städter

**Affiliations:** 1 Department of Economics, University of Exeter, Exeter, EX4 4PU United Kingdom; 2 Department of Economics, University of Regensburg, 93053 Regensburg, Germany

**Keywords:** Human behaviour, Psychology and behaviour

## Abstract

Because of the importance of economic decisions, researchers have looked into what factors influence them. Gender has received a lot of attention for explaining differences in behavior. But how much can be associated with gender, and how much with an individual’s biological sex? We run an experimental online study with cis- and transgender participants that (1) looks into correlational differences between gender and sex for competitiveness, risk-taking, and altruism by comparing decisions across these different subject groups. (2) we prime participants with either a masculine or feminine gender identity to examine causal gender effects on behavior. We hypothesize that if gender is indeed a primary factor for decision-making, (i) individuals of the same gender (but different sex) make similar decisions, and (ii) gender priming changes behavior. Based on 780 observations, we conclude that the role of gender (and sex) is not as decisive for economic behavior as originally thought.

## Introduction

Worldwide, humans make economic decisions every day: Should I apply for a new job opportunity in a highly competitive environment? Should I invest in a risky asset or not? How much money should I donate to charities? A vast literature tries to determine the factors that affect decisions in domains such as competitiveness^1^, risk-taking^2^, and altruism^3^. Researchers have looked, among other things, into the role of institutional or market-related features^4–11^, cultural background^12–17^, individual characteristics^18–23^, hormonal^24–29^, or other biological factors, such as genetics, and neurological factors^30–34^. Among those factors, gender has received a lot of attention. Over the last few decades, the flourishing research in economics has looked at whether gender is a significant driver of how women and men behave in the domains of competitiveness^35^, risk-taking^2,36^, and altruism^3^. We refer to the Supplementary Sect. 14 for a detailed literature review.

But is it really gender that influences behavior? Or, instead, are sex differences causing these observed differences? Or is it a mix of gender and sex? Importantly, sex and gender are two distinct concepts. Whereas sex is defined as “either of the two main categories (male and female) into which humans” are categorized based on their reproductive functions (www.oed.com, accessed 2021-10-12), gender usually refers to the psychological, behavioral, social, and cultural aspects of being male or female (i.e., masculinity or femininity)^37–39^. For cisgender individuals, the internal gender identity matches and presents itself by the externally determined cultural expectations of the behavior and roles considered appropriate for one’s sex^37^. However, the gender identity of transmen and transwomen and their gender roles are typically not the same as what is associated with their sex assigned at birth^40^. So the question arises: how much of the differences of men and women often found in the economic literature can really be associated with gender as opposed to an individual’s sex?

We investigate this question by using well-known behavioral economic experiments in the domain of *competitiveness*, *risky choices*, and *altruism*. As stated, for these three behavioral traits, gender differences are a common finding. However, existing studies identify gender effects, without controlling for sex. Distinguishing gender from sex effects is practically impossible when only investigating cisgender participants. As a novel approach, we run our experimental study with transmen and transwomen in addition to cismen and ciswomen. We do not use the gender that is attributed to a person by others^38,39,41,42^. Instead, this study utilizes the information on the participants’ self-identification to a particular gender and sex from self-reported categories and established scaling methods from psychological and medical science. The advantage of having this information is that that cisgender and transgender people differ in either their sex or their gender. To illustrate this consider an example: a ciswoman has female sex *and* feminine gender. A transman has female sex *but* masculine gender. So differences in the behavior of those two subject groups might be associated with gender instead of sex.

The experimental method is excellent for studying the economic choices we are interested in because of its standardized and validated measures. We have information on the participants’ gender and sex from self-reported categories and established scaling methods. Moreover, instead of just analyzing gender and sex effects correlationally, we elicit the causal impact of gender by exogenously varying gender identities with a priming method.

First, we test how gender correlates with the mentioned choices. By contrasting the behavior of the four different subject groups of cismen, ciswomen, transmen, and transwomen, we obtain insights into how far biology (sex) or the cultural and sociological construct of gender explains differences in economic behavior. Our study is the first investigating competitiveness, risk-taking, and altruism of transmen and transwomen. We hypothesize that if gender is the driving factor, individuals of the same gender (and different sex) make similar decisions, and decisions significantly differ when gender differs (and sex is the same). Second, we concentrate on the causal effect of gender on behavior—an analysis that is rarely done in the literature. The traditional experimental method of randomizing over the variable of interest is not possible with gender. Hence, we need a different approach to elicit causal effects. As our method to test a directional impact of gender, we employ a gender prime: either a masculine or feminine gender identity is subconsciously activated. Priming is an easy-to-implement intervention that has shown to influence individual decision-making in various dimensions. Amongst others, it has been used to activate gender identities or change gender stereotypes^43,44^. Those studies’ results are mixed, depending on the objective of the prime (e.g., risk preferences, competitiveness, altruism) and the method of priming (eliciting gender at the beginning of the study or showing pictures).

In our study, we use a word priming method that has shown to be powerful in other contexts^45–47^, and has the advantage that we can easily include a gender-neutral condition by using gender-neutral words. In general, it seems to be the case that different genders react differently to gender priming. Importantly, none of the existing priming studies has recruited transgender subjects as researchers usually rely only on self-reported (binary) gender identities. If cisgender and transgender individuals change their behavior when being primed, this would indicate a causal effect of gender on individual economic decisions. To be more specific, our hypotheses are as follows. First, since our priming affects individuals’ gender identity and not their sex, we anticipate participants with the same gender to react similarly to the respective prime. Put differently, cismen and transmen (ciswomen and transwomen) should adjust their behavior similarly when being primed. Second, we expect reactions to priming to be different when the gender is not the same among the participants. Lastly, the results should be different when participants are primed with their own gender identity instead of their respective other gender identity.

Based on 780 observations from experiments conducted online, our results generally show no correlational or causal effect of gender or sex for competitiveness, risk-taking, and altruism. The only exceptions are that cismen have a higher rate of entering the competition than all other subject groups when primed masculine. They also risk more when primed with a masculine identity compared to the neutral priming condition. In addition, we find that subjects of male sex (i.e., cismen and transwomen) risk more than their female counterparts (ciswomen and transmen). However, these behavioral differences that sometimes point towards gender and sometimes towards sex as explanatory variables do not replicate if we apply different robustness tests, including correcting for multiple hypothesis testing. Thus, we conclude that neither gender nor sex is a consistent main factor influencing the economic decisions measured in this article.

## Methods

To test our research questions, we set up an online economic experiment. This experiment received ethical approval from the UEBS Research Ethics Committee of the University of Exeter (Ethics application—eUEBS004241; 26.05.2021) and the Ethics Committee of the University of Regensburg (28.04.2021). All research was performed in accordance with the relevant guidelines and regulations. We have obtained informed consent from all participants. The study was preregistered on aspredicted.org (Nr. 68888) before data collection (see https://aspredicted.org/rc9vn.pdf). We conduct our study (tasks and questionnaires) with oTree^48^ on Prolific (www.prolific.co). To recruit the different subject groups, we used specific filters provided by Prolific. Prolific was especially well suited to host our study as they have a pool of subjects who registered as being either a transman or a transwoman. We used the Prolific filters on gender identity to recruit our subjects. However, our classification into the subject groups cismen, ciswomen, transmen, and transwomen is based on the self-reported information we elicited with the experimental questionnaire.

Each participant completes six parts and several questionnaires. One part is randomly selected for payment at the end of the experiment. In Part 1, a participant is randomly assigned to either the baseline treatment (NEUTRAL) or a treatment condition that refers to one of the gender priming interventions: FEMININE (primes a feminine gender identity) or MASCULINE (primes a masculine gender identity). Participants are primed by a word search task where different words are used depending on the underlying treatment^46^. The words in FEMININE are: female, woman, she, women, her, girl, hers, lady; in MASCULINE they are: male, man, he, men, him, boy, his, gentleman. In the baseline condition NEUTRAL, participants also solve the word search task, with the following (neutral) words: person, it, people, its, child, theirs, individual, neuter. Participants are shown the words and have two minutes to mark these words in a 10 × 10 grid. In case they find all words, they receive $\pounds$5.

After the word search task, each participant enters the next parts of the experiments, which are the respective economic decision–making parts. As our first decision dimension, we employ monetary incentives to measure competitiveness^49^. We measure the performance in a real effort math task, where the participants are instructed to solve puzzles by finding two two-digit numbers that add up to 100 in 3 × 3 matrices for two minutes. In Part 2, they complete the math task under piece-rate incentives, which means they receive $\pounds$0.50 for every solved puzzle. In Part 3, the same math task is performed under tournament incentives. The participants are divided into groups of four and receive $\pounds$2 for every solved puzzle, but only if they solve more puzzles than every other group member. In Part 4, the participants have to choose, before performing, whether their performance in this part will be paid based on the piece-rate incentives (like Part 2) or according to the tournament rules (like Part 3). Whenever a participant decides on the tournament incentives in Part 4, s/he is classified as competitive and competes against the group member’s performance in the previous Part 3. In all parts, the participants do not receive feedback on how well they perform compared to the other group members until the end of the experiment and have no information on the other group members’ identity or characteristics. Additionally, we measure the participants’ confidence in Part 2 (how well they think they performed compared to the other participants in the session) and Part 3 (how well they think they performed compared to the other group members) with incentivized questions.

Our second decision dimension is the willingness to take risks (Part 5). It is measured using a simple lottery task^50^. Participants receive $\pounds$4 and can invest into a lottery with a 50% chance of success. The invested amount is multiplied by 2.5 in case of success. In case of no success, the invested amount is lost. The participants keep the amount not invested. Risk preferences are measured as the amount a participant invests, where higher investments indicate a higher willingness to take risks. The third decision dimension is altruism (Part 6). We investigate the participants’ altruistic preferences with a dictator game^51^. Participants receive $\pounds$5 and split up this amount between themselves and up to five different charities. Altruism is quantified as the sum donated by a participant.

The post-experimental questionnaire contains (1) a 30-items version of the Bem Sex Role Inventory (BEM) that explores a person’s masculine and feminine self-identification on a continuous scale^52^; (2) the Transgender Congruence Scale (TCS)^53^ which evaluates if and how much someone identifies as transgender; (3) demographic questions, as well as questions on the biological sex, gender, sexual orientation, and whether one self-identifies as transgender; and (4) the Steps to Transition (STT) questionnaire that describes typical steps transgender people undertake in their transition^53^. This questionnaire controls for aspects like legally changing a name, undergoing hormone replacement therapy, having surgery to alter genitalia, or a non-genital surgery like a breast removal. In addition, we include debriefing questions to check if the participants are aware of the study topic and the priming intervention^54^.

The last section of the Supplementary Information provides a detailed description of all instructions and questionnaires (including the screenshots) of our experiment.

## Results

Presenting our results, we use the following abbreviations: Brown-Forsythe test (BF), Chi-squared test ($\chi ^2$), Kruskal-Wallis test (KW), two-tailed Kendall’s rank correlation coefficients test (KTAU), two-tailed Mann-Whitney U test (MWU), Robust Wald test (W), two-tailed Variance Ratio test (VR), Cohen’s *d* (*d*), and standard deviation (SD). The significance levels are defined as follows: $p~<~0.05$ (*), $p~<~0.01$ (**), and $p~<~0.001$ (***), where a significant result must have at least $p~<~0.05$. We summarize multiple *p*-values by $p's$.

### Descriptives

We collected a total of $n~=~780$ observations, out of which 425 are cisgender (214 cismen and 211 ciswomen) and 355 transgender (215 transmen and 140 transwomen; see the Supplementary Subsect. 15.1 for more details). The questionnaire is used to classify one subject into one of the four groups, which asks about their current gender, sex, and whether they self-identify as transgender. We generally find support for the classification into groups according to the guidelines of the American Psychological Association^40^, as the data indicate that for only 5.07% of the transgender individuals their sex changed since birth.

We did a pre-experimental power analysis to calculate the needed sample sizes based on existing work^4^. We used their neutral priming condition to inform our power calculations. Based on their effect size delta of $-\,0.264$, the needed observations for $\alpha ~=~0.05$ and a power of 0.80 are 44 for one subject group in one treatment. Following, it would be enough to have in total $n~=~528$. To be more conservative, we preregistered having 72 observations for each subject group in each treatment, resulting in a power of 0.95 (see https://aspredicted.org/rc9vn.pdf for further information). In our particular case of having a non-usual subject group of transgender individuals, we already mentioned in the preregistration that having 72 is very ambitious for transgender individuals, also because of the number of registered transgender individuals on Prolific. We ended up in NEUTRAL with the minimum needed amount of 44 transwomen. Consequently, we had a priori, based on the ex-ante preregistered power calculations and depending on the underlying comparison, at least a power of 0.82. The power increases up to 0.95 for the subject groups with $n=72$ in one treatment.

As summarized in Supplementary Table 1, the participants are on average 24.4 years old (SD = 6.60), have an average height in centimeters of 170 (SD = 10.8), and approximately half of them are students (47.2%). Around one third holds a university degree, 69.4% have an income lower than £20,000, and 25.8% report being religious. Our sample consists mostly of participants from the United States, followed by Continental Europe and the United Kingdom. Less than 10% live outside these three mentioned regions. Responses to the BEM classify 28.5% as feminine, 19.4% as masculine, 24.1% as androgynous, and 28.1% as undifferentiated. On the TCS scale ranging from 1 to 5, participants show an average score of 3.67 (SD = 1.1). The average score on the STT, which ranges from 0 to 16, is 4.35 (SD = 4.6). The various subject groups are comparable in several characteristics as indicated by the statistical tests added in Supplementary Table 1. Descriptive statistics broken down by subject groups are presented in Supplementary Tables 2 and 3 (cisgender) as well as Supplementary Tables 4 and 5 (transgender).

For the outcomes of Part 1, the Supplementary Sect. 2 includes the detailed summarizing descriptives on the participants’ priming. On average, the participants marked 7.45 out of 8 words (SD = 1.53), and 83.97% (i.e., $n~=~655$) marked all words from the list within the given time of two minutes.

### Competitiveness

**Figure 1:**
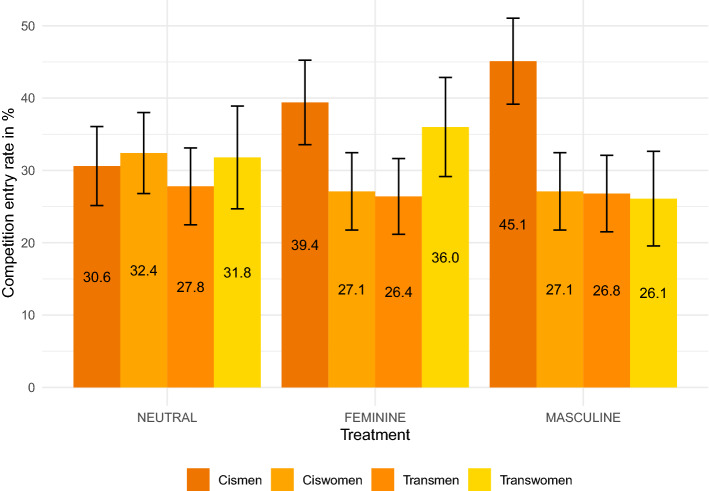
Tournament entry rates in Part 4 by treatment and subject groups in alphabetical order ($n~=~780$). The bars show the percentage of participants (between 0 and 100) who chose to compete rather than to perform under piece–rate incentives. The error bars represent the standard errors of the means.

Figure 1 and Supplementary Table 14 summarize the tournament entry rates in Part 4. In order to investigate whether gender and competitiveness are correlated, we focus on the baseline treatment NEUTRAL. No significant variation is reported across the four subject groups ($\chi ^2(3)~=~0.408$, $p~=~0.939$). Similar, when pooling the results by gender (Supplementary Fig. 2; cismen + transmen vs. ciswomen + transwomen), tournament entry rates do not differ for feminine and masculine subjects ($\chi ^2(1)~=~0.273$, $p~=~0.601$) and also no difference is found for male and female subjects when pooling the data by sex (Supplementary Fig. 3; cismen + transwomen vs. ciswomen + transmen; $\chi ^2(1)~=~0.028$, $p~=~0.867$). We compare the differences between the priming conditions (FEMININE and MASCULINE) and the baseline treatment (NEUTRAL) for the causal analysis. Priming does not influence the competition entry rates for any subject group ($\chi ^2(1)$, $p's~>~0.265$), including for cismen when comparing the MASCULINE treatment to the NEUTRAL treatment ($\chi ^2(1)$, $p~=~0.073$). We shall see in the regression analysis that when adding further controls, the impact of MASCULINE priming on cismen becomes significant. Looking at the MASCULINE priming condition only, where the entry rates look very similar for all subject groups except for cismen, the competition entry rate is around 20 percentage points higher for cismen than for all other subject groups ($\chi ^2(3)~=~7.991$, $p~=~0.046$).

In Supplementary Table 15, we run Probit regressions for the baseline treatment (NEUTRAL) to disentangle the effects of gender and sex. As our basic regression framework, we have in column (1) just the subject groups and in (2) additionally controls for the performance measures in the real effort task. In column (3), we further take into account the participant’s confidence and willingness to take risks. In column (4), we add the variables age, height, student status, income, religion, and residence, whereas in (5), we control for the outcomes in the TCS and STT. The TCS is interesting in our setting as it accounts for how much individuals feel genuine, authentic, and comfortable with their gender identity and external appearance. Similarly important, the STT measures details about the transition process, especially biological aspects like whether one has had surgery to alter genitalia, a non-genital surgery (like breast removal), or is undergoing hormone replacement therapy. Using joint coefficient tests (see Supplementary Table 15), we find neither gender (W, $\chi ^2(1)$, $p's~>~0.437$) nor sex (W, $\chi ^2(1)$, $p's~>~0.214$) to have a significant effect on competitiveness. We thus conclude that there is no correlation between neither gender nor sex and competitiveness in our study.

To analyze a potential causal effect of gender, we run Probit regressions in Supplementary Table 16. The non–parametrized analyses are confirmed for ciswomen, transmen, and transwomen. For cismen we find that the gender prime with MASCULINE has a significant impact increasing the competition entry rates in specification (2) (coef = 0.473; 95% CI = 0.036, 0.909; $p~=~0.034$; controlling for performance) and (4) (coef = 0.544; 95% CI = 0.076, 1.012; $p~=~0.021$; controlling for beliefs, risk attitude, and other person-specific covariates). Summing up, only cismen’s competition entry rates seem to be influenced (positively) when priming them with their own gender identity. We do not find a significant impact of gender priming for all other subject groups and priming combinations. We will interpret those results in the Discussion.

Our experimental design does not only allow us to look into the choice to enter a tournament but also into participants’ confidence (i.e., how well they believe they performed in the real effort task when competing, see Supplementary Table 11). In NEUTRAL, there is no evidence that subjects of masculine gender have higher performance beliefs than subjects of feminine gender (MWU, $z~=~-0.912$, $p~=~0.362$). However, we do find differences between subjects of female and male sex (MWU, $z~=~-3.470$, $p~=~0.001$). For priming, no subject group increases or decreases their beliefs when being primed (MWU, $p's~>~0.177$). Regressions in Supplementary Table 12 confirm that beliefs depend on the participants’ sex: male subjects generally have higher confidence in their performance than female subjects (W, *F*(1), $p's~<~0.001$). And again, confidence does not differ across gender (W, *F*(1), $p's~>~0.259$). That gender does not play a role in this setting is further confirmed when looking at the causal impact of gender priming on the participants’ confidence. For none of the subject groups, we do find any effect of gender priming on the beliefs when using regression analyses (see Supplementary Table 13, W, *F*(1), $p's~>~0.178$).

Another interesting aspect is to see in how far behavior pays off in the competitiveness task. We provide details and various analyses of the performances in the real effort task and the related payoffs of Part 2 to 4 in Supplementary Sect. 3.

### Risk

**Figure 2:**
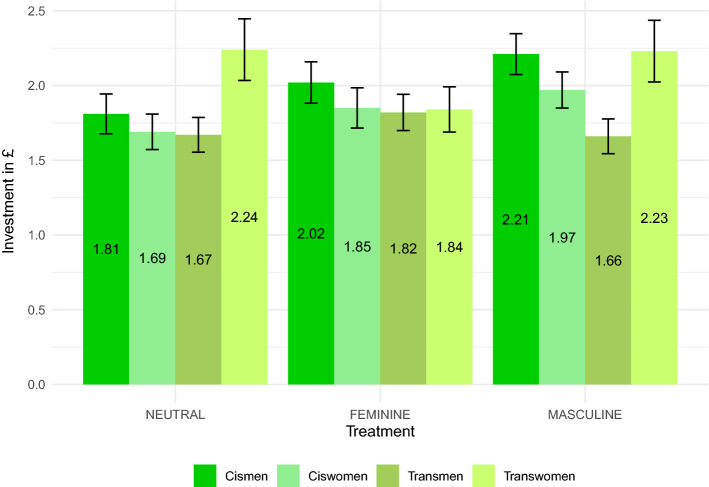
Investments into the risky lottery in Part 5 by treatment and subject groups in alphabetical order ($n~=~780$). The bars show the average investment rate, and the error bars represent the standard errors of the means.

Investment rates in the lottery are depicted in Fig. 2 and stated in Supplementary Table 20. When applying non–parametric tests, we do not find any differences between the various subject groups within the baseline treatment NEUTRAL (KW, chi-squared with ties =4.712 with 3 d.f., $p~=~0.194$). If anything, transwomen seem to be more risk-taking than transmen in a pairwise comparison (MWU, $z~=~-1.979$, $p~=~0.048$). This, however, does not point towards a systematic impact of gender and/or sex when pooling data (Supplementary Figs. 4 and 5; gender: cismen + transmen vs. ciswomen + transwomen, sex: cismen + transwomen vs. ciswomen + transmen; MWU, $p's~>~0.130$). Turning to the causal impact of priming, again, we see MASCULINE priming increases the risk attitude for cismen only (MWU, $z~=~2.075$ $p~=~0.038$) bringing the level of cismen to the one of transwomen in the MASCULINE priming (MWU, $z~=~0.156$, $p~=~0.876$). For every other subject group, we do not find any significant impact of gender priming (MWU, $p~>~0.206$).

Joint coefficient tests for the regressions (with and without control variables) in Supplementary Table 21 show the correlational results for our baseline condition. We find no differences in risk-taking of subjects of feminine and masculine gender (W, *F*(1), $p's~>~0.132$). However, we find a sex effect: male subjects risk more than female subjects (W, *F*(1), $p's~<~0.042$).

Turning to priming, we have significant differences in risk-taking of cismen when being primed MASCULINE (W, *F*(1), $p's~<~0.046$; see Supplementary Table 22). We find no difference in risk-taking for all other subject groups when primed with a gender (W, *F*(1), $p's~>~0.092$). The findings are independent of what other control variables are taken into account. The regression analysis for risk attitudes is thus similar to what we found for competition entry rates. When being primed with their own gender, only cismen significantly increase their risk-taking behavior.

### Altruism

**Figure 3:**
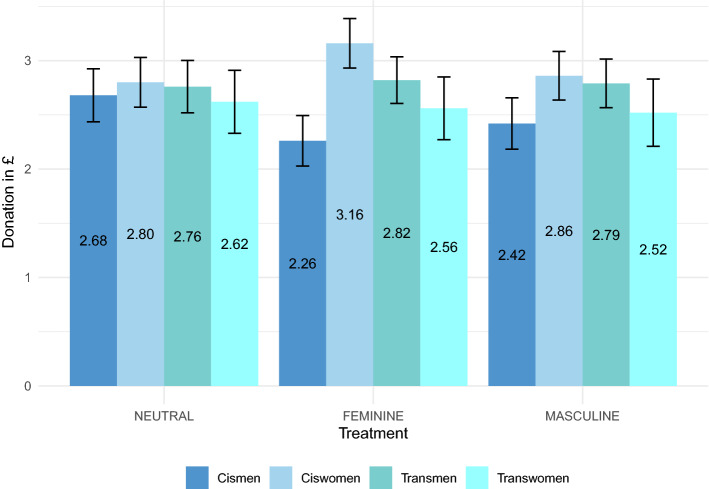
Donation in Part 6 by treatment and subject groups in alphabetical order ($n~=~780$). The average donations are indicated by the bars, and the error bars represent the standard errors of the means.

Last, we test for differences in the donation task (see Fig. 3 and Supplementary Table 24). Donations in NEUTRAL are not distinguishable across subject groups (KW, chi-squared with ties = 0.434 with 3 d.f., $p~=~0.933$). Neither pooled results for gender nor for sex yield a difference in donation rates (Supplementary Figs. 6 and 7; MWU, $p's~>~0.564$). Concerning the causal impact of gender priming, we do not find significant effects for any subject group and any priming condition (MWU, $p's~>~0.260$).

The regression analyses in Supplementary Tables 25 and 26 confirm these findings. Joint coefficient tests for gender or sex do not show significant correlations in the baseline condition (W *F*(1), $p's~>~0.580$). Moreover, the impact of all priming condition on all subject group remains insignificant, even after controlling for different sets of additional personal covariates (W, *F*(1), $p's~>~0.214$).

To summarize, we find no correlation between gender or sex on altruism and do not detect any causal impact of gender priming on altruistic behavior in our setup.

### Gender and sex differences within priming conditions

As we have shown so far, there is no systematic correlation between gender and behavior in the NEUTRAL treatment. Here we briefly test for gender and sex differences in behavior within the two other priming treatments. Looking at Supplementary Figs. 2 to 7 and analyzing the gender differences with non-parametric tests, we see no difference in competition entry rates across subject groups (FEMININE: $\chi ^2(1)~=~0.124$, $p~=~0.725$, MASCULINE: $\chi ^2(1)~=~2.488$, $p~=~0.115$), risk–taking (FEMININE: MWU, $z~=~0.584$ $p~=~0.560$, MASCULINE: MWU, $z~=~-0.663$, $p~=~0.507$), and altruism (FEMININE: MWU, $z~=~-1.507$, $p~=~0.132$, MASCULINE: MWU, $z~=~-0.625$, $p~=~0.532$). Turning to sex differences, the picture slightly changes. First, we see differences between subjects of male and female sex in both priming conditions (FEMININE and MASCULINE) for competitiveness. The differences are close to conventional levels of significance (FEMININE: $\chi ^2(1)~=~3.808$, $p~=~0.051$, MASCULINE: $\chi ^2(1)~=~3.349$, $p~=~0.067$). Second, for risk-taking, we find a significant difference in the MASCULINE treatment only, with subjects of male sex taking more risk than subjects of female sex (MWU, $z~=~2.558$ $p~=~0.011$). Third, for altruism, we find subjects of female sex having significantly higher scores than those of male sex in the FEMININE treatment (MWU, $z~=~-2.269$, $p~=~0.023$). Hence, for risk and altruism we find that only those sexes show higher scores who are primed with the gender identity that they would cisgender-stereotypically be associated with.

## Robustness tests

In the remainder of the article, we apply different approaches to test the robustness of our results for comparing behavior across subject groups within NEUTRAL and by subject groups across primings.

### Comparing variances instead of means

Recent literature argues that gender differences, for example, in preferences, often remain undetected because the researchers almost exclusively focus on differences in means^2,55^. It is suggested that when comparing variance ratios (i.e., the standard effect size measure for variance differences), one reliably finds evidence for greater male variability in cooperation, time, risk, social preferences, and academic grades. Thus, we rerun our analysis based on variance ratios for risk and altruism only, given that competitiveness is measured on a binary scale.

No significant differences in standard deviations of all subject groups within the baseline treatment NEUTRAL (BF(3,255), $W50~=~2.564$, $p~=~0.055$) are found for the lottery investment rates. Pooling the results for gender does again show no differences in the variances (VR(143, 114), $f~=~0.805$, $p~=~0.219$). Only the investment rate of male subjects has a greater variability compared to females when pooling data based on sex (VR(115, 142), $f~=~1.5617$, $p~=~0.012$). This result is in line with a recent meta-analysis, finding a significant difference in variances between men and women of 1.25^2^. Additionally, no causal impact of gender priming between any priming condition for any subject group (VR, $p's~>~0.100$) is reported.

The variances of the donations in NEUTRAL are not distinguishable across subject groups (BF(3, 255), $W50~=~1.100$, $p~=~0.350$). The literature reports a variance ratio between men and women of 1.18^2^, which is in line with the variance ratio in our sample of 1.144 between cismen and ciswomen. Neither pooled results for gender nor sex show significant differences in the variances of donation rates (VR, $p's~>~0.480$). Similarly, the donation rates do not differ based on the variances for any subject group when comparing the different priming conditions (VR, $p's~>~0.343$).

### Using Cohen’s *d*s

Cohen’s *d* can be used with the $p{-}value$ from a common $t-test$ to illustrate if an effect size is not only significant but if a significant result is also relevant. One restriction of this approach is that it is only possible to conduct it for pairwise comparisons, which is not fully in line with the main analyses we provide in the “Results” section. Moreover, t-tests and their p-values are generally presented together with the Cohen’s *d*. The p-values tell if the effect is statistically significant, whereas the Cohen’s *d*s determine the effect size. However, t-tests are usually applied to normally distributed data or in case a dataset is considered to be very large. Nevertheless, we believe that discussing Cohen’s *d*s adds another valuable robustness test for our results. We consider an effect to be (i) small, when the absolute Cohen’s *d* is smaller than 0.2, (ii) medium for absolute Cohen’s *d* between 0.2 and 0.5, and (iii) large if the absolute Cohen’s *d* is larger than 0.5. In the following, we discuss the Cohen’s *d* statistics and add the p-values from respective $t-test$s only for those that report at least a medium Cohen’s *d*.

Supplementary Table 17 summarizes the Cohen’s *d* analyses for competitiveness. When comparing the subject groups in NEUTRAL, we find only small effects (*d* $\in [0.012, 0.101]$). The same is true when pooling by gender or sex in NEUTRAL (*d* $\in [0.021, 0.065]$). The effects sizes for comparing all four subject groups separately between NEUTRAL and FEMININE (*d* $\in [0.031, 0.187]$) and NEUTRAL and MASCULINE (*d* $\in [0.023, 0.115]$) are again small. The only exception are cismen, where the difference between NEUTRAL and MASCULINE becomes medium (*d* $=0.303$) but is insignificant ($p~=~0.072$).

The analyses for risk can be found in Supplementary Table 23. Within NEUTRAL, the effect sizes of comparing cismen or ciswomen with transwomen is medium (*d* $=0.354,0.484$) and only significant for the latter comparison ($p~=~0.011$). Besides, the Cohen’s *d* is getting large and significant for transmen vs. transwomen (*d* $=0.504$; $p~=~0.008$). For all other comparisons, the effects are small between subject groups (*d* $\in [0.017, 0.134]$). Pooling by sex reveals a medium, significant effect size (*d* $=0.268$; $p~=~0.032$) while the effect size for the gender-wise comparison is small (*d* $=0.143$). The effects sizes for each subject group when looking at NEUTRAL vs. FEMININE are small (*d* $\in [0.143, 0.182]$), except for the medium insignificant one of transwomen (*d* $=0.335$; $p~=~0. 107$). For NEUTRAL vs. MASCULINE, cismen show a medium and significant effect size (*d* $=0.348$; $p~=~0.039$), whereas all other subject groups have small or medium, but insignificant Cohen’s *d*s (*d* $\in [0.013, 0.282]$; $p~>~0.097$).

The effect size for the participants’ donations are listed in Supplementary Table 27. They are small and insignificant within NEUTRAL when comparing by sex, gender, or between subject groups (*d* $\in [0.004, 0.098]$; $p~>~0.600$). Similar, the effects sizes for all other comparisons considering the different treatments are small and lack significance (*d* $\in [0.016, 0.188]$; $p~>~0.267$). The only slightly medium and insignificant exception (*d* $=0.210$: $p~=~0.212$) is reported for cismen in NEUTRAL vs. FEMININE.

### Using a continuous instead of a categorical gender measure

With just a handful of exceptions^44,56–58^, researchers in economics always used a categorical way to measure gender. However, it is more and more discussed that gender might be a continuous characteristic rather than a binary (or categorical) one^59^. Techniques accounting for it include asking different questions^60^ or use identity status concerning adherence to actual gender role beliefs^61^. Another method is the BEM sex role inventory^52^. It provides a continuous gender scale, and we conducted it in the post-experimental questionnaire. The BEM is a very accurate predictor for gender and is highly correlated with other continuous gender measures, and single-item measures^58^.

We rerun all regression analyses and include, instead of the subject groups, the variables $BEM score\!: Feminine$ (defined as the score participants reached on the BEM questions measuring femininity) and $BEM score\!: Masculine$ (score on masculine questions in the BEM). Results in Supplementary Tables 28, 30, and 32 show throughout that neither the feminine nor the masculine score significantly influence how the participants decide in NEUTRAL (W, $p's~>~0.057$). This is not surprising since the BEM scores and the gender categories are highly correlated (feminine: KTAU, Kendall’s score = 21692, $p~=~0.001$, masculine: KTAU, Kendall’s score = -18485, $p~=~0.003$), and we did not find correlational gender differences in the baseline condition for neither of the economic decisions we investigate.

Also, for the causal impact of gender priming, no evidence is found for an effect of the BEM score on behavior. Supplementary Tables 29, 31, and 33 confirm this with the insignificant variables measuring the two BEM scores ($p's~>~0.056$), the insignificant interaction terms of the priming condition with the feminine or masculine BEM score ($p's~>~0.054$), and the insignificant respective joint coefficient tests (W, $\chi ^2(1)$/*F*(1), $p's~>~0.108$).

### Controlling for gender congruent upbringing

One limitation of our approach is that the subjects are sorted into distinct gender categories based on their *current* gender identity. This potentially lacks accounting for psychological, behavioral, social, and cultural experiences that shape a gender identity over time, particularly during adolescence. While we can not fully account for this confound, we can analyze if being raised according to one’s current gender affects our primary outcomes.

In our post-experimental questionnaire, we asked the participants according to which gender their parents treated them. Based on the answers and the self-reported gender, we create the variable gender congruent upbringing (GCU). GCU is equal to 1 if someone was raised according to their current gender identity (or was raised neutrally) and 0 otherwise. How the participants were raised matches the currently reported gender of 32.09% of transmen and 15.00% of transwomen. For cisgender individuals, the variable CGU equals 1 for 99.76%. Due to the lack of variation of CGU for the cisgender sample, we conducted all analyses for transgender individuals only.

We rerun all main regression and include, instead of the different subject groups, the variable GCU. Results in Supplementary Tables 43 and 47 show that whether participants were raised according to their current gender does not significantly influence the participants’ competitiveness and altruism in NEUTRAL ($p's~>~0.473$). For risk, we see in Supplementary Table 45 a significantly negative coefficient in NEUTRAL for two out of the three regression) ($p's~<~0.043$). When considering the causal impact of the gender priming, there is again no evidence for an effect of being raised gender-congruent on competitiveness and altruism. Supplementary Tables 44 and 48 show this based on the insignificant coefficient for GCU ($p's~>~0.423$) and the insignificant respective joint coefficient tests (W, $\chi ^2(1)$/*F*(1), $p's~>~0.076$). For risk (see Supplementary Table 46), the coefficients are again significantly negative for NEUTRAL only ($p's~<~0.022$), because the joint coefficient tests taking the treatments and GCU interactions into account remain insignificant (W, *F*(1), $p's~>~0.055$).

### Controlling for the strength of the priming intervention

To underline the strength of our results concerning the priming, we look at the answers to the survey question “Do you remember any of the words from the word-search puzzle? If not, leave empty.”, which was implemented (not incentivized) at the very end of our experiment. We use the outcome of this question to control for the strength of the priming intervention. It can be assumed that the more words a subject remembered, the more they were still primed towards the end of the study. First, 93.08% of all participants remember at least one out of the eight words. The average number of recognized words is 4.33, and 70.00% of all participants reported at least four words. Thus, it can be assumed that the prime was activated for the majority of participants throughout the experiment.

Second, we rerun the regressions in Supplementary Tables 16, 22, and 26 for the three behavioral outcomes. The dummy variables, accounting for the different primings (i.e., the treatment variables), are replaced by $Rem.\,feminine\,words$, $Rem.\,masculine\,words$, and $Rem.\,neutral\,words$, which measure the number of words remembered in each treatment. The only significant and close to significant results found are that cismen in MASCULINE are investing more into the lottery, the more masculine words they remember (see Supplementary Table 35; $p's~<~0.014$) and ciswomen in FEMININE are donating more, the more feminine words they remember (see Supplementary Table 36; $p's~<~0.051$), compared to the NEUTRAL condition. Moreover, we did a subgroup analysis for those who remembered at least the median amount of priming words (i.e., four words) or less (see Supplementary Tables 37 to 42). Overall, when using the remembered words instead of a simple priming variable, our findings in the main “Results” section replicate.

### Correcting for multiple hypothesis testing

Like other scientists, we face the problem of simultaneously evaluating several hypotheses. Conducting multiple comparisons increases the likelihood that a non-negligible proportion of tests are false positives. Thus, drawing valid conclusions requires considering the number of performed statistical tests and adjusting the statistical confidence measures accordingly. We employ the free online tool “Multiple Testing Correction” by^62^, available at www.multipletesting.com.

As we perform with our novel pool of transgender individuals a mix of exploratory and confirmatory analysis, the suitable methods for correction are Bonferroni, the Holm (step-down) approach and the Hochberg (step-up) correction which allows for calculating False Discovery Rates (FDR). According to the Multiple Testing Correction, the first significant *p*-value (values over these thresholds are not considered as significant) is $p~=~0.0015$, independent of the used method. So if we—instead of the significance levels explained in the “Results” section—define a result as significant if it is at least $p~<~0.0015$, all results (non-parametric and findings from regressions) turn out to be insignificant.

## Discussion

This paper applies well-known and extensively used experimental techniques to identify the influence of gender and sex on economic decision-making. First, we separate the impact of gender and sex on economic decisions by collecting data from participants whose gender and sex differ, which is new to the literature. We compare the competitive, risk, and altruistic behavior of four different subject groups—cismen, ciswomen, transmen, and transwomen. Second, we induce either a neutral, feminine, or masculine gender identity by having different priming conditions. Thus, with our experimental setup, we go beyond correlating gender and sex with decisions and try to evoke gender identities through a priming manipulation causally.

While this study is pre-registered and carefully designed following existing literature and the state of the art standards in experimental economics, the findings diverge from previous work. Our results do not show conclusive correlational or causal evidence for gender or sex as determinants of economic decision-making. As described in the main “Results” section, we find just a hand full of significant results. These results do generally not replicate when applying different robustness tests, including accounting for multiple hypothesis testing. Thus, the pattern is essentially consistent: gender and sex differences in behavior remain statistically indistinguishable. Besides, we see that cis- and transgender participants do not systematically differ from each other in their behavior. Our overall interpretation of the data is that gender and sex might not matter as much as initially thought. But what can explain these findings?

First, one explanation could be that gender effects might depend on the underlying subject pool. The existing literature has treated gender differences in behavior as a well-established and robust finding. However, the vast majority of these papers use standard student subjects^63^. Studies that use other samples^64^ or online samples are generally less likely to report gender differences, especially when controlling for a set of participants’ characteristics^18,65^. Moreover, differences in sample size are likely to play a role. We pre-registered a sample that would give us enough statistical power based on existing literature. Still, it remains true that small gender differences in behavior may lie below our minimum detectable effect sizes. The total sample size in this experiment has been constrained by the availability of transgender individuals on Prolific. However, we expect the availability of transgender individuals for future studies to increase, hence allowing for replications of our findings with larger sample sizes.

Second, almost two decades have passed since the first studies that looked into competitiveness, risk, and altruism were published and found gender differences in behavior. One can thus speculate that female empowerment, educational initiatives, and the broader awareness of gender and sex equality in private and professional settings have led to a narrowing of potential behavioral differences in the meantime.

Third, the absence of an effect of gender priming on the behavior of transgender subjects may be rooted in the connotation those subject groups have with gender. For transgender individuals, the concept of gender might be a relatively continuous spectrum whereas for cis-individuals it might be seen as a binary dimension. As such, gender might not be as decisive for transgender as for cisgender individuals. The fact that gender priming seems to work only for cismen but not for ciswomen might hinge on the role gender usually has played for those two subject groups. Whereas for cismen their gender usually comes with advantages and, as such, has a positive connotation, ciswomen might have negative experiences concerning the way society treats them based on their gender.

Despite the partly unexpected findings, we belief that there are several key “takeaways” from this study. For the first time, we present evidence from a sample of cis- and transgender participants in one framework, which allows for both a correlational and a causal approach, and look at how they decide in a competitive context and when making risky or altruistic decisions. Transgender individuals have become a more and more visible part of society. Thus, we think it is crucial to understand their economic behavior. Furthermore, having transgender participants in our sample makes it possible to look deeper into the part that an individual’s gender—as opposed to sex—plays in economic decision-making. In our setting, we shed light on the part of gender effects that can be attributed to biological factors (which refer to a participant’s sex) and other aspects of one’s gender identity. Additionally, we do not measure gender only on a categorical scale; instead, we also apply a continuous gender scale. Our results are qualitatively the same, independent of what gender scale is used. Besides, we use different statistical techniques to analyze our data, which overall point towards the same interpretation of our results. Moreover, we test for the first time if upbringing according to the current gender influences the behavior of transgender individuals. We found that gender-congruent upbringing makes transgender individuals more risk-averse only in the neutral priming condition. For this result, we encourage future research to look into the explanations of this outcome, which would go beyond the original scope of this paper.

Based on our findings, we conclude that the role of gender and sex is not as decisive for economic behavior as previously assumed.

## Supplementary Information

Supplementary Information.

## Data Availability

The dataset generated and analyzed for this research project as well as the custom code that supports the study′s findings are available on OSF (https://osf.io/tyzjh/?view_only=243e2b6ca1174a8d802f496ce97c6a70). The oTree code is available on request.
