# Supplementary material for: On the robustness of gender differences in economic behavior
Source: Sci Rep. 2022 Dec 15;12:21549. doi: 10.1038/s41598-022-25141-1 (PMC9755295; doi:10.1038/s41598-022-25141-1)
Supplement: Supplementary file 1 — Supplementary Information. [file 41598_2022_25141_MOESM1_ESM.pdf]

On the robustness of gender differences in economic behavior
SUPPLEMENTARY INFORMATION

Contents

1 Summary statistics **3**

2 Priming (Part 1) **8**

3 Performance in the real effort math task (Part 2, 3, and 4) **10**

3.1 Performance Part 2 . . . 11

3.2 Performance Part 3 . . . 12

3.3 Performance Part 4 . . . 13

4 Beliefs (Part 3) **15**

4.1 Non-parametric tests . . . 15

4.2 Regressions . . . 16

5 Competitiveness (Part 4) **18**

5.1 Bar graphs . . . 18

5.2 Non-parametric tests . . . 19

5.3 Regressions . . . 20

5.4 Cohen's *d* . . . 22

6 Payoffs (Part 4) **23**

6.1 Regressions . . . 23

7 Risk (Part 5) **25**

7.1 Bar graphs . . . 25

7.2 Non-parametric tests . . . 26

7.3 Regressions . . . 27

7.4 Cohen's *d* . . . 29

8 Altruism (Part 6) **30**

8.1 Bar graphs . . . 30

8.2 Non-parametric tests . . . 31

8.3 Regressions . . . 32

8.4 Cohen's *d* . . . 34

9 Continuous gender measure (BEM) **35**

9.1 Competitiveness . . . 35

9.2 Risk . . . 37

9.3 Altruism . . . 39

10 Controlling for the strength of the primining intervention **41**

10.1 Competitiveness . . . 41

10.2 Risk . . . 42

10.3 Altruism . . . 43

11 Participants remembered at least 4 words **44**

11.1 Competitiveness . . . 44

11.2 Risk . . . 45

11.3 Altruism . . . 46

12 Participants remembered less than 4 words **47**
12.1 Competitiveness . . . 47
12.2 Risk . . . 48
12.3 Altruism . . . 49
13 Gender congruent upbringing **50**
13.1 Competitiveness . . . 50
13.2 Risk . . . 52
13.3 Altruism . . . 54
14 Detailed literature summary **56**
14.1 Competitiveness . . . 56
14.2 Risk . . . 56
14.3 Altruism . . . 57
References . . . 58
15 Additional information **62**
15.1 Study sample . . . 62
15.2 Datasets . . . 62
15.3 Instructions . . . 62

1 Summary statistics

Supplementary Table 1 Descriptives for the cisgender and transgender samples.

		Gender		
	Total	Cisgender	Transgender	p-value
	(N=780)	(N=425)	(N=355)	
Treatment				0.933
NEUTRAL	259 (33.2%)	143 (33.6%)	116 (32.7%)	
FEMININE	263 (33.7%)	141 (33.2%)	122 (34.4%)	
MASCULINE	258 (33.1%)	141 (33.2%)	117 (33.0%)	
Age (years)				0.516
Mean (SD)	24.4 (6.60)	24.3 (6.52)	24.6 (6.71)	
Height (cm)				0.002
Mean (SD)	170 (10.8)	171 (11.0)	169 (10.5)	
Student status				0.830
Yes	368 (47.2%)	202 (47.5%)	166 (46.8%)	
No	412 (52.8%)	223 (52.5%)	189 (53.2%)	
Highest education				0.094
University degree	266 (34.1%)	159 (37.4%)	107 (30.1%)	
High school diploma/A-levels	361 (46.3%)	189 (44.5%)	172 (48.5%)	
Other	153 (19.6%)	77 (18.1%)	76 (21.4%)	
Income: Less than 20,000 GBP				0.171
Yes	541 (69.4%)	286 (67.3%)	255 (71.8%)	
No	239 (30.6%)	139 (32.7%)	100 (28.2%)	
Religion				0.891
Non-religious	547 (70.1%)	295 (69.4%)	252 (71.0%)	
Religious	201 (25.8%)	112 (26.4%)	89 (25.1%)	
Not say	32 (4.1%)	18 (4.2%)	14 (3.9%)	
Residence				<0.001
Continental Europe	250 (32.1%)	169 (39.8%)	81 (22.8%)	
United Kingdom	205 (26.3%)	101 (23.8%)	104 (29.3%)	
United States	265 (34.0%)	133 (31.3%)	132 (37.2%)	
Other	60 (7.7%)	22 (5.2%)	38 (10.7%)	
BEM group:				0.002
Androgynous	188 (24.1%)	116 (27.3%)	72 (20.3%)	
Feminine	222 (28.5%)	104 (24.5%)	118 (33.2%)	
Masculine	151 (19.4%)	95 (22.4%)	56 (15.8%)	
Undifferentiated	219 (28.1%)	110 (25.9%)	109 (30.7%)	
BEM score: Feminine				0.730
Mean (SD)	41.8 (8.58)	41.8 (8.19)	41.7 (9.03)	
BEM score: Masculine				<0.001
Mean (SD)	33.9 (7.95)	35.0 (7.64)	32.5 (8.11)	
TCS				<0.001
Mean (SD)	3.67 (1.14)	4.47 (0.570)	2.71 (0.865)	
STT				<0.001
Mean (SD)	4.35 (4.59)	0.998 (1.47)	8.37 (3.76)	

Note: The table summarizes the characteristics of the cisgender and transgender samples. The education category other includes subjects that replied technical/community college, secondary education (e.g. GED/GCSE), no formal qualification, or don't know/not applicable. The religion category religious includes subjects that replied Buddhism, Christianity, Hinduism, Islam, Judaism, Paganism, Sikhism, or Spiritualism. The residence category other includes subjects that replied Australia, Canada, Chile, Israel, Japan, Mexico, New Zealand, or South Africa. The column p-value reports the p-values of χ^2-tests for categorical variables and the p-values of Wilcoxon-Mann Whitney tests for numerical variables between the cisgender and transgender column.

Supplementary Table 2 Descriptives by treatment for cismen.

	Total	Treatment			p-value
		NEUTRAL	FEMININE	MASCULINE	
**Cismen**					
	(N=214)	(N=72)	(N=71)	(N=71)	
Age (years)					0.042
Mean (SD)	24.1 (5.74)	25.8 (7.70)	24.1 (4.79)	22.5 (3.44)	
Height (cm)					0.449
Mean (SD)	178 (9.08)	180 (10.2)	177 (7.95)	177 (8.77)	
Student status					0.754
Yes	102 (47.7%)	32 (44.4%)	34 (47.9%)	36 (50.7%)	
No	112 (52.3%)	40 (55.6%)	37 (52.1%)	35 (49.3%)	
Highest education					0.237
University degree	72 (33.6%)	26 (36.1%)	27 (38.0%)	19 (26.8%)	
High school diploma/A-levels	94 (43.9%)	27 (37.5%)	28 (39.4%)	39 (54.9%)	
Other	48 (22.4%)	19 (26.4%)	16 (22.5%)	13 (18.3%)	
Income: Less than 20,000 GBP					0.841
Yes	135 (63.1%)	47 (65.3%)	43 (60.6%)	45 (63.4%)	
No	79 (36.9%)	25 (34.7%)	28 (39.4%)	26 (36.6%)	
Religion					0.820
Non-religious	144 (67.3%)	48 (66.7%)	47 (66.2%)	49 (69.0%)	
Religious	60 (28.0%)	21 (29.2%)	19 (26.8%)	20 (28.2%)	
Not say	10 (4.7%)	3 (4.2%)	5 (7.0%)	2 (2.8%)	
Residence					0.972
Continental Europe	95 (44.4%)	31 (43.1%)	30 (42.3%)	34 (47.9%)	
United Kingdom	50 (23.4%)	17 (23.6%)	17 (23.9%)	16 (22.5%)	
United States	65 (30.4%)	23 (31.9%)	23 (32.4%)	19 (26.8%)	
Other	4 (1.9%)	1 (1.4%)	1 (1.4%)	2 (2.8%)	
BEM group:					0.490
Androgynous	56 (26.2%)	19 (26.4%)	19 (26.8%)	18 (25.4%)	
Feminine	40 (18.7%)	15 (20.8%)	11 (15.5%)	14 (19.7%)	
Masculine	59 (27.6%)	22 (30.6%)	15 (21.1%)	22 (31.0%)	
Undifferentiated	59 (27.6%)	16 (22.2%)	26 (36.6%)	17 (23.9%)	
BEM score: Feminine					0.644
Mean (SD)	40.0 (8.55)	40.4 (8.52)	39.3 (9.13)	40.4 (8.03)	
BEM score: Masculine					0.522
Mean (SD)	35.7 (7.68)	36.2 (6.93)	34.9 (7.71)	36.0 (8.41)	
TCS					0.620
Mean (SD)	4.47 (0.591)	4.45 (0.541)	4.49 (0.590)	4.46 (0.645)	
STT					0.001
Mean (SD)	0.986 (1.46)	1.18 (1.09)	0.535 (0.939)	1.24 (2.01)	

Note: The table summarizes the characteristics of the cisgender and transgender samples. The education category other includes subjects that replied technical/community college, secondary education (e.g. GED/GCSE), no formal qualification, or don't know/not applicable. The religion category religious includes subjects that replied Buddhism, Christianity, Hinduism, Islam, Judaism, Paganism, Sikhism, or Spiritualism. The residence category other includes subjects that replied Australia, Canada, Chile, Israel, Japan, Mexico, New Zealand, or South Africa. The column p-value reports the p-values of χ^2-tests for categorical variables and the p-values of the Kruskal Wallis test for numerical variables between the treatment columns.

Supplementary Table 3 Descriptives by treatment for ciswomen.

	Total	Treatment: NEUTRAL	FEMININE	MASCULINE	p-value
**Ciswomen**					
	(N=211)	(N=71)	(N=70)	(N=70)	
Age (years)					0.644
Mean (SD)	24.6 (7.23)	25.0 (7.83)	25.1 (7.79)	23.6 (5.90)	
Height (cm)					0.541
Mean (SD)	164 (7.96)	164 (9.88)	164 (7.02)	165 (6.60)	
Student status					0.813
Yes	100 (47.4%)	35 (49.3%)	34 (48.6%)	31 (44.3%)	
No	111 (52.6%)	36 (50.7%)	36 (51.4%)	39 (55.7%)	
Highest education					0.667
University degree	87 (41.2%)	32 (45.1%)	31 (44.3%)	24 (34.3%)	
High school diploma/A-levels	95 (45.0%)	29 (40.8%)	31 (44.3%)	35 (50.0%)	
Other	29 (13.7%)	10 (14.1%)	8 (11.4%)	11 (15.7%)	
Income: Less than 20,000 GBP					0.253
Yes	151 (71.6%)	53 (74.6%)	53 (75.7%)	45 (64.3%)	
No	60 (28.4%)	18 (25.4%)	17 (24.3%)	25 (35.7%)	
Religion					0.990
Non-religious	151 (71.6%)	51 (71.8%)	50 (71.4%)	50 (71.4%)	
Religious	52 (24.6%)	17 (23.9%)	17 (24.3%)	18 (25.7%)	
Not say	8 (3.8%)	3 (4.2%)	3 (4.3%)	2 (2.9%)	
Residence					0.589
Continental Europe	74 (35.1%)	28 (39.4%)	25 (35.7%)	21 (30.0%)	
United Kingdom	51 (24.2%)	19 (26.8%)	15 (21.4%)	17 (24.3%)	
United States	68 (32.2%)	21 (29.6%)	24 (34.3%)	23 (32.9%)	
Other	18 (8.5%)	3 (4.2%)	6 (8.6%)	9 (12.9%)	
BEM group:					0.187
Androgynous	60 (28.4%)	17 (23.9%)	28 (40.0%)	15 (21.4%)	
Feminine	64 (30.3%)	23 (32.4%)	19 (27.1%)	22 (31.4%)	
Masculine	36 (17.1%)	14 (19.7%)	11 (15.7%)	11 (15.7%)	
Undifferentiated	51 (24.2%)	17 (23.9%)	12 (17.1%)	22 (31.4%)	
BEM score: Feminine					0.212
Mean (SD)	43.5 (7.42)	43.3 (7.35)	44.7 (7.66)	42.6 (7.22)	
BEM score: Masculine					0.099
Mean (SD)	34.3 (7.54)	33.9 (7.31)	35.9 (8.18)	33.2 (6.93)	
TCS					0.878
Mean (SD)	4.48 (0.550)	4.55 (0.413)	4.47 (0.585)	4.42 (0.630)	
STT					0.906
Mean (SD)	1.01 (1.49)	1.15 (2.07)	0.957 (1.04)	0.914 (1.14)	

Note: The table summarizes the characteristics of the cisgender and transgender samples. The education category other includes subjects that replied technical/community college, secondary education (e.g. GED/GCSE), no formal qualification, or don't know/not applicable. The religion category religious includes subjects that replied Buddhism, Christianity, Hinduism, Islam, Judaism, Paganism, Sikhism, or Spiritualism. The residence category other includes subjects that replied Australia, Canada, Chile, Israel, Japan, Mexico, New Zealand, or South Africa. The column p-value reports the p-values of χ^2-tests for categorical variables and the p-values of the Kruskal Wallis test for numerical variables between the treatment columns.

Supplementary Table 4 Descriptives by treatment for transmen.

	Total	Treatment			*p*-value
		NEUTRAL	FEMININE	MASCULINE	
**Transmen**					
	(N=215)	(N=72)	(N=72)	(N=71)	
Age (years)					0.775
Mean (SD)	24.3 (6.40)	25.1 (8.07)	24.0 (5.61)	23.7 (5.12)	
Height (cm)					0.301
Mean (SD)	164 (8.52)	165 (10.4)	164 (7.32)	164 (7.64)	
Student status					0.376
Yes	108 (50.2%)	34 (47.2%)	41 (56.9%)	33 (46.5%)	
No	107 (49.8%)	38 (52.8%)	31 (43.1%)	38 (53.5%)	
Highest education					0.891
University degree	63 (29.3%)	22 (30.6%)	19 (26.4%)	22 (31.0%)	
High school diploma/A-levels	109 (50.7%)	37 (51.4%)	39 (54.2%)	33 (46.5%)	
Other	43 (20.0%)	13 (18.1%)	14 (19.4%)	16 (22.5%)	
Income: Less than 20,000 GBP					0.355
Yes	155 (72.1%)	48 (66.7%)	52 (72.2%)	55 (77.5%)	
No	60 (27.9%)	24 (33.3%)	20 (27.8%)	16 (22.5%)	
Religion					0.892
Non-religious	144 (67.0%)	47 (65.3%)	49 (68.1%)	48 (67.6%)	
Religious	62 (28.8%)	23 (31.9%)	19 (26.4%)	20 (28.2%)	
Not say	9 (4.2%)	2 (2.8%)	4 (5.6%)	3 (4.2%)	
Residence					0.939
Continental Europe	47 (21.9%)	14 (19.4%)	17 (23.6%)	16 (22.5%)	
United Kingdom	64 (29.8%)	22 (30.6%)	23 (31.9%)	19 (26.8%)	
United States	85 (39.5%)	31 (43.1%)	25 (34.7%)	29 (40.8%)	
Other	19 (8.8%)	5 (6.9%)	7 (9.7%)	7 (9.9%)	
BEM group:					0.927
Androgynous	44 (20.5%)	17 (23.6%)	13 (18.1%)	14 (19.7%)	
Feminine	69 (32.1%)	20 (27.8%)	24 (33.3%)	25 (35.2%)	
Masculine	42 (19.5%)	16 (22.2%)	13 (18.1%)	13 (18.3%)	
Undifferentiated	60 (27.9%)	19 (26.4%)	22 (30.6%)	19 (26.8%)	
BEM score: Feminine					0.809
Mean (SD)	41.4 (8.90)	41.1 (8.18)	41.5 (9.47)	41.6 (9.13)	
BEM score: Masculine					0.597
Mean (SD)	33.6 (7.43)	34.0 (7.42)	33.1 (6.98)	33.6 (7.95)	
TCS					0.692
Mean (SD)	2.82 (0.868)	2.88 (0.946)	2.75 (0.857)	2.84 (0.800)	
STT					0.910
Mean (SD)	9.26 (3.15)	9.29 (3.50)	9.21 (3.01)	9.27 (2.96)	

Note: The table summarizes the characteristics of the cisgender and transgender samples. The education category other includes subjects that replied technical/community college, secondary education (e.g. GED/GCSE), no formal qualification, or don't know/not applicable. The religion category religious includes subjects that replied Buddhism, Christianity, Hinduism, Islam, Judaism, Paganism, Sikhism, or Spiritualism. The residence category other includes subjects that replied Australia, Canada, Chile, Israel, Japan, Mexico, New Zealand, or South Africa. The column *p*-value reports the *p*-values of χ^2-tests for categorical variables and the *p*-values of the Kruskal Wallis test for numerical variables between the treatment columns.

Supplementary Table 5 Descriptives by treatment for transwomen.

		Treatment			
	Total	NEUTRAL	FEMININE	MASCULINE	p-value
**Transwomen**					
	(N=140)	(N=44)	(N=50)	(N=46)	
Age (years)					0.345
Mean (SD)	25.1 (7.15)	25.3 (5.91)	25.6 (9.01)	24.2 (5.89)	
Height (cm)					0.864
Mean (SD)	175 (10.1)	176 (8.41)	174 (13.4)	175 (7.24)	
Student status					0.939
Yes	58 (41.4%)	18 (40.9%)	20 (40.0%)	20 (43.5%)	
No	82 (58.6%)	26 (59.1%)	30 (60.0%)	26 (56.5%)	
Highest education					0.090
University degree	44 (31.4%)	13 (29.5%)	20 (40.0%)	11 (23.9%)	
High school diploma/A-levels	63 (45.0%)	16 (36.4%)	20 (40.0%)	27 (58.7%)	
Other	33 (23.6%)	15 (34.1%)	10 (20.0%)	8 (17.4%)	
Income: Less than 20,000 GBP					0.070
Yes	100 (71.4%)	37 (84.1%)	34 (68.0%)	29 (63.0%)	
No	40 (28.6%)	7 (15.9%)	16 (32.0%)	17 (37.0%)	
Religion					0.664
Non-religious	108 (77.1%)	33 (75.0%)	39 (78.0%)	36 (78.3%)	
Religious	27 (19.3%)	10 (22.7%)	10 (20.0%)	7 (15.2%)	
Not say	5 (3.6%)	1 (2.3%)	1 (2.0%)	3 (6.5%)	
Residence					0.257
Continental Europe	34 (24.3%)	11 (25.0%)	9 (18.0%)	14 (30.4%)	
United Kingdom	40 (28.6%)	11 (25.0%)	17 (34.0%)	12 (26.1%)	
United States	47 (33.6%)	13 (29.5%)	21 (42.0%)	13 (28.3%)	
Other	19 (13.6%)	9 (20.5%)	3 (6.0%)	7 (15.2%)	
BEM group:					0.333
Androgynous	28 (20.0%)	5 (11.4%)	12 (24.0%)	11 (23.9%)	
Feminine	49 (35.0%)	19 (43.2%)	17 (34.0%)	13 (28.3%)	
Masculine	14 (10.0%)	7 (15.9%)	3 (6.0%)	4 (8.7%)	
Undifferentiated	49 (35.0%)	13 (29.5%)	18 (36.0%)	18 (39.1%)	
BEM score: Feminine					0.973
Mean (SD)	42.3 (9.22)	42.8 (7.72)	41.3 (10.4)	42.8 (9.28)	
BEM score: Masculine					0.996
Mean (SD)	30.9 (8.83)	31.0 (9.17)	30.8 (8.75)	30.9 (8.80)	
TCS					0.745
Mean (SD)	2.54 (0.835)	2.54 (0.755)	2.55 (0.872)	2.52 (0.883)	
STT					0.027
Mean (SD)	7.01 (4.19)	7.93 (4.05)	5.80 (4.35)	7.46 (3.91)	

Note: The table summarizes the characteristics of the cisgender and transgender samples. The education category other includes subjects that replied technical/community college, secondary education (e.g. GED/GCSE), no formal qualification, or don't know/not applicable. The religion category religious includes subjects that replied Buddhism, Christianity, Hinduism, Islam, Judaism, Paganism, Sikhism, or Spiritualism. The residence category other includes subjects that replied Australia, Canada, Chile, Israel, Japan, Mexico, New Zealand, or South Africa. The column p-value reports the p-values of χ^2-tests for categorical variables and the p-values of the Kruskal Wallis test for numerical variables between the treatment columns.

2 Priming (Part 1)

Figure 1 presents the number of marked words split up by treatments and subject groups. We do not find any differences in marked words within one priming condition across subject groups (KW; NEUTRAL: $p = 0.349$; FEMININE: $p = 0.874$; MASCULINE: $p = 0.112$). For the different subject groups separately across priming conditions, only the number of words marked by transmen didn't differ across priming conditions (KW; cismen: $p < 0.000$; ciswomen: $p = 0.038$; transmen: $p = 0.123$; transwomen: $p = 0.014$). Concerning gender differences, we do not see significant variations (MWU; $p = 0.675$). The same is true for sex difference (MWU; $p < 0.060$). As we did not pre-register to control for the number of words marked in our regressions, we do not add this variable in the reported analysis. However, please note that all main results remain qualitatively the same when we account for the heterogeneity in the number of marked words. The additional analyses are available on request.

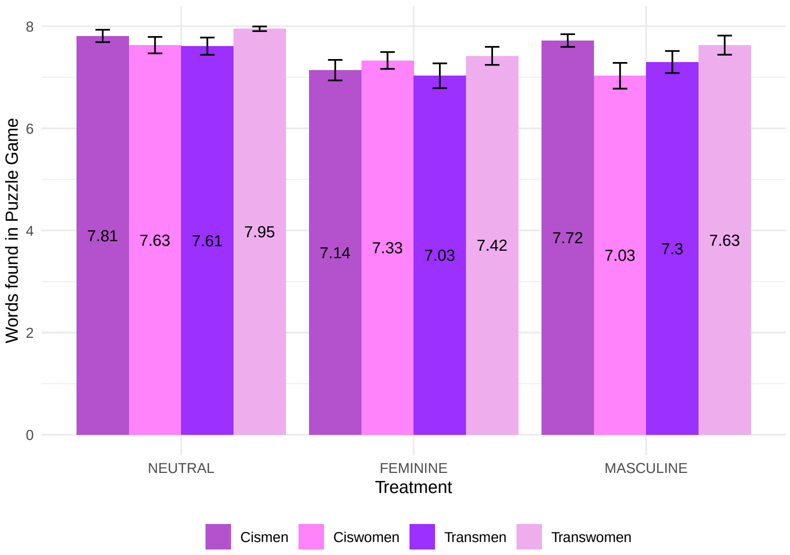

Supplementary Figure 1 Marked words in Part 1 by treatments and subject groups in alphabetical order ($n = 780$). The bars show the average amount of marked words, and the error bars represent the standard error of the mean.

Supplementary Table 6 Words found in the priming task across treatments and subject groups.

Panel A: Priming across treatments

	Treatment			
Subject groups	NEUTRAL	FEMININE	MASCULINE	p-value
Cismen	7.806	7.141	7.718	<0.001
Ciswomen	7.634	7.329	7.029	0.038
Transmen	7.611	7.028	7.296	0.123
Transwomen	7.955	7.420	7.630	0.014

Panel B: Priming across subject groups

	Subject groups				
Treatment	Cismen	Ciswomen	Transmen	Transwomen	p-value
NEUTRAL	7.806	7.634	7.611	7.955	0.349
FEMININE	7.141	7.329	7.028	7.420	0.874
MASCULINE	7.718	7.029	7.296	7.630	0.112

Panel C: Priming across groups within NEUTRAL

	Group 1		Group 2		
	Subjects		Subjects		p-value
Case 1	Cisgender	7.720	Transgender	7.741	0.816
Case 2	Cismen	7.806	Ciswomen	7.634	0.339
Case 3	Transmen	7.611	Transwomen	7.955	0.122
Case 4	Female	7.622	Male	7.862	0.091
Case 5	Feminine	7.757	Masculine	7.708	0.820

Panel D: Priming in NEUTRAL compared to the other treatments

	NEUTRAL	FEMININE		MASCULINE	
Subject groups			p-value		p-value
Cismen	7.806	7.141	<0.001	7.718	0.345
Ciswomen	7.634	7.329	0.012	7.029	0.035
Transmen	7.611	7.028	0.040	7.296	0.207
Transwomen	7.955	7.420	0.004	7.630	0.100

Note: The columns p-value report the results of the tests performed column-wise. For continuous variables, we conducted Mann-Whitney U tests for two groups and Kruskal-Wallis tests for more than two groups.

3 Performance in the real effort math task (Part 2, 3, and 4)

The following tables summarize the performance in the math task by treatment and subject groups for Part 2 (Table 7) and Part 3 (Table 8). By treatments, ciswomen and cismen have differences in performance in MASCULINE in Part 2 (MWU; NEUTRAL: $p = 0.080$, FEMININE: $p = 0.205$, MASCULINE: $p = 0.037$) and across all treatments in Part 3 (MWU; NEUTRAL: $p = 0.004$, Part 3 FEMININE: $p = 0.010$, Part 3 MASCULINE: $p = 0.028$). Also, the paper[1] (which set up the online version of this math task) does find gender differences in performance. Thomas Buser states in a personal communication that women "perform significantly worse". In their paper, this is true for their first-round (our Part 2), where "women score 1.3 fewer correct answers" than men (male average: 10.0). Moreover, in their second-round (our Part 4), males score on average 10.0, but women score 0.7 fewer correct answers.

Transgender participants show performance differences in NEUTRAL in Part 2 and 3 (MWU; Part 2: NEUTRAL: $p = 0.007$, FEMININE: $p = 0.555$, MASCULINE: $p = 0.181$; Part 3: NEUTRAL: $p = 0.015$, FEMININE: $p = 0.600$, MASCULINE: $p = 0.053$). Concerning sex differences, male participants always have a higher performance than female ones in NEUTRAL and MASCULINE when facing piece-rate incentives (MWU; Part 2: NEUTRAL: $p = 0.003$, FEMININE: $p = 0.164$, MASCULINE: $p = 0.010$). Interestingly, this is true when they compete in Part 3 for all three treatments (MWU; NEUTRAL: $p < 0.001$, FEMININE: $p = 0.014$, MASCULINE: $p = 0.003$).

However, performances do not differ by the individual's gender (MWU; Part 2: NEUTRAL: $p = 0.755$, FEMININE: $p = 0.621$, MASCULINE: $p = 0.553$; Part 3: NEUTRAL: $p = 0.575$, FEMININE: $p = 0.161$, MASCULINE: $p = 0.675$). All differences vanish in Part 4, when we split up the data by those in the tournament (see the respective p-values in Table 9 and Table 9). For the priming intervention, we have no evidence of priming influencing the performance, independent of the part or subject group (KW; cismen: $p > 0.478$; ciswomen: $p > 0.562$; transmen: $p > 0.956$; or transwomen: $p > 0.170$).

Please note that we can not exclude that the math task is not influenced by a participant's gender and sex, combinations of it, in addition to interactions with priming. However, we can control how performance heterogeneity affects competitiveness by adding individual performances to our regressions measuring competitiveness. See Table 15 to Table 16.

3.1 Performance Part 2

Supplementary Table 7 Performance in Part 2 across treatments and subject groups.

Panel A: Performance in Part 2 across treatments

Subject groups	Treatment NEUTRAL	FEMININE	MASCULINE	p-value
Cismen	8.458	8.704	9.423	0.478
Ciswomen	7.535	7.929	8.014	0.719
Transmen	7.750	7.778	7.718	0.956
Transwomen	9.409	8.740	8.739	0.285

Panel B: Performance in Part 2 across subject groups

Treatment	Subject groups Cismen	Ciswomen	Transmen	Transwomen	p-value
NEUTRAL	8.458	7.535	7.750	9.409	0.011
FEMININE	8.704	7.929	7.778	8.740	0.529
MASCULINE	9.423	8.014	7.718	8.739	0.062

Panel C: Performance in Part 2 across groups within NEUTRAL

	Group 1		Group 2		
	Subjects		Subjects		p-value
Case 1	Cisgender	8.000	Transgender	8.379	0.405
Case 2	Cismen	8.458	Ciswomen	7.535	0.080
Case 3	Transmen	7.750	Transwomen	9.409	0.007
Case 4	Female	7.643	Male	8.819	0.003
Case 5	Feminine	8.252	Masculine	8.104	0.755

Panel D: Performance in Part 2 in NEUTRAL compared to the other treatments

	NEUTRAL	FEMININE		MASCULINE	
Subject groups			p-value		p-value
Cismen	8.458	8.704	0.797	9.423	0.231
Ciswomen	7.535	7.929	0.488	8.014	0.476
Transmen	7.750	7.778	0.832	7.718	0.932
Transwomen	9.409	8.740	0.125	8.739	0.255

Note: The columns p-value report the results of the tests performed column-wise. For continuous variables, we conducted Mann-Whitney U tests for two groups and Kruskal-Wallis tests for more than two groups.

3.2 Performance Part 3

Supplementary Table 8 Performance in Part 3 across treatments and subject groups.

Panel A: Performance in Part 3 across treatments

Subject groups	Treatment: NEUTRAL	FEMININE	MASCULINE	p-value
Cismen	9.333	10.070	9.930	0.593
Ciswomen	7.423	7.957	8.271	0.562
Transmen	7.833	7.736	7.930	0.979
Transwomen	9.659	8.500	9.326	0.170

Panel B: Performance in Part 3 across subject groups

Treatment	Subject groups: Cismen	Ciswomen	Transmen	Transwomen	p-value
NEUTRAL	9.333	7.423	7.833	9.659	0.002
FEMININE	10.070	7.957	7.736	8.500	0.021
MASCULINE	9.930	8.271	7.930	9.326	0.024

Panel C: Performance in Part 3 across groups within NEUTRAL

	Group 1		Group 2		
	Subjects		Subjects		p-value
Case 1	Cisgender	8.385	Transgender	8.526	0.612
Case 2	Cismen	9.333	Ciswomen	7.423	0.004
Case 3	Transmen	7.833	Transwomen	9.659	0.015
Case 4	Female	7.629	Male	9.457	<0.001
Case 5	Feminine	8.278	Masculine	8.583	0.575

Panel D: Performance in Part 3 in NEUTRAL compared to the other treatments

	NEUTRAL	FEMININE		MASCULINE	
Subject groups			p-value		p-value
Cismen	9.333	10.070	0.353	9.930	0.406
Ciswomen	7.423	7.957	0.396	8.271	0.325
Transmen	7.833	7.736	0.920	7.930	0.832
Transwomen	9.659	8.500	0.081	9.326	0.686

Note: The columns p-value report the results of the tests performed column-wise. For continuous variables, we conducted Mann-Whitney U tests for two groups and Kruskal-Wallis tests for more than two groups.

3.3 Performance Part 4

Supplementary Table 9 Performance in Part 4 of *competing* subjects across treatments and subject groups.

Panel A: Performance in Part 4 of competing subjects across treatments

	Treatment			
Subject groups	NEUTRAL	FEMININE	MASCULINE	p-value
Cismen	9.500	10.321	9.312	0.765
Ciswomen	8.391	8.947	9.053	0.763
Transmen	9.000	8.211	8.474	0.839
Transwomen	9.429	8.333	9.167	0.670

Panel B: Performance in Part 4 of competing subjects across subject groups

	Subject groups				
Treatment	Cismen	Ciswomen	Transmen	Transwomen	p-value
NEUTRAL	9.500	8.391	9.000	9.429	0.705
FEMININE	10.321	8.947	8.211	8.333	0.389
MASCULINE	9.312	9.053	8.474	9.167	0.923

Panel C: Performance in Part 4 of competing subjects across groups within NEUTRAL

	Group 1		Group 2		
	Subjects		Subjects		p-value
Case 1	Cisgender	8.933	Transgender	9.176	0.651
Case 2	Cismen	9.500	Ciswomen	8.391	0.278
Case 3	Transmen	9.000	Transwomen	9.429	0.860
Case 4	Female	8.674	Male	9.472	0.358
Case 5	Feminine	8.784	Masculine	9.262	0.475

Panel D: Performance in Part 4 of competing subjects in NEUTRAL compared to the other treatments

	NEUTRAL	FEMININE		MASCULINE	
Subject groups			p-value		p-value
Cismen	9.500	10.321	0.556	9.312	0.965
Ciswomen	8.391	8.947	0.638	9.053	0.494
Transmen	9.000	8.211	0.563	8.474	0.671
Transwomen	9.429	8.333	0.360	9.167	0.797

Note: The columns p-value report the results of the tests performed column-wise. For continuous variables, we conducted Mann-Whitney U tests for two groups and Kruskal-Wallis tests for more than two groups.

Supplementary Table 10 Performance in Part 4 of *non-competing* subjects across treatments and subject groups.

Panel A: Performance in Part 4 of non-competing subjects across treatments

	Treatment			
Subject groups	NEUTRAL	FEMININE	MASCULINE	p-value
Cismen	8.960	9.093	9.026	0.994
Ciswomen	7.979	9.098	8.725	0.341
Transmen	8.712	8.453	8.288	0.825
Transwomen	8.800	8.906	9.500	0.703

Panel B: Performance in Part 4 of non-competing subjects across subject groups

	Subject groups				
Treatment	Cismen	Ciswomen	Transmen	Transwomen	p-value
NEUTRAL	8.960	7.979	8.712	8.800	0.577
FEMININE	9.093	9.098	8.453	8.906	0.929
MASCULINE	9.026	8.725	8.288	9.500	0.376

Panel C: Performance in Part 4 of non-competing subjects across groups within NEUTRAL

	Group 1		Group 2		
	Subjects		Subjects		p-value
Case 1	Cisgender	8.480	Transgender	8.744	0.874
Case 2	Cismen	8.960	Ciswomen	7.979	0.159
Case 3	Transmen	8.712	Transwomen	8.800	0.977
Case 4	Female	8.360	Male	8.900	0.292
Case 5	Feminine	8.295	Masculine	8.833	0.309

Panel D: Performance in Part 4 of non-competing subjects in NEUTRAL compared to the other treatments

	NEUTRAL	FEMININE		MASCULINE	
Subject groups			p-value		p-value
Cismen	8.960	9.093	0.914	9.026	0.960
Ciswomen	7.979	9.098	0.135	8.725	0.529
Transmen	8.712	8.453	0.921	8.288	0.606
Transwomen	8.800	8.906	0.843	9.500	0.445

Note: The columns p-value report the results of the tests performed column-wise. For continuous variables, we conducted Mann-Whitney U tests for two groups and Kruskal-Wallis tests for more than two groups.

4 Beliefs (Part 3)

4.1 Non-parametric tests

Supplementary Table 11 Beliefs in Part 3 across treatments and subject groups.

Panel A: Beliefs in Part 3 across treatments

	Treatment			
Subject groups	NEUTRAL	FEMININE	MASCULINE	p-value
Cismen	2.139	1.944	2.070	0.391
Ciswomen	2.606	2.500	2.571	0.793
Transmen	2.542	2.653	2.704	0.633
Transwomen	2.205	2.440	2.304	0.396

Panel B: Beliefs in Part 3 across subject groups

	Subject groups				
Treatment	Cismen	Ciswomen	Transmen	Transwomen	p-value
NEUTRAL	2.139	2.606	2.542	2.205	0.006
FEMININE	1.944	2.500	2.653	2.440	<0.001
MASCULINE	2.070	2.571	2.704	2.304	<0.001

Panel C: Beliefs in Part 3 across groups within NEUTRAL

	Group 1		Group 2		
	Subjects		Subjects		p-value
Case 1	Cisgender	2.371	Transgender	2.414	0.746
Case 2	Cismen	2.139	Ciswomen	2.606	0.003
Case 3	Transmen	2.542	Transwomen	2.205	0.061
Case 4	Female	2.573	Male	2.164	0.001
Case 5	Feminine	2.452	Masculine	2.340	0.362

Panel D: Beliefs in Part 3 in NEUTRAL compared to the other treatments

	NEUTRAL	FEMININE		MASCULINE	
Subject groups			p-value		p-value
Cismen	2.139	1.944	0.177	2.070	0.567
Ciswomen	2.606	2.500	0.496	2.571	0.772
Transmen	2.542	2.653	0.529	2.704	0.354
Transwomen	2.205	2.440	0.178	2.304	0.537

Note: The columns p-value report the results of the tests performed column-wise. For continuous variables, we conducted Mann-Whitney U tests for two groups and Kruskal-Wallis tests for more than two groups.

4.2 Regressions

Supplementary Table 12 OLS regression for NEUTRAL. Dependent variable: Beliefs in Part 3.

	(1)	(2)	(3)
Ciswomen	0.467 **	0.586 **	0.494 **
	(0.155)	(0.176)	(0.156)
Transmen	0.403 *	0.511 **	-0.155
	(0.159)	(0.180)	(0.322)
Transwomen	0.066	0.194	-0.551
	(0.172)	(0.173)	(0.324)
Age		0.000	
		(0.009)	
Height		0.005	
		(0.006)	
Student status		-0.169	
		(0.136)	
Income: < 20,000 GBP		-0.077	
		(0.135)	
Religion: Religious		0.243	
		(0.135)	
Religion: Not say		0.460	
		(0.313)	
Residence: US		-0.080	
		(0.152)	
Residence: UK		0.097	
		(0.170)	
Residence: Other		-0.408 *	
		(0.202)	
TCS			-0.253 **
			(0.093)
STT			0.020
			(0.022)
Const.	2.139 ***	1.226	3.242 ***
	(0.105)	(1.195)	(0.436)
N	259	259	259
Adj. R2	0.035	0.057	0.057
H_0: Sex	0.001	0.001	0.000
H_0: Gender	0.587	0.259	0.682

Note: The beliefs in Part 3 are the participants belief about how their performance ranks within the group (1 = best to 4 = worst). Standard errors in parentheses are heteroskedasticity robust. In the second last column from the right, the baseline is a non-student, non-religious cisman, who earns more than 20K GBP, and lives in continental Europe. In the last column from the right, the baseline is a cisman. *** $p < 0.001$; ** $p < 0.01$; * $p < 0.05$. H_0: Sex reports the p-values of a joint coefficient test comparing Male (Cismen and Transwomen) with Female (Ciswomen and Transmen). H_0: Gender reports the p-values of a joint coefficient test comparing Masculine (Cismen and Transmen) with Feminine (Ciswomen and Transwomen).

Supplementary Table 13 OLS regression for all treatments. Dependent variable: Beliefs in Part 3.

	(1)	(2)	(3)
Ciswomen	0.467 **	0.444 **	0.484 **
	(0.155)	(0.162)	(0.155)
Transmen	0.403 *	0.405 *	0.093
	(0.159)	(0.164)	(0.229)
Transwomen	0.066	0.080	-0.291
	(0.172)	(0.174)	(0.234)
Treatment: FEMININE	-0.195	-0.187	-0.184
	(0.149)	(0.146)	(0.148)
Treatment: MASCULINE	-0.068	-0.045	-0.067
	(0.151)	(0.148)	(0.150)
FEMININE x Ciswomen	0.090	0.094	0.065
	(0.218)	(0.216)	(0.217)
MASCULINE x Ciswomen	0.034	0.034	0.013
	(0.220)	(0.215)	(0.219)
FEMININE x Transmen	0.306	0.309	0.273
	(0.218)	(0.216)	(0.215)
MASCULINE x Transmen	0.231	0.210	0.222
	(0.222)	(0.220)	(0.219)
FEMININE x Transwomen	0.431	0.421	0.434
	(0.239)	(0.238)	(0.236)
MASCULINE x Transwomen	0.168	0.145	0.166
	(0.242)	(0.242)	(0.241)
Const.	2.139 ***	2.547 ***	2.860 ***
	(0.105)	(0.693)	(0.250)
Controls (Age, Height, Student status, Income, Religion, Residence)	–	Yes	–
Controls (TCS, STT)	–	–	Yes
N	780	780	780
Adj. R2	0.055	0.074	0.067
H_0: FEMININE on Cismen	0.192	0.201	0.213
H_0: MASCULINE on Cismen	0.651	0.762	0.654
H_0: FEMININE on Ciswomen	0.504	0.553	0.454
H_0: MASCULINE on Ciswomen	0.830	0.943	0.736
H_0: FEMININE on Transmen	0.483	0.443	0.568
H_0: MASCULINE on Transmen	0.317	0.309	0.331
H_0: FEMININE on Transwomen	0.207	0.211	0.178
H_0: MASCULINE on Transwomen	0.597	0.601	0.599

Note: The beliefs in Part 3 are the participants belief about how their performance ranks within the group (1 = best to 4 = worst). Standard errors in parentheses are heteroskedasticity robust. In the second last column from the right, the baseline is a non-student, non-religious cisman, who earns more than 20K GBP, and lives in continental Europe. In the last column from the right, the baseline is a cisman. *** $p < 0.001$; ** $p < 0.01$; * $p < 0.05$. Rows starting with H_0 report the p-values of a joint coefficient test that the coefficients' sum equals 0. For example, "H_0: FEMININE on Ciswomen" tests the effect of the treatment (FEMININE) on the subject group (Ciswomen).

5 Competitiveness (Part 4)

5.1 Bar graphs

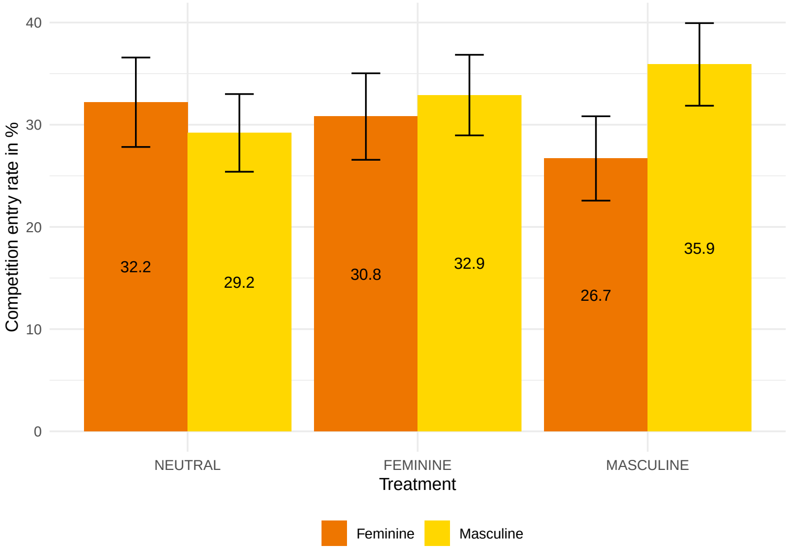

Supplementary Figure 2 Tournament entry rates in Part 4 by treatments and gender ($n = 780$). The bars show the percentage of participants (between 0 and 100) who chose to compete rather than to perform under piece-rate incentives. The error bars represent the standard error of the mean.

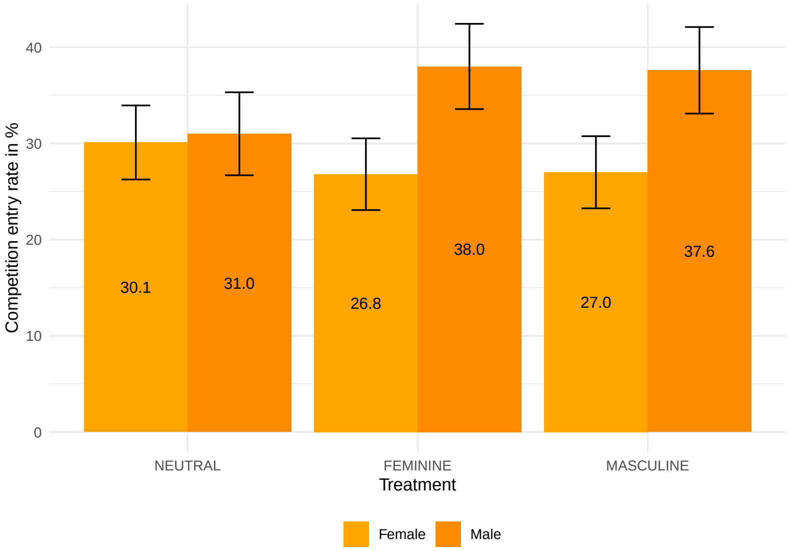

Supplementary Figure 3 Tournament entry rates in Part 4 by treatments and sex ($n = 780$). The bars show the percentage of participants (between 0 and 100) who chose to compete rather than to perform under piece-rate incentives. The error bars represent the standard error of the mean.

5.2 Non-parametric tests

Supplementary Table 14 Tournament entry rates across treatments and subject groups.

Panel A: Competitiveness across treatments

	Treatment			
Subject groups	NEUTRAL	FEMININE	MASCULINE	p-value
Cismen	30.6%	39.4%	45.1%	0.198
Ciswomen	32.4%	27.1%	27.1%	0.729
Transmen	27.8%	26.4%	26.8%	0.981
Transwomen	31.8%	36.0%	26.1%	0.578

Panel B: Competitiveness across subject groups

	Subject groups				
Treatment	Cismen	Ciswomen	Transmen	Transwomen	p-value
NEUTRAL	30.6%	32.4%	27.8%	31.8%	0.939
FEMININE	39.4%	27.1%	26.4%	36.0%	0.264
MASCULINE	45.1%	27.1%	26.8%	26.1%	0.046

Panel C: Competitiveness across groups within NEUTRAL

	Group 1		Group 2		
	Subjects		Subjects		p-value
Case 1	Cisgender	31.5%	Transgender	29.3%	0.708
Case 2	Cismen	30.6%	Ciswomen	32.4%	0.813
Case 3	Transmen	27.8%	Transwomen	31.8%	0.643
Case 4	Female	30.1%	Male	31.0%	0.867
Case 5	Feminine	32.2%	Masculine	29.2%	0.601

Panel D: Competitiveness in NEUTRAL compared to the other treatments

	NEUTRAL	FEMININE		MASCULINE	
Subject groups			p-value		p-value
Cismen	30.6%	39.4%	0.265	45.1%	0.073
Ciswomen	32.4%	27.1%	0.495	27.1%	0.495
Transmen	27.8%	26.4%	0.851	26.8%	0.891
Transwomen	31.8%	36.0%	0.669	26.1%	0.549

Note: The column p-value reports the results of χ^2 tests performed column-wise.

5.3 Regressions

Supplementary Table 15 Probit regression for NEUTRAL. Dependent variable: Competition.

	(1)	(2)	(3)	(4)	(5)
Ciswomen	0.052	0.173	0.281	0.360	0.268
	(0.220)	(0.231)	(0.254)	(0.297)	(0.254)
Transmen	-0.081	0.013	0.106	0.114	0.516
	(0.223)	(0.231)	(0.239)	(0.292)	(0.462)
Transwomen	0.036	0.108	0.084	-0.076	0.478
	(0.252)	(0.261)	(0.265)	(0.306)	(0.472)
Perf. tournament		0.003	-0.064 *	-0.054	-0.064 *
		(0.025)	(0.029)	(0.030)	(0.029)
Delta perf.		0.122 ***	0.096 *	0.103 **	0.095 *
		(0.035)	(0.039)	(0.038)	(0.040)
Belief tournament			-0.522 ***	-0.516 ***	-0.518 ***
			(0.123)	(0.127)	(0.124)
Risk			0.124	0.137	0.130
			(0.074)	(0.077)	(0.074)
Age				0.026 *	
				(0.012)	
Height				0.007	
				(0.010)	
Student status				-0.073	
				(0.209)	
Income: < 20,000 GBP				0.442	
				(0.232)	
Religion: Religious				0.081	
				(0.207)	
Religion: Not say				0.268	
				(0.485)	
Residence: US				0.159	
				(0.254)	
Residence: UK				0.421	
				(0.270)	
Residence: Other				0.564	
				(0.391)	
TCS					0.094
					(0.139)
STT					-0.033
					(0.033)
Const.	-0.508 **	-0.670 *	0.823	-1.618	0.419
	(0.156)	(0.272)	(0.468)	(2.000)	(0.789)
N	259	259	259	259	259
Pseudo R2 (McFadden)	0.001	0.059	0.129	0.168	0.132
H_0: Sex	0.846	0.823	0.415	0.214	0.420
H_0: Gender	0.614	0.437	0.470	0.656	0.527

Note: Competition is a binary variable equal to 1 if the participant enters the tournament in Part 4 and 0 otherwise. Delta perf. is the difference in performance between Part 3 (tournament) and Part 2 (piece-rate). Belief tournament is the participants' belief of their performance rank within their group in Part 3, where the value 1 represents the rank with the highest performance. Standard errors in parentheses are heteroskedasticity robust. In the second last column from the right, the baseline is a non-student, non-religious cisman, who earns more than 20K GBP, and lives in continental Europe. In the last column from the right, the baseline is a cisman. *** $p < 0.001$; ** $p < 0.01$; * $p < 0.05$. H_0: Sex reports the p-values of a joint coefficient test comparing Male (Cismen and Transwomen) with Female (Ciswomen and Transmen). H_0: Gender reports the p-values of a joint coefficient test comparing Masculine (Cismen and Transmen) with Feminine (Ciswomen and Transwomen).

Supplementary Table 16 Probit regression for all treatments. Dependent variable: Competition.

	(1)	(2)	(3)	(4)	(5)
Ciswomen	0.052	0.167	0.307	0.461	0.280
	(0.220)	(0.229)	(0.255)	(0.270)	(0.255)
Transmen	-0.081	0.008	0.127	0.189	0.634
	(0.223)	(0.230)	(0.241)	(0.270)	(0.343)
Transwomen	0.036	0.122	0.078	0.079	0.635
	(0.252)	(0.262)	(0.272)	(0.287)	(0.359)
Treatment: FEMININE	0.241	0.187	0.129	0.167	0.104
	(0.218)	(0.223)	(0.227)	(0.241)	(0.231)
Treatment: MASCULINE	0.385	0.473 *	0.428	0.544 *	0.423
	(0.217)	(0.225)	(0.232)	(0.246)	(0.234)
FEMININE x Ciswomen	-0.392	-0.339	-0.318	-0.383	-0.285
	(0.313)	(0.329)	(0.360)	(0.365)	(0.360)
MASCULINE x Ciswomen	-0.536	-0.678 *	-0.645	-0.755 *	-0.614
	(0.312)	(0.323)	(0.342)	(0.348)	(0.343)
FEMININE x Transmen	-0.282	-0.201	-0.107	-0.112	-0.052
	(0.313)	(0.325)	(0.329)	(0.340)	(0.333)
MASCULINE x Transmen	-0.415	-0.543	-0.405	-0.475	-0.369
	(0.313)	(0.319)	(0.327)	(0.341)	(0.333)
FEMININE x Transwomen	-0.126	-0.018	0.187	0.131	0.150
	(0.347)	(0.352)	(0.357)	(0.366)	(0.360)
MASCULINE x Transwomen	-0.552	-0.690	-0.573	-0.651	-0.583
	(0.356)	(0.366)	(0.377)	(0.393)	(0.379)
Perf. tournament		-0.008	-0.067 ***	-0.066 ***	-0.066 ***
		(0.014)	(0.016)	(0.016)	(0.016)
Delta perf.		0.136 ***	0.108 ***	0.114 ***	0.110 ***
		(0.020)	(0.021)	(0.022)	(0.021)
Belief tournament			-0.561 ***	-0.557 ***	-0.543 ***
			(0.071)	(0.072)	(0.071)
Risk			0.150 ***	0.153 ***	0.153 ***
			(0.045)	(0.046)	(0.045)
Const.	-0.508 **	-0.587 **	0.865 **	-1.757	-0.072
	(0.156)	(0.200)	(0.310)	(1.255)	(0.480)
Controls (Age, Height, Student status, Income, Religion, Residence)	–	–	–	Yes	–
Controls (TCS, STT)	–	–	–	–	Yes
N	780	780	780	780	780
Pseudo R2 (McFadden)	0.013	0.082	0.161	0.178	0.167
H_0: FEMININE on Cismen	0.266	0.402	0.579	0.478	0.656
H_0: MASCULINE on Cismen	0.074	0.034	0.063	0.021	0.068
H_0: FEMININE on Ciswomen	0.495	0.508	0.432	0.374	0.452
H_0: MASCULINE on Ciswomen	0.495	0.374	0.368	0.387	0.426
H_0: FEMININE on Transmen	0.851	0.953	0.928	0.821	0.832
H_0: MASCULINE on Transmen	0.891	0.761	0.922	0.779	0.828
H_0: FEMININE on Transwomen	0.669	0.541	0.274	0.310	0.386
H_0: MASCULINE on Transwomen	0.549	0.449	0.627	0.724	0.591

Note: Competition is a binary variable equal to 1 if the participant enters the tournament in Part 4 and 0 otherwise. Delta perf. is the difference in performance between Part 3 (tournament) and Part 2 (piece-rate). Belief tournament is the participants' belief of their performance rank within their group in Part 3, where the value 1 represents the rank with the highest performance. Standard errors in parentheses are heteroskedasticity robust. In the second last column from the right, the baseline is a non-student, non-religious cisman, who earns more than 20K GBP, and lives in continental Europe. In the last column from the right, the baseline is a cisman. *** $p < 0.001$; ** $p < 0.01$; * $p < 0.05$. Rows starting with H_0 report the p-values of a joint coefficient test that the coefficients' sum equals 0. For example, "H_0: FEMININE on Ciswomen" tests the effect of the treatment (FEMININE) on the subject group (Ciswomen).

5.4 Cohen's *d*

Supplementary Table 17 Cohen's d analysis: Competitiveness

	|Cohen's d|	Cohen's d	CI.95-Lower	CI.95-Upper	t-statistic	p-value
**Within NEUTRAL**						
Female vs. Male	0.021	-0.021	-0.266	0.224	-0.169	0.866
Feminine vs. Masculine	0.065	0.065	-0.180	0.310	0.526	0.599
Cismen vs. Ciswomen	0.040	-0.040	-0.367	0.288	-0.237	0.813
Cismen vs. Transmen	0.061	0.061	-0.266	0.388	0.367	0.714
Cismen vs. Transwomen	0.027	-0.027	-0.402	0.348	-0.147	0.883
Ciswomen vs. Transmen	0.101	0.101	-0.228	0.429	0.603	0.548
Ciswomen vs. Transwomen	0.012	0.012	-0.364	0.388	0.066	0.947
Transmen vs. Transwomen	0.089	-0.089	-0.464	0.287	-0.478	0.633
**Between treatments**						
NEUTRAL vs. FEMININE	0.031	-0.031	-0.203	0.141	-0.354	0.723
Cismen	0.187	-0.187	-0.516	0.143	-1.118	0.265
Ciswomen	0.115	0.115	-0.216	0.445	0.683	0.496
Transmen	0.031	0.031	-0.296	0.358	0.188	0.852
Transwomen	0.088	-0.088	-0.493	0.318	-0.428	0.669
NEUTRAL vs. MASCULINE	0.028	-0.028	-0.200	0.145	-0.315	0.753
Cismen	0.303	-0.303	-0.633	0.030	-1.811	0.072
Ciswomen	0.115	0.115	-0.216	0.445	0.683	0.496
Transmen	0.023	0.023	-0.305	0.351	0.137	0.892
Transwomen	0.127	0.127	-0.288	0.540	0.601	0.549

6 Payoffs (Part 4)

6.1 Regressions

Supplementary Table 18 OLS regression for NEUTRAL. Dependent variable: Payoff in Part 4.

	(1)	(2)	(3)
Ciswomen	-0.156	1.315	-0.267
	(1.183)	(1.326)	(1.177)
Transmen	0.951	2.316	3.377
	(1.256)	(1.358)	(2.170)
Transwomen	-1.071	-0.560	1.571
	(1.244)	(1.278)	(2.215)
Age		-0.040	
		(0.047)	
Height		0.113 **	
		(0.041)	
Student status		3.479 ***	
		(1.017)	
Income: < 20,000 GBP		0.402	
		(0.922)	
Religion: Religious		-2.336 *	
		(0.953)	
Religion: Not say		-3.673 **	
		(1.247)	
Residence: US		1.421	
		(1.212)	
Residence: UK		1.468	
		(1.176)	
Residence: Other		-1.269	
		(1.576)	
TCS			1.036
			(0.634)
STT			-0.099
			(0.153)
Const.	5.389 ***	-15.595 *	0.895
	(0.851)	(7.668)	(2.853)
N	259	259	259
Adj. R2	-0.003	0.073	-0.002

H_0: Sex	0.289	0.056	0.366
H_0: Gender	0.215	0.381	0.234

Note: Standard errors in parentheses are heteroskedasticity robust. In the second last column from the right, the baseline is a non-student, non-religious cisman, who earns more than 20K GBP, and lives in continental Europe. In the last column from the right, the baseline is a cisman. *** $p < 0.001$; ** $p < 0.01$; * $p < 0.05$. H_0: Sex reports the p-values of a joint coefficient test comparing Male (Cismen and Transwomen) with Female (Ciswomen and Transmen). H_0: Gender reports the p-values of a joint coefficient test comparing Masculine (Cismen and Transmen) with Feminine (Ciswomen and Transwomen).

Supplementary Table 19 OLS regression for all treatments. Dependent variable: Payoff in Part 4.

	(1)	(2)	(3)
Ciswomen	-0.156	0.922	-0.225
	(1.183)	(1.245)	(1.178)
Transmen	0.951	1.785	1.993
	(1.256)	(1.279)	(1.614)
Transwomen	-1.071	-0.976	0.193
	(1.244)	(1.250)	(1.643)
Treatment: FEMININE	2.660	2.622	2.632
	(1.516)	(1.483)	(1.525)
Treatment: MASCULINE	0.442	0.354	0.435
	(1.331)	(1.343)	(1.328)
FEMININE x Ciswomen	-2.293	-2.286	-2.207
	(1.925)	(1.892)	(1.931)
MASCULINE x Ciswomen	-1.096	-1.243	-1.003
	(1.700)	(1.717)	(1.692)
FEMININE x Transmen	-4.501 *	-4.672 *	-4.383 *
	(1.905)	(1.868)	(1.909)
MASCULINE x Transmen	-0.818	-0.744	-0.781
	(1.859)	(1.850)	(1.849)
FEMININE x Transwomen	-1.689	-1.536	-1.664
	(2.111)	(2.116)	(2.108)
MASCULINE x Transwomen	-0.075	-0.088	-0.054
	(1.813)	(1.770)	(1.827)
Const.	5.389 ***	-8.821	2.454
	(0.851)	(5.310)	(1.658)
Controls (Age, Height, Student status, Income, Religion, Residence)	–	Yes	–
Controls (TCS, STT)	–	–	Yes
N	780	780	780
Adj. R2	0.003	0.034	0.005

H_0: FEMININE on Cismen	0.080	0.077	0.085
H_0: MASCULINE on Cismen	0.740	0.792	0.743
H_0: FEMININE on Ciswomen	0.757	0.773	0.719
H_0: MASCULINE on Ciswomen	0.536	0.411	0.589
H_0: FEMININE on Transmen	0.111	0.070	0.129
H_0: MASCULINE on Transmen	0.772	0.760	0.788
H_0: FEMININE on Transwomen	0.509	0.472	0.514
H_0: MASCULINE on Transwomen	0.766	0.822	0.762

Note: Standard errors in parentheses are heteroskedasticity robust. In the second last column from the right, the baseline is a non-student, non-religious cisman, who earns more than 20K GBP, and lives in continental Europe. In the last column from the right, the baseline is a cisman. *** $p < 0.001$; ** $p < 0.01$; * $p < 0.05$. Rows starting with H_0 report the p-values of a joint coefficient test that the coefficients' sum equals 0. For example, "H_0: FEMININE on Ciswomen" tests the effect of the treatment (FEMININE) on the subject group (Ciswomen).

7 Risk (Part 5)

7.1 Bar graphs

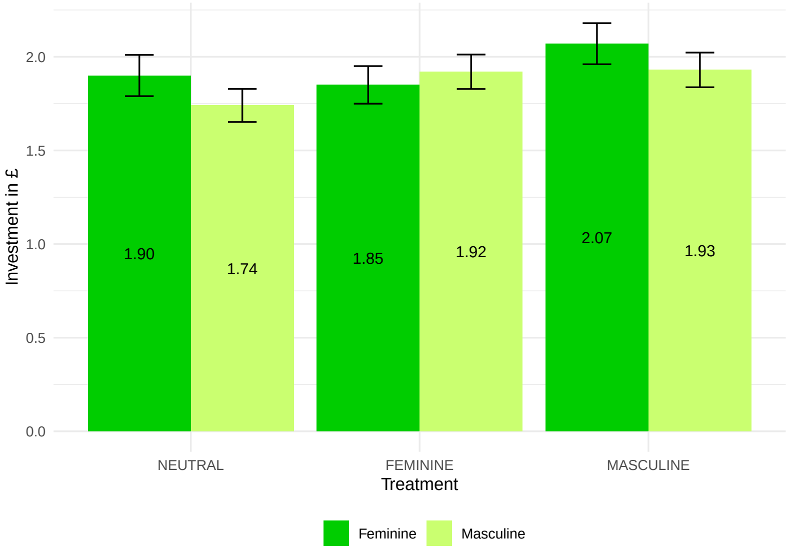

Supplementary Figure 4 Investment into the risky lottery in Part 5 by treatments and gender ($n = 780$). The bars show the average investment rate, and the error bars represent the standard error of the mean.

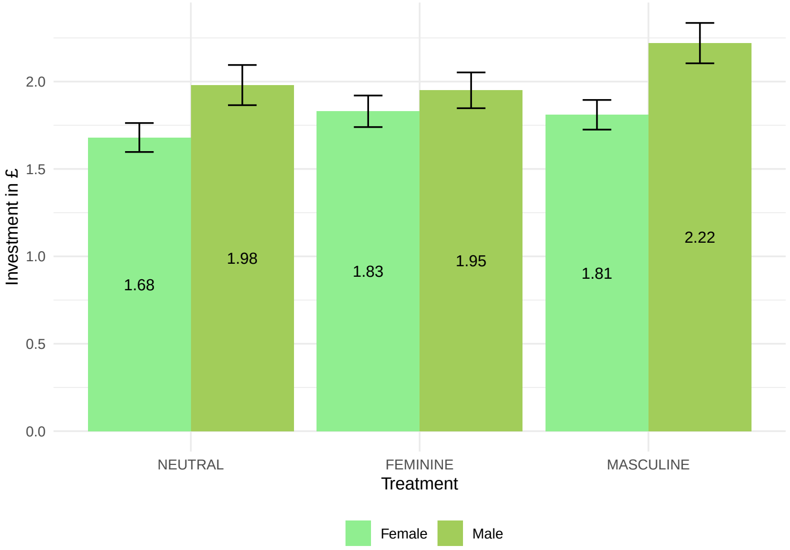

Supplementary Figure 5 Investment into the risky lottery in Part 5 by treatments and sex ($n = 780$). The bars show the average investment rate, and the error bars represent the standard error of the mean.

7.2 Non-parametric tests

Supplementary Table 20 Investment into the risky lottery across treatments and subject groups.

Panel A: Risk across treatments

	Treatment			
Subject groups	NEUTRAL	FEMININE	MASCULINE	p-value
Cismen	1.814	2.021	2.208	0.119
Ciswomen	1.690	1.852	1.972	0.446
Transmen	1.673	1.816	1.655	0.660
Transwomen	2.244	1.840	2.227	0.357

Panel B: Risk across subject groups

	Subject groups				
Treatment	Cismen	Ciswomen	Transmen	Transwomen	p-value
NEUTRAL	1.814	1.690	1.673	2.244	0.194
FEMININE	2.021	1.852	1.816	1.840	0.715
MASCULINE	2.208	1.972	1.655	2.227	0.030

Panel C: Risk across groups within NEUTRAL

	Group 1		Group 2		
	Subjects		Subjects		p-value
Case 1	Cisgender	1.753	Transgender	1.890	0.461
Case 2	Cismen	1.814	Ciswomen	1.690	0.704
Case 3	Transmen	1.673	Transwomen	2.244	0.048
Case 4	Female	1.681	Male	1.977	0.130
Case 5	Feminine	1.902	Masculine	1.743	0.355

Panel D: Risk in NEUTRAL compared to the other treatments

	NEUTRAL	FEMININE		MASCULINE	
Subject groups			p-value		p-value
Cismen	1.814	2.021	0.262	2.208	0.038
Ciswomen	1.690	1.852	0.550	1.972	0.208
Transmen	1.673	1.816	0.479	1.655	0.881
Transwomen	2.244	1.840	0.206	2.227	0.927

Note: The column p-value reports the results of the tests performed column-wise. For continuous variables, we conducted Mann-Whitney U tests for two groups and Kruskal-Wallis tests for more than two groups.

7.3 Regressions

Supplementary Table 21 OLS regression for NEUTRAL. Dependent variable: Risk.

	(1)	(2)	(3)
Ciswomen	-0.124	-0.164	-0.109
	(0.179)	(0.218)	(0.177)
Transmen	-0.141	-0.221	-0.295
	(0.177)	(0.212)	(0.396)
Transwomen	0.430	0.316	0.216
	(0.245)	(0.258)	(0.412)
Age		-0.008	
		(0.010)	
Height		-0.001	
		(0.008)	
Student status		0.064	
		(0.161)	
Income: < 20,000 GBP		-0.125	
		(0.162)	
Religion: Religious		-0.045	
		(0.160)	
Religion: Not say		-0.502	
		(0.414)	
Residence: US		0.052	
		(0.202)	
Residence: UK		0.145	
		(0.194)	
Residence: Other		0.618 *	
		(0.309)	
TCS			-0.143
			(0.105)
STT			-0.009
			(0.029)
Const.	1.814 ***	2.273	2.463 ***
	(0.133)	(1.565)	(0.499)
N	259	259	259
Adj. R2	0.022	0.018	0.024
H_0: Sex	0.020	0.042	0.037
H_0: Gender	0.132	0.228	0.183

Note: Standard errors in parentheses are heteroskedasticity robust. In the second last column from the right, the baseline is a non-student, non-religious cisman, who earns more than 20K GBP, and lives in continental Europe. In the last column from the right, the baseline is a cisman. *** $p < 0.001$; ** $p < 0.01$; * $p < 0.05$. H_0: Sex reports the p-values of a joint coefficient test comparing Male (Cismen and Transwomen) with Female (Ciswomen and Transmen). H_0: Gender reports the p-values of a joint coefficient test comparing Masculine (Cismen and Transmen) with Feminine (Ciswomen and Transwomen).

Supplementary Table 22 OLS regression for all treatments. Dependent variable: Risk.

	(1)	(2)	(3)
Ciswomen	-0.124	-0.123	-0.124
	(0.179)	(0.195)	(0.179)
Transmen	-0.141	-0.154	-0.273
	(0.177)	(0.189)	(0.286)
Transwomen	0.430	0.387	0.325
	(0.245)	(0.248)	(0.324)
Treatment: FEMININE	0.207	0.210	0.218
	(0.192)	(0.193)	(0.192)
Treatment: MASCULINE	0.394 *	0.386 *	0.393 *
	(0.191)	(0.193)	(0.191)
FEMININE x Ciswomen	-0.045	-0.048	-0.052
	(0.263)	(0.263)	(0.263)
MASCULINE x Ciswomen	-0.112	-0.118	-0.106
	(0.256)	(0.259)	(0.255)
FEMININE x Transmen	-0.064	-0.074	-0.073
	(0.255)	(0.257)	(0.255)
MASCULINE x Transmen	-0.412	-0.409	-0.410
	(0.252)	(0.253)	(0.252)
FEMININE x Transwomen	-0.611	-0.593	-0.585
	(0.319)	(0.320)	(0.322)
MASCULINE x Transwomen	-0.412	-0.380	-0.403
	(0.348)	(0.350)	(0.349)
Const.	1.814 ***	1.605	1.762 ***
	(0.133)	(0.913)	(0.308)
Controls (Age, Height, Student status, Income, Religion, Residence)	–	Yes	–
Controls (TCS, STT)	–	–	Yes
N	780	780	780
Adj. R2	0.018	0.013	0.017

H_0: FEMININE on Cismen	0.282	0.278	0.257
H_0: MASCULINE on Cismen	0.039	0.046	0.040
H_0: FEMININE on Ciswomen	0.368	0.370	0.356
H_0: MASCULINE on Ciswomen	0.097	0.125	0.092
H_0: FEMININE on Transmen	0.395	0.426	0.388
H_0: MASCULINE on Transmen	0.915	0.889	0.918
H_0: FEMININE on Transwomen	0.113	0.132	0.156
H_0: MASCULINE on Transwomen	0.951	0.984	0.975

Note: Standard errors in parentheses are heteroskedasticity robust. In the second last column from the right, the baseline is a non-student, non-religious cisman, who earns more than 20K GBP, and lives in continental Europe. In the last column from the right, the baseline is a cisman. *** $p < 0.001$; ** $p < 0.01$; * $p < 0.05$. Rows starting with H_0 report the p-values of a joint coefficient test that the coefficients' sum equals 0. For example, "H_0: FEMININE on Ciswomen" tests the effect of the treatment (FEMININE) on the subject group (Ciswomen).

7.4 Cohen's *d*

Supplementary Table 23 Cohen's d analysis: Risk

	|Cohen's d|	Cohen's d	CI.95-Lower	CI.95-Upper	t-statistic	p-value
**Within NEUTRAL**						
Female vs. Male	0.268	-0.268	-0.515	-0.020	-2.156	0.032
Feminine vs. Masculine	0.143	0.143	-0.103	0.388	1.150	0.251
Cismen vs. Ciswomen	0.117	0.117	-0.212	0.445	0.698	0.486
Cismen vs. Transmen	0.134	0.134	-0.194	0.461	0.804	0.423
Cismen vs. Transwomen	0.354	-0.354	-0.735	0.030	-1.908	0.059
Ciswomen vs. Transmen	0.017	0.017	-0.310	0.345	0.104	0.917
Ciswomen vs. Transwomen	0.484	-0.484	-0.871	-0.092	-2.596	0.011
Transmen vs. Transwomen	0.504	-0.504	-0.891	-0.111	-2.712	0.008
**Between treatments**						
NEUTRAL vs. FEMININE	0.065	-0.065	-0.236	0.107	-0.740	0.459
Cismen	0.182	-0.182	-0.510	0.148	-1.086	0.279
Ciswomen	0.153	-0.153	-0.484	0.179	-0.909	0.365
Transmen	0.143	-0.143	-0.470	0.185	-0.857	0.393
Transwomen	0.335	0.335	-0.077	0.744	1.626	0.107
NEUTRAL vs. MASCULINE	0.161	-0.161	-0.334	0.012	-1.833	0.067
Cismen	0.348	-0.348	-0.680	-0.014	-2.080	0.039
Ciswomen	0.282	-0.282	-0.614	0.053	-1.673	0.097
Transmen	0.018	0.018	-0.310	0.346	0.107	0.915
Transwomen	0.013	0.013	-0.400	0.426	0.062	0.951

8 Altruism (Part 6)

8.1 Bar graphs

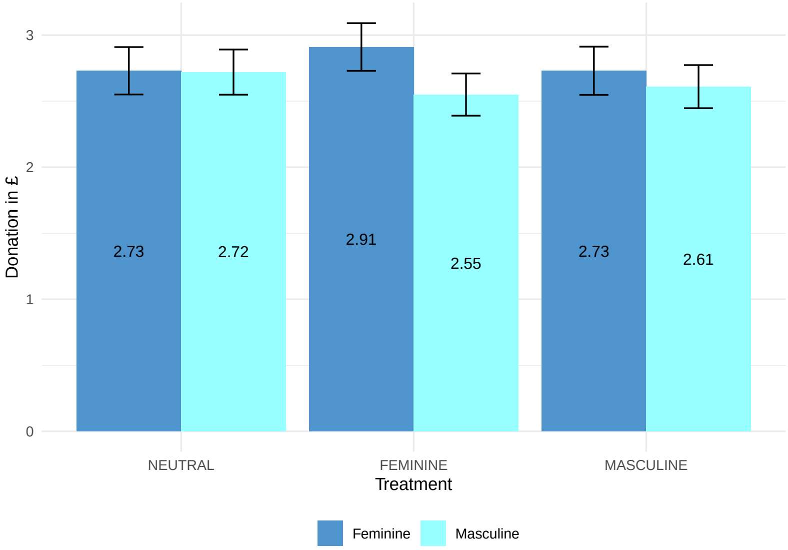

Supplementary Figure 6 Donation in Part 6 by treatments and gender ($n = 780$). The average donations are indicated by the bars and the error bars represent the standard error of the mean.

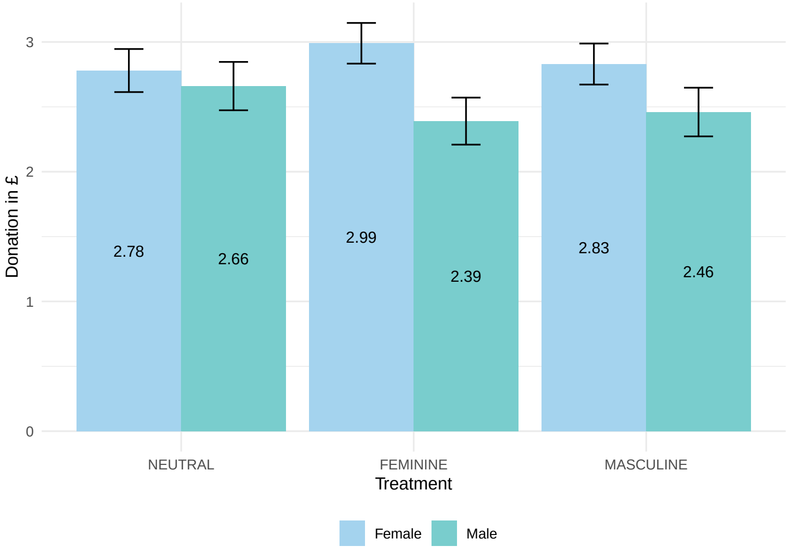

Supplementary Figure 7 Donation in Part 6 by treatments and sex ($n = 780$). The average donations are indicated by the bars and the error bars represent the standard error of the mean.

8.2 Non-parametric tests

Supplementary Table 24 Donations across treatments and subject groups.

Panel A: Donations across treatments

	Treatment			
Subject groups	NEUTRAL	FEMININE	MASCULINE	p-value
Cismen	2.685	2.265	2.423	0.582
Ciswomen	2.803	3.161	2.864	0.478
Transmen	2.762	2.822	2.792	0.999
Transwomen	2.615	2.556	2.525	0.999

Panel B: Donations across subject groups

	Subject groups				
Treatment	Cismen	Ciswomen	Transmen	Transwomen	p-value
NEUTRAL	2.685	2.803	2.762	2.615	0.933
FEMININE	2.265	3.161	2.822	2.556	0.073
MASCULINE	2.423	2.864	2.792	2.525	0.540

Panel C: Donations across groups within NEUTRAL

	Group 1		Group 2		
	Subjects		Subjects		p-value
Case 1	Cisgender	2.743	Transgender	2.706	0.871
Case 2	Cismen	2.685	Ciswomen	2.803	0.759
Case 3	Transmen	2.762	Transwomen	2.615	0.583
Case 4	Female	2.782	Male	2.658	0.564
Case 5	Feminine	2.731	Masculine	2.723	0.914

Panel D: Donations in NEUTRAL compared to the other treatments

	NEUTRAL	FEMININE		MASCULINE	
Subject groups			p-value		p-value
Cismen	2.685	2.265	0.322	2.423	0.454
Ciswomen	2.803	3.161	0.260	2.864	0.863
Transmen	2.762	2.822	0.982	2.792	0.977
Transwomen	2.615	2.556	0.948	2.525	0.987

Note: The columns p-value report the results of the tests performed column-wise. For continuous variables, we conducted Mann-Whitney U tests for two groups and Kruskal-Wallis tests for more than two groups.

8.3 Regressions

Supplementary Table 25 OLS regression for NEUTRAL. Dependent variable: Donations.

	(1)	(2)	(3)
Ciswomen	0.118	0.188	0.128
	(0.335)	(0.403)	(0.337)
Transmen	0.077	0.239	-0.162
	(0.343)	(0.387)	(0.646)
Transwomen	-0.070	0.103	-0.324
	(0.378)	(0.409)	(0.647)
Age		0.040 *	
		(0.016)	
Height		0.002	
		(0.013)	
Student status		0.713 **	
		(0.274)	
Income: < 20,000 GBP		0.025	
		(0.279)	
Religion: Religious		0.800 **	
		(0.274)	
Religion: Not say		0.701	
		(0.736)	
Residence: US		-0.789 *	
		(0.324)	
Residence: UK		-0.470	
		(0.335)	
Residence: Other		-0.391	
		(0.538)	
TCS			-0.091
			(0.199)
STT			0.012
			(0.046)
Const.	2.685 ***	1.123	3.077 ***
	(0.244)	(2.413)	(0.909)
N	259	259	259
Adj. R2	-0.011	0.044	-0.018

H_0: Sex	0.600	0.600	0.580
H_0: Gender	0.955	0.920	0.948

Note: Standard errors in parentheses are heteroskedasticity robust. In the second last column from the right, the baseline is a non-student, non-religious cisman, who earns more than 20K GBP, and lives in continental Europe. In the last column from the right, the baseline is a cisman. *** $p < 0.001$; ** $p < 0.01$; * $p < 0.05$. H_0: Sex reports the p-values of a joint coefficient test comparing Male (Cismen and Transwomen) with Female (Ciswomen and Transmen). H_0: Gender reports the p-values of a joint coefficient test comparing Masculine (Cismen and Transmen) with Feminine (Ciswomen and Transwomen).

Supplementary Table 26 OLS regression for all treatments. Dependent variable: Donations.

	(1)	(2)	(3)
Ciswomen	0.118	0.175	0.128
	(0.335)	(0.358)	(0.336)
Transmen	0.077	0.208	-0.036
	(0.343)	(0.351)	(0.465)
Transwomen	-0.070	-0.004	-0.222
	(0.378)	(0.386)	(0.477)
Treatment: FEMININE	-0.420	-0.377	-0.419
	(0.338)	(0.343)	(0.338)
Treatment: MASCULINE	-0.262	-0.229	-0.261
	(0.340)	(0.346)	(0.340)
FEMININE x Ciswomen	0.778	0.745	0.767
	(0.468)	(0.471)	(0.469)
MASCULINE x Ciswomen	0.323	0.355	0.308
	(0.468)	(0.470)	(0.468)
FEMININE x Transmen	0.480	0.410	0.465
	(0.468)	(0.458)	(0.468)
MASCULINE x Transmen	0.292	0.270	0.287
	(0.474)	(0.466)	(0.474)
FEMININE x Transwomen	0.361	0.444	0.350
	(0.530)	(0.533)	(0.529)
MASCULINE x Transwomen	0.172	0.191	0.166
	(0.543)	(0.547)	(0.545)
Const.	2.685 ***	1.806	3.117 ***
	(0.244)	(1.435)	(0.516)
Controls (Age, Height, Student status, Income, Religion, Residence)	–	Yes	–
Controls (TCS, STT)	–	–	Yes
N	780	780	780
Adj. R2	-0.000	0.023	-0.002
H_0: FEMININE on Cismen	0.214	0.272	0.216
H_0: MASCULINE on Cismen	0.441	0.509	0.444
H_0: FEMININE on Ciswomen	0.270	0.256	0.282
H_0: MASCULINE on Ciswomen	0.848	0.693	0.882
H_0: FEMININE on Transmen	0.854	0.914	0.886
H_0: MASCULINE on Transmen	0.926	0.895	0.937
H_0: FEMININE on Transwomen	0.885	0.870	0.867
H_0: MASCULINE on Transwomen	0.832	0.930	0.825

Note: Standard errors in parentheses are heteroskedasticity robust. In the second last column from the right, the baseline is a non-student, non-religious cisman, who earns more than 20K GBP, and lives in continental Europe. In the last column from the right, the baseline is a cisman. *** $p < 0.001$; ** $p < 0.01$; * $p < 0.05$. Rows starting with H_0 report the p-values of a joint coefficient test that the coefficients' sum equals 0. For example, "H_0: FEMININE on Ciswomen" tests the effect of the treatment (FEMININE) on the subject group (Ciswomen).

8.4 Cohen's *d*

Supplementary Table 27 Cohen's d analysis: Altruism

	|Cohen's d|	Cohen's d	CI.95-Lower	CI.95-Upper	t-statistic	p-value
**Within NEUTRAL**						
Female vs. Male	0.062	0.062	-0.183	0.307	0.502	0.616
Feminine vs. Masculine	0.004	0.004	-0.241	0.249	0.031	0.975
Cismen vs. Ciswomen	0.059	-0.059	-0.387	0.269	-0.355	0.723
Cismen vs. Transmen	0.038	-0.038	-0.364	0.289	-0.226	0.821
Cismen vs. Transwomen	0.035	0.035	-0.340	0.410	0.188	0.851
Ciswomen vs. Transmen	0.021	0.021	-0.307	0.348	0.124	0.902
Ciswomen vs. Transwomen	0.098	0.098	-0.279	0.474	0.526	0.600
Transmen vs. Transwomen	0.074	0.074	-0.302	0.449	0.399	0.691
**Between treatments**						
NEUTRAL vs. FEMININE	0.008	0.008	-0.164	0.180	0.091	0.928
Cismen	0.210	0.210	-0.121	0.538	1.253	0.212
Ciswomen	0.188	-0.188	-0.518	0.145	-1.113	0.267
Transmen	0.031	-0.031	-0.358	0.296	-0.186	0.853
Transwomen	0.030	0.030	-0.375	0.435	0.145	0.885
NEUTRAL vs. MASCULINE	0.033	0.033	-0.140	0.205	0.371	0.711
Cismen	0.130	0.130	-0.199	0.458	0.776	0.439
Ciswomen	0.032	-0.032	-0.363	0.298	-0.193	0.847
Transmen	0.016	-0.016	-0.343	0.312	-0.093	0.926
Transwomen	0.045	0.045	-0.369	0.458	0.214	0.831

9 Continuous gender measure (BEM)

9.1 Competitiveness

Supplementary Table 28 Probit regression for NEUTRAL. Dependent variable: Competition. Gender is measured on a continuous scale.

	(1)	(2)	(3)	(4)	(5)
BEM score: Feminine	0.010	0.014	0.016	0.017	0.016
	(0.011)	(0.011)	(0.012)	(0.013)	(0.012)
BEM score: Masculine	0.022	0.019	0.015	0.016	0.015
	(0.012)	(0.012)	(0.012)	(0.013)	(0.013)
Perf. tournament		0.004	-0.064 *	-0.057	-0.063 *
		(0.024)	(0.028)	(0.029)	(0.029)
Delta perf.		0.121 ***	0.097 *	0.108 **	0.097 *
		(0.034)	(0.038)	(0.038)	(0.039)
Belief tournament			-0.500 ***	-0.484 ***	-0.501 ***
			(0.123)	(0.126)	(0.122)
Risk			0.129	0.131	0.129
			(0.077)	(0.079)	(0.077)
Age				0.023	
				(0.012)	
Height				-0.000	
				(0.009)	
Student status				-0.114	
				(0.204)	
Income: < 20,000 GBP				0.464 *	
				(0.231)	
Religion: Religious				-0.001	
				(0.212)	
Religion: Not say				0.227	
				(0.471)	
Residence: US				0.094	
				(0.255)	
Residence: UK				0.391	
				(0.260)	
Residence: Other				0.495	
				(0.380)	
TCS					-0.012
					(0.096)
STT					-0.011
					(0.022)
Const.	-1.677 **	-1.847 **	-0.315	-1.493	-0.224
	(0.585)	(0.651)	(0.803)	(1.759)	(0.889)
N	259	259	259	259	259
Pseudo R2 (McFadden)	0.018	0.074	0.139	0.175	0.140

Note: Competition is a binary variable equal to 1 if the participant enters the tournament in Part 4 and 0 otherwise. Delta perf. is the difference in performance between Part 3 (tournament) and Part 2 (piece-rate). Belief tournament is the participants' belief of their performance rank within their group in Part 3, where the value 1 represents the rank with the highest performance. Standard errors in parentheses are heteroskedasticity robust. In the second last column from the right, the baseline is a non-student, non-religious person, who earns more than 20K GBP, and lives in continental Europe. *** $p < 0.001$; ** $p < 0.01$; * $p < 0.05$.

Supplementary Table 29 Probit regression for all treatments. Dependent variable: Competition. Gender is measured on a continuous scale.

	(1)	(2)	(3)	(4)	(5)
BEM score: Feminine	0.010	0.014	0.017	0.013	0.017
	(0.011)	(0.011)	(0.012)	(0.012)	(0.012)
BEM score: Masculine	0.022	0.018	0.014	0.014	0.012
	(0.012)	(0.012)	(0.013)	(0.013)	(0.013)
FEMININE x BEM score: Masculine	0.002	0.003	-0.001	0.000	-0.001
	(0.016)	(0.016)	(0.017)	(0.017)	(0.017)
MASCULINE x BEM score: Masculine	0.001	0.004	0.003	0.007	0.004
	(0.016)	(0.016)	(0.017)	(0.017)	(0.017)
FEMININE x BEM score: Feminine	-0.017	-0.018	-0.026	-0.022	-0.027
	(0.014)	(0.014)	(0.015)	(0.016)	(0.016)
MASCULINE x BEM score: Feminine	-0.015	-0.021	-0.029	-0.029	-0.031
	(0.015)	(0.015)	(0.016)	(0.016)	(0.016)
Treatment: FEMININE	0.652	0.706	1.148	0.972	1.219
	(0.729)	(0.750)	(0.797)	(0.832)	(0.820)
Treatment: MASCULINE	0.671	0.771	1.166	1.061	1.210
	(0.783)	(0.810)	(0.884)	(0.913)	(0.895)
Perf. tournament		-0.003	-0.067 ***	-0.065 ***	-0.068 ***
		(0.013)	(0.016)	(0.016)	(0.017)
Delta perf.		0.132 ***	0.106 ***	0.112 ***	0.107 ***
		(0.019)	(0.021)	(0.022)	(0.021)
Belief tournament			-0.557 ***	-0.552 ***	-0.551 ***
			(0.071)	(0.071)	(0.071)
Risk			0.151 ***	0.152 ***	0.153 ***
			(0.045)	(0.046)	(0.045)
Const.	-1.677 **	-1.794 **	-0.241	-1.998	-0.521
	(0.585)	(0.628)	(0.719)	(1.227)	(0.767)
Controls (Age, Height, Student status, Income, Religion, Residence)	–	–	–	Yes	–
Controls (TCS, STT)	–	–	–	–	Yes
N	780	780	780	780	780
Pseudo R2 (McFadden)	0.016	0.085	0.163	0.179	0.166
H_0: FEMININE on BEM score: Feminine	0.361	0.338	0.135	0.213	0.115
H_0: MASCULINE on BEM score: Feminine	0.381	0.329	0.160	0.206	0.145
H_0: FEMININE on BEM score: Masculine	0.347	0.324	0.127	0.203	0.108
H_0: MASCULINE on BEM score: Masculine	0.370	0.315	0.149	0.191	0.135

Note: Competition is a binary variable equal to 1 if the participant enters the tournament in Part 4 and 0 otherwise. Delta perf. is the difference in performance between Part 3 (tournament) and Part 2 (piece-rate). Belief tournament is the participants' belief of their performance rank within their group in Part 3, where the value 1 represents the rank with the highest performance. Standard errors in parentheses are heteroskedasticity robust. In the second last column from the right, the baseline is a non-student, non-religious person, who earns more than 20K GBP, and lives in continental Europe. *** $p < 0.001$; ** $p < 0.01$; * $p < 0.05$. Rows starting with H_0 report the p-values of a joint coefficient test that the coefficients' sum equals 0. For example, "H_0: FEMININE on BEM score: Feminine" tests the effect of the treatment (FEMININE) on the subject group (BEM score: Feminine).

9.2 Risk

Supplementary Table 30 OLS regression for NEUTRAL. Dependent variable: Risk. Gender is measured on a continuous scale.

	(1)	(2)	(3)
BEM score: Feminine	-0.009	-0.008	-0.009
	(0.009)	(0.009)	(0.009)
BEM score: Masculine	0.006	0.006	0.010
	(0.009)	(0.010)	(0.009)
Age		-0.008	
		(0.009)	
Height		0.005	
		(0.007)	
Student status		0.045	
		(0.160)	
Income: < 20,000 GBP		-0.091	
		(0.161)	
Religion: Religious		-0.067	
		(0.167)	
Religion: Not say		-0.518	
		(0.419)	
Residence: US		0.091	
		(0.204)	
Residence: UK		0.167	
		(0.190)	
Residence: Other		0.769 *	
		(0.302)	
TCS			-0.173 *
			(0.078)
STT			-0.019
			(0.018)
Const.	2.011 ***	1.262	2.577 ***
	(0.440)	(1.339)	(0.568)
N	259	259	259
Adj. R2	-0.002	0.004	0.012

Note: Standard errors in parentheses are heteroskedasticity robust. In the second last column from the right, the baseline is a non-student, non-religious person, who earns more than 20K GBP, and lives in continental Europe. *** $p < 0.001$; ** $p < 0.01$; * $p < 0.05$.

Supplementary Table 31 OLS regression for all treatments. Dependent variable: Risk. Gender is measured on a continuous scale.

	(1)	(2)	(3)
BEM score: Feminine	-0.009	-0.009	-0.010
	(0.009)	(0.009)	(0.009)
BEM score: Masculine	0.006	0.005	0.005
	(0.009)	(0.009)	(0.009)
FEMININE x BEM score: Masculine	-0.000	-0.000	-0.001
	(0.013)	(0.013)	(0.013)
MASCULINE x BEM score: Masculine	-0.001	-0.000	-0.001
	(0.014)	(0.014)	(0.014)
FEMININE x BEM score: Feminine	0.001	0.001	0.001
	(0.012)	(0.012)	(0.012)
MASCULINE x BEM score: Feminine	0.017	0.016	0.017
	(0.012)	(0.012)	(0.013)
Treatment: FEMININE	0.045	0.044	0.061
	(0.596)	(0.600)	(0.602)
Treatment: MASCULINE	-0.489	-0.503	-0.485
	(0.635)	(0.640)	(0.637)
Const.	2.011 ***	0.739	1.953 ***
	(0.440)	(0.861)	(0.482)
Controls (Age, Height, Student status, Income, Religion, Residence)	–	Yes	–
Controls (TCS, STT)	–	–	Yes
N	780	780	780
Adj. R2	-0.001	-0.001	-0.003
H_0: FEMININE on BEM score: Feminine	0.938	0.939	0.917
H_0: MASCULINE on BEM score: Feminine	0.452	0.441	0.457
H_0: FEMININE on BEM score: Masculine	0.940	0.941	0.919
H_0: MASCULINE on BEM score: Masculine	0.435	0.426	0.440

Note: Standard errors in parentheses are heteroskedasticity robust. In the second last column from the right, the baseline is a non-student, non-religious person, who earns more than 20K GBP, and lives in continental Europe. *** $p < 0.001$; ** $p < 0.01$; * $p < 0.05$. Rows starting with H_0 report the p-values of a joint coefficient test that the coefficients' sum equals 0. For example, "H_0: FEMININE on BEM score: Feminine" tests the effect of the treatment (FEMININE) on the subject group (BEM score: Feminine).

9.3 Altruism

Supplementary Table 32 OLS regression for NEUTRAL. Dependent variable: Donations. Gender is measured on a continuous scale.

	(1)	(2)	(3)
BEM score: Feminine	0.023	0.018	0.023
	(0.015)	(0.015)	(0.016)
BEM score: Masculine	0.002	-0.014	0.003
	(0.016)	(0.016)	(0.016)
Age		0.040 *	
		(0.017)	
Height		-0.002	
		(0.010)	
Student status		0.710 *	
		(0.278)	
Income: < 20,000 GBP		0.051	
		(0.275)	
Religion: Religious		0.797 **	
		(0.279)	
Religion: Not say		0.608	
		(0.722)	
Residence: US		-0.831 **	
		(0.309)	
Residence: UK		-0.480	
		(0.330)	
Residence: Other		-0.401	
		(0.499)	
TCS			-0.031
			(0.133)
STT			-0.002
			(0.031)
Const.	1.713 *	1.573	1.808
	(0.774)	(1.963)	(0.919)
N	259	259	259
Adj. R2	0.001	0.054	-0.007

Note: Standard errors in parentheses are heteroskedasticity robust. In the second last column from the right, the baseline is a non-student, non-religious person, who earns more than 20K GBP, and lives in continental Europe. *** $p < 0.001$; ** $p < 0.01$; * $p < 0.05$.

Supplementary Table 33 OLS regression for all treatments. Dependent variable: Donations. Gender is measured on a continuous scale.

	(1)	(2)	(3)
BEM score: Feminine	0.023	0.022	0.023
	(0.015)	(0.015)	(0.015)
BEM score: Masculine	0.002	-0.006	0.004
	(0.016)	(0.016)	(0.016)
FEMININE x BEM score: Masculine	-0.003	0.003	-0.002
	(0.023)	(0.023)	(0.023)
MASCULINE x BEM score: Masculine	0.002	0.002	0.001
	(0.022)	(0.022)	(0.022)
FEMININE x BEM score: Feminine	0.013	0.009	0.015
	(0.020)	(0.020)	(0.020)
MASCULINE x BEM score: Feminine	0.019	0.018	0.021
	(0.021)	(0.020)	(0.021)
Treatment: FEMININE	-0.484	-0.446	-0.603
	(1.012)	(1.007)	(1.012)
Treatment: MASCULINE	-0.927	-0.841	-0.976
	(1.067)	(1.052)	(1.076)
Const.	1.713 *	2.827 *	2.100 *
	(0.774)	(1.351)	(0.821)
Controls (Age, Height, Student status, Income, Religion, Residence)	–	Yes	–
Controls (TCS, STT)	–	–	Yes
N	780	780	780
Adj. R2	0.013	0.034	0.014
H_0: FEMININE on BEM score: Feminine	0.638	0.660	0.556
H_0: MASCULINE on BEM score: Feminine	0.389	0.428	0.368
H_0: FEMININE on BEM score: Masculine	0.627	0.656	0.545
H_0: MASCULINE on BEM score: Masculine	0.381	0.420	0.359

Note: Standard errors in parentheses are heteroskedasticity robust. In the second last column from the right, the baseline is a non-student, non-religious person, who earns more than 20K GBP, and lives in continental Europe. *** $p < 0.001$; ** $p < 0.01$; * $p < 0.05$. Rows starting with H_0 report the p-values of a joint coefficient test that the coefficients' sum equals 0. For example, "H_0: FEMININE on BEM score: Feminine" tests the effect of the treatment (FEMININE) on the subject group (BEM score: Feminine).

10 Controlling for the strength of the primining intervention

10.1 Competitiveness

Supplementary Table 34 Probit regression for all treatments. Dependent variable: Competition.

	(1)	(2)	(3)	(4)	(5)
Ciswomen	-0.080	0.015	0.177	0.305	0.167
	(0.190)	(0.201)	(0.221)	(0.237)	(0.222)
Transmen	-0.094	-0.021	0.126	0.170	0.616 *
	(0.188)	(0.197)	(0.201)	(0.229)	(0.313)
Transwomen	-0.140	-0.051	-0.021	-0.020	0.509
	(0.216)	(0.224)	(0.233)	(0.243)	(0.324)
Rem. feminine words	0.028	0.009	0.009	0.018	0.006
	(0.039)	(0.038)	(0.037)	(0.039)	(0.038)
Rem. masculine words	0.042	0.054	0.047	0.070	0.048
	(0.044)	(0.045)	(0.046)	(0.048)	(0.046)
Rem. feminine words x Ciswomen	-0.058	-0.038	-0.042	-0.055	-0.040
	(0.053)	(0.054)	(0.057)	(0.058)	(0.057)
Rem. masculine words x Ciswomen	-0.063	-0.088	-0.087	-0.108	-0.086
	(0.061)	(0.062)	(0.063)	(0.064)	(0.062)
Rem. feminine words x Transmen	-0.063	-0.043	-0.036	-0.039	-0.024
	(0.053)	(0.052)	(0.052)	(0.053)	(0.052)
Rem. masculine words x Transmen	-0.085	-0.106	-0.082	-0.095	-0.072
	(0.058)	(0.058)	(0.061)	(0.065)	(0.061)
Rem. feminine words x Transwomen	0.021	0.038	0.056	0.041	0.056
	(0.060)	(0.059)	(0.059)	(0.059)	(0.059)
Rem. masculine words x Transwomen	-0.068	-0.092	-0.091	-0.109	-0.091
	(0.068)	(0.069)	(0.074)	(0.075)	(0.072)
Const.	-0.394 **	-0.463 *	0.954 **	-1.462	0.014
	(0.131)	(0.183)	(0.293)	(1.248)	(0.468)
Controls (Age, Height, Student status, Income, Religion, Residence)	–	–	–	Yes	–
Controls (TCS, STT)	–	–	–	–	Yes
N	780	780	780	780	780
Pseudo R2 (McFadden)	0.013	0.081	0.160	0.176	0.167
H_0: Rem. feminine words on Cismen	0.472	0.819	0.830	0.663	0.880
H_0: Rem. masculine words on Cismen	0.337	0.242	0.317	0.145	0.312
H_0: Rem. feminine words on Ciswomen	0.415	0.446	0.402	0.364	0.395
H_0: Rem. masculine words on Ciswomen	0.600	0.402	0.352	0.388	0.373
H_0: Rem. feminine words on Transmen	0.322	0.349	0.476	0.588	0.638
H_0: Rem. masculine words on Transmen	0.273	0.198	0.415	0.571	0.587
H_0: Rem. feminine words on Transwomen	0.279	0.315	0.177	0.223	0.203
H_0: Rem. masculine words on Transwomen	0.613	0.462	0.420	0.469	0.429

Note: Competition is a binary variable equal to 1 if the participant enters the tournament in Part 4 and 0 otherwise. Delta perf. is the difference in performance between Part 3 (tournament) and Part 2 (piece-rate). In the second last column from the right, the baseline is a non-student, non-religious cisman, who earns more than 20K GBP, and lives in continental Europe. In the last column from the right, the baseline is a cisman. *** $p < 0.001$; ** $p < 0.01$; * $p < 0.05$. Rows starting with H_0 report the p-values of a joint coefficient test that the coefficients' sum equals 0. For example, "H_0: Rem. feminine words on Ciswomen" tests the effect of Rem. feminine words on the subject group (Ciswomen).

10.2 Risk

Supplementary Table 35 OLS regression for all treatments. Dependent variable: Risk.

	(1)	(2)	(3)
Ciswomen	-0.063	-0.052	-0.065
	(0.155)	(0.170)	(0.156)
Transmen	-0.163	-0.164	-0.309
	(0.147)	(0.161)	(0.265)
Transwomen	0.270	0.235	0.162
	(0.205)	(0.205)	(0.290)
Rem. feminine words	0.058	0.062	0.060
	(0.037)	(0.037)	(0.037)
Rem. masculine words	0.101 *	0.103 *	0.100 *
	(0.041)	(0.041)	(0.041)
Rem. feminine words x Ciswomen	-0.031	-0.036	-0.031
	(0.046)	(0.046)	(0.046)
Rem. masculine words x Ciswomen	-0.064	-0.070	-0.062
	(0.052)	(0.052)	(0.052)
Rem. feminine words x Transmen	-0.019	-0.026	-0.022
	(0.046)	(0.046)	(0.046)
Rem. masculine words x Transmen	-0.092	-0.099 *	-0.092
	(0.049)	(0.049)	(0.049)
Rem. feminine words x Transwomen	-0.082	-0.084	-0.079
	(0.056)	(0.056)	(0.056)
Rem. masculine words x Transwomen	-0.056	-0.054	-0.057
	(0.061)	(0.062)	(0.061)
Const.	1.798 ***	1.552	1.752 ***
	(0.110)	(0.906)	(0.300)
Controls (Age, Height, Student status, Income, Religion, Residence)	–	Yes	–
Controls (TCS, STT)	–	–	Yes
N	780	780	780
Adj. R2	0.019	0.015	0.019
H_0: Rem. feminine words on Cismen	0.114	0.092	0.104
H_0: Rem. masculine words on Cismen	0.014	0.012	0.014
H_0: Rem. feminine words on Ciswomen	0.334	0.362	0.314
H_0: Rem. masculine words on Ciswomen	0.259	0.319	0.244
H_0: Rem. feminine words on Transmen	0.158	0.196	0.166
H_0: Rem. masculine words on Transmen	0.755	0.867	0.766
H_0: Rem. feminine words on Transwomen	0.558	0.587	0.640
H_0: Rem. masculine words on Transwomen	0.330	0.285	0.353

Note: Standard errors in parentheses are heteroskedasticity robust. In the second last column from the right, the baseline is a non-student, non-religious cisman, who earns more than 20K GBP, and lives in continental Europe. In the last column from the right, the baseline is a cisman. *** $p < 0.001$; ** $p < 0.01$; * $p < 0.05$. Rows starting with H_0 report the p-values of a joint coefficient test that the coefficients' sum equals 0. For example, "H_0: Rem. feminine words on Ciswomen" tests the effect of Rem. feminine words on the subject group (Ciswomen).

10.3 Altruism

Supplementary Table 36 OLS regression for all treatments. Dependent variable: Donations.

	(1)	(2)	(3)
Ciswomen	0.007	0.025	0.012
	(0.285)	(0.303)	(0.285)
Transmen	0.122	0.230	-0.014
	(0.284)	(0.298)	(0.420)
Transwomen	-0.225	-0.164	-0.403
	(0.324)	(0.324)	(0.438)
Rem. feminine words	-0.112	-0.115	-0.113
	(0.062)	(0.065)	(0.062)
Rem. masculine words	-0.054	-0.063	-0.055
	(0.071)	(0.073)	(0.071)
Rem. feminine words x Ciswomen	0.212 **	0.218 **	0.211 **
	(0.080)	(0.082)	(0.080)
Rem. masculine words x Ciswomen	0.100	0.123	0.098
	(0.090)	(0.092)	(0.090)
Rem. feminine words x Transmen	0.117	0.113	0.115
	(0.080)	(0.081)	(0.081)
Rem. masculine words x Transmen	0.032	0.041	0.030
	(0.090)	(0.090)	(0.090)
Rem. feminine words x Transwomen	0.141	0.164	0.139
	(0.096)	(0.097)	(0.096)
Rem. masculine words x Transwomen	0.084	0.093	0.085
	(0.102)	(0.102)	(0.103)
Const.	2.695 ***	1.848	3.150 ***
	(0.202)	(1.396)	(0.506)
Controls (Age, Height, Student status, Income, Religion, Residence)	–	Yes	–
Controls (TCS, STT)	–	–	Yes
N	780	780	780
Adj. R2	0.005	0.030	0.004
H_0: Rem. feminine words on Cismen	0.072	0.076	0.071
H_0: Rem. masculine words on Cismen	0.450	0.390	0.441
H_0: Rem. feminine words on Ciswomen	0.049	0.041	0.051
H_0: Rem. masculine words on Ciswomen	0.412	0.279	0.433
H_0: Rem. feminine words on Transmen	0.921	0.975	0.971
H_0: Rem. masculine words on Transmen	0.697	0.684	0.659
H_0: Rem. feminine words on Transwomen	0.693	0.492	0.717
H_0: Rem. masculine words on Transwomen	0.685	0.673	0.681

Note: Standard errors in parentheses are heteroskedasticity robust. In the second last column from the right, the baseline is a non-student, non-religious cisman, who earns more than 20K GBP, and lives in continental Europe. In the last column from the right, the baseline is a cisman. *** $p < 0.001$; ** $p < 0.01$; * $p < 0.05$. Rows starting with H_0 report the p-values of a joint coefficient test that the coefficients' sum equals 0. For example, "H_0: Rem. feminine words on Ciswomen" tests the effect of Rem. feminine words on the subject group (Ciswomen).

11 Participants remembered at least 4 words

11.1 Competitiveness

Supplementary Table 37 Probit regression for all treatments. Dependent variable: Competition. The participants remembered at least 4 words in Part 1.

	(1)	(2)	(3)	(4)	(5)
Ciswomen	0.047	0.066	0.197	0.357	0.158
	(0.317)	(0.330)	(0.344)	(0.363)	(0.346)
Transmen	0.065	0.117	0.220	0.286	0.554
	(0.318)	(0.329)	(0.320)	(0.378)	(0.435)
Transwomen	0.122	0.223	0.208	0.264	0.650
	(0.377)	(0.392)	(0.416)	(0.413)	(0.516)
Treatment: FEMININE	0.361	0.189	0.158	0.212	0.136
	(0.306)	(0.309)	(0.302)	(0.326)	(0.305)
Treatment: MASCULINE	0.331	0.351	0.272	0.434	0.247
	(0.312)	(0.325)	(0.325)	(0.349)	(0.327)
FEMININE x Ciswomen	-0.378	-0.139	-0.094	-0.112	-0.055
	(0.404)	(0.421)	(0.446)	(0.465)	(0.448)
MASCULINE x Ciswomen	-0.413	-0.455	-0.383	-0.471	-0.327
	(0.412)	(0.432)	(0.449)	(0.459)	(0.450)
FEMININE x Transmen	-0.558	-0.375	-0.284	-0.314	-0.222
	(0.405)	(0.415)	(0.409)	(0.431)	(0.409)
MASCULINE x Transmen	-0.592	-0.660	-0.469	-0.496	-0.406
	(0.414)	(0.428)	(0.434)	(0.461)	(0.441)
FEMININE x Transwomen	-0.128	-0.021	0.139	0.053	0.132
	(0.466)	(0.476)	(0.494)	(0.492)	(0.507)
MASCULINE x Transwomen	-0.533	-0.707	-0.651	-0.779	-0.638
	(0.479)	(0.499)	(0.521)	(0.534)	(0.533)
Perf. tournament		0.006	-0.056 **	-0.059 **	-0.061 **
		(0.017)	(0.021)	(0.022)	(0.021)
Delta perf.		0.147 ***	0.114 ***	0.129 ***	0.118 ***
		(0.024)	(0.027)	(0.027)	(0.027)
Belief tournament			-0.602 ***	-0.612 ***	-0.604 ***
			(0.088)	(0.091)	(0.089)
Risk			0.165 **	0.166 **	0.164 **
			(0.054)	(0.055)	(0.054)
Const.	-0.595 *	-0.759 *	0.790	-2.259	-0.228
	(0.251)	(0.305)	(0.439)	(1.839)	(0.594)
Controls (Age, Height, Student status, Income, Religion, Residence)	–	–	–	Yes	–
Controls (TCS, STT)	–	–	–	–	Yes
N	546	546	546	546	546
Pseudo R2 (McFadden)	0.017	0.104	0.189	0.231	0.200
H_0: FEMININE on Cismen	0.232	0.546	0.631	0.530	0.682
H_0: MASCULINE on Cismen	0.283	0.274	0.419	0.215	0.463
H_0: FEMININE on Ciswomen	0.947	0.853	0.822	0.732	0.778
H_0: MASCULINE on Ciswomen	0.758	0.710	0.704	0.902	0.786
H_0: FEMININE on Transmen	0.452	0.496	0.661	0.731	0.766
H_0: MASCULINE on Transmen	0.332	0.275	0.513	0.841	0.604
H_0: FEMININE on Transwomen	0.502	0.640	0.430	0.480	0.481
H_0: MASCULINE on Transwomen	0.574	0.336	0.327	0.373	0.317

Note: Competition is a binary variable equal to 1 if the participant enters the tournament in Part 4 and 0 otherwise. Delta perf. is the difference in performance between Part 3 (tournament) and Part 2 (piece-rate). Standard errors in parentheses are heteroskedasticity robust. In the second last column from the right, the baseline is a non-student, non-religious cisman, who earns more than 20K GBP, and lives in continental Europe. In the last column from the right, the baseline is a cisman. *** $p < 0.001$; ** $p < 0.01$; * $p < 0.05$. Rows starting with H_0 report the p-values of a joint coefficient test that the coefficients' sum equals 0. For example, "H_0: FEMININE on Ciswomen" tests the effect of the treatment (FEMININE) on the subject group (Ciswomen).

11.2 Risk

Supplementary Table 38 OLS regression for all treatments. Dependent variable: Risk. The participants remembered at least 4 words in Part 1.

	(1)	(2)	(3)
Ciswomen	-0.148	-0.142	-0.154
	(0.284)	(0.297)	(0.285)
Transmen	-0.192	-0.217	-0.430
	(0.293)	(0.306)	(0.414)
Transwomen	0.083	0.069	-0.107
	(0.333)	(0.338)	(0.411)
Treatment: FEMININE	0.228	0.260	0.247
	(0.294)	(0.291)	(0.294)
Treatment: MASCULINE	0.525	0.577	0.517
	(0.299)	(0.300)	(0.299)
FEMININE x Ciswomen	-0.094	-0.120	-0.096
	(0.361)	(0.358)	(0.362)
MASCULINE x Ciswomen	-0.364	-0.417	-0.340
	(0.362)	(0.361)	(0.363)
FEMININE x Transmen	-0.081	-0.116	-0.093
	(0.364)	(0.362)	(0.365)
MASCULINE x Transmen	-0.469	-0.535	-0.459
	(0.369)	(0.370)	(0.370)
FEMININE x Transwomen	-0.289	-0.349	-0.262
	(0.415)	(0.418)	(0.411)
MASCULINE x Transwomen	-0.229	-0.236	-0.216
	(0.436)	(0.440)	(0.433)
Const.	1.883 ***	1.775	1.844 ***
	(0.240)	(1.280)	(0.416)
Controls (Age, Height, Student status, Income, Religion, Residence)	–	Yes	–
Controls (TCS, STT)	–	–	Yes
N	546	546	546
Adj. R2	0.014	0.008	0.015
H_0: FEMININE on Cismen	0.437	0.373	0.402
H_0: MASCULINE on Cismen	0.080	0.055	0.084
H_0: FEMININE on Ciswomen	0.524	0.514	0.475
H_0: MASCULINE on Ciswomen	0.432	0.452	0.390
H_0: FEMININE on Transmen	0.494	0.512	0.481
H_0: MASCULINE on Transmen	0.798	0.854	0.790
H_0: FEMININE on Transwomen	0.836	0.764	0.956
H_0: MASCULINE on Transwomen	0.353	0.291	0.338

Note: Standard errors in parentheses are heteroskedasticity robust. In the second last column from the right, the baseline is a non-student, non-religious cisman, who earns more than 20K GBP, and lives in continental Europe. In the last column from the right, the baseline is a cisman. *** $p < 0.001$; ** $p < 0.01$; * $p < 0.05$. Rows starting with H_0 report the p-values of a joint coefficient test that the coefficients' sum equals 0. For example, "H_0: FEMININE on Ciswomen" tests the effect of the treatment (FEMININE) on the subject group (Ciswomen).

11.3 Altruism

Supplementary Table 39 OLS regression for all treatments. Dependent variable: Donations. The participants remembered at least 4 words in Part 1.

	(1)	(2)	(3)
Ciswomen	0.327	0.389	0.342
	(0.469)	(0.500)	(0.472)
Transmen	0.487	0.579	0.099
	(0.497)	(0.526)	(0.651)
Transwomen	-0.243	-0.116	-0.670
	(0.541)	(0.555)	(0.669)
Treatment: FEMININE	-0.349	-0.360	-0.335
	(0.477)	(0.490)	(0.478)
Treatment: MASCULINE	-0.079	-0.135	-0.082
	(0.484)	(0.499)	(0.484)
FEMININE x Ciswomen	0.745	0.769	0.726
	(0.604)	(0.609)	(0.605)
MASCULINE x Ciswomen	0.093	0.204	0.080
	(0.612)	(0.618)	(0.613)
FEMININE x Transmen	0.178	0.189	0.141
	(0.619)	(0.618)	(0.619)
MASCULINE x Transmen	-0.253	-0.149	-0.256
	(0.632)	(0.639)	(0.632)
FEMININE x Transwomen	0.591	0.669	0.603
	(0.704)	(0.716)	(0.705)
MASCULINE x Transwomen	0.204	0.199	0.218
	(0.701)	(0.718)	(0.704)
Const.	2.528 ***	1.844	3.301 ***
	(0.384)	(1.901)	(0.675)
Controls (Age, Height, Student status, Income, Religion, Residence)	–	Yes	–
Controls (TCS, STT)	–	–	Yes
N	546	546	546
Adj. R2	0.004	0.015	0.004
H_0: FEMININE on Cismen	0.465	0.462	0.485
H_0: MASCULINE on Cismen	0.870	0.786	0.866
H_0: FEMININE on Ciswomen	0.286	0.266	0.293
H_0: MASCULINE on Ciswomen	0.971	0.853	0.996
H_0: FEMININE on Transmen	0.665	0.652	0.625
H_0: MASCULINE on Transmen	0.415	0.475	0.407
H_0: FEMININE on Transwomen	0.640	0.556	0.606
H_0: MASCULINE on Transwomen	0.805	0.902	0.790

Note: Standard errors in parentheses are heteroskedasticity robust. In the second last column from the right, the baseline is a non-student, non-religious cisman, who earns more than 20K GBP, and lives in continental Europe. In the last column from the right, the baseline is a cisman. *** $p < 0.001$; ** $p < 0.01$; * $p < 0.05$. Rows starting with H_0 report the p-values of a joint coefficient test that the coefficients' sum equals 0. For example, "H_0: FEMININE on Ciswomen" tests the effect of the treatment (FEMININE) on the subject group (Ciswomen).

12 Participants remembered less than 4 words

12.1 Competitiveness

Supplementary Table 40 Probit regression for all treatments. Dependent variable: Competition. The participants remembered less than 4 words in Part 1.

	(1)	(2)	(3)	(4)	(5)
Ciswomen	0.176	0.327	0.481	0.450	0.444
	(0.340)	(0.352)	(0.417)	(0.453)	(0.408)
Transmen	-0.254	-0.235	-0.094	-0.184	0.671
	(0.348)	(0.362)	(0.401)	(0.421)	(0.594)
Transwomen	-0.021	0.047	-0.051	-0.307	0.653
	(0.351)	(0.362)	(0.363)	(0.384)	(0.572)
Treatment: FEMININE	0.075	0.122	-0.034	-0.241	-0.076
	(0.380)	(0.425)	(0.440)	(0.475)	(0.451)
Treatment: MASCULINE	0.616	0.746 *	0.729 *	0.870 *	0.705 *
	(0.338)	(0.335)	(0.348)	(0.400)	(0.351)
FEMININE x Ciswomen	-0.590	-0.771	-0.844	-0.699	-0.721
	(0.606)	(0.655)	(0.740)	(0.800)	(0.745)
MASCULINE x Ciswomen	-0.882	-1.096 *	-1.124	-1.140	-1.050
	(0.545)	(0.551)	(0.606)	(0.687)	(0.612)
FEMININE x Transmen	0.421	0.431	0.554	0.639	0.631
	(0.603)	(0.681)	(0.672)	(0.708)	(0.689)
MASCULINE x Transmen	0.006	-0.116	-0.029	0.177	-0.002
	(0.552)	(0.543)	(0.543)	(0.616)	(0.561)
FEMININE x Transwomen	-0.338	-0.200	0.095	0.643	-0.206
	(0.617)	(0.641)	(0.630)	(0.637)	(0.647)
MASCULINE x Transwomen	-0.668	-0.857	-0.583	-0.741	-0.598
	(0.616)	(0.618)	(0.616)	(0.636)	(0.628)
Perf. tournament		-0.038	-0.097 ***	-0.109 ***	-0.088 **
		(0.024)	(0.029)	(0.033)	(0.030)
Delta perf.		0.118 **	0.105 **	0.107 **	0.104 **
		(0.036)	(0.038)	(0.041)	(0.039)
Belief tournament			-0.493 ***	-0.531 ***	-0.467 ***
			(0.129)	(0.148)	(0.132)
Risk			0.172	0.179	0.169
			(0.092)	(0.099)	(0.093)
Const.	-0.452 *	-0.273	0.975	0.666	0.490
	(0.204)	(0.291)	(0.509)	(2.158)	(0.944)
Controls (Age, Height, Student status, Income, Religion, Residence)	–	–	–	Yes	–
Controls (TCS, STT)	–	–	–	–	Yes
N	234	234	234	234	234
Pseudo R2 (McFadden)	0.032	0.076	0.147	0.214	0.160
H_0: FEMININE on Cismen	0.840	0.750	0.931	0.551	0.849
H_0: MASCULINE on Cismen	0.061	0.029	0.036	0.024	0.043
H_0: FEMININE on Ciswomen	0.262	0.167	0.080	0.073	0.114
H_0: MASCULINE on Ciswomen	0.523	0.409	0.375	0.563	0.440
H_0: FEMININE on Transmen	0.278	0.232	0.273	0.432	0.252
H_0: MASCULINE on Transmen	0.143	0.144	0.115	0.032	0.118
H_0: FEMININE on Transwomen	0.579	0.873	0.910	0.483	0.627
H_0: MASCULINE on Transwomen	0.918	0.829	0.787	0.822	0.844

Note: Competition is a binary variable equal to 1 if the participant enters the tournament in Part 4 and 0 otherwise. Delta perf. is the difference in performance between Part 3 (tournament) and Part 2 (piece-rate). Belief tournament is the participants' belief of their performance rank within their group in Part 3, where the value 1 represents the rank with the highest performance. Standard errors in parentheses are heteroskedasticity robust. In the second last column from the right, the baseline is a non-student, non-religious cisman, who earns more than 20K GBP, and lives in continental Europe. In the last column from the right, the baseline is a cisman. *** $p < 0.001$; ** $p < 0.01$; * $p < 0.05$. Rows starting with H_0 report the p-values of a joint coefficient test that the coefficients' sum equals 0. For example, "H_0: FEMININE on Ciswomen" tests the effect of the treatment (FEMININE) on the subject group (Ciswomen).

12.2 Risk

Supplementary Table 41 OLS regression for all treatments. Dependent variable: Risk. The participants remembered less than 4 words in Part 1.

	(1)	(2)	(3)
Ciswomen	-0.170	-0.141	-0.188
	(0.247)	(0.268)	(0.252)
Transmen	-0.128	-0.107	0.161
	(0.196)	(0.216)	(0.435)
Transwomen	0.755 *	0.651	1.044 *
	(0.370)	(0.392)	(0.510)
Treatment: FEMININE	-0.032	-0.053	-0.054
	(0.262)	(0.283)	(0.265)
Treatment: MASCULINE	0.026	0.036	0.014
	(0.236)	(0.249)	(0.234)
FEMININE x Ciswomen	0.220	0.258	0.275
	(0.472)	(0.482)	(0.479)
MASCULINE x Ciswomen	0.588	0.471	0.621
	(0.395)	(0.409)	(0.397)
FEMININE x Transmen	0.101	0.167	0.117
	(0.406)	(0.429)	(0.411)
MASCULINE x Transmen	-0.349	-0.461	-0.345
	(0.322)	(0.340)	(0.324)
FEMININE x Transwomen	-0.837	-0.734	-0.909
	(0.507)	(0.536)	(0.499)
MASCULINE x Transwomen	-0.449	-0.337	-0.462
	(0.676)	(0.662)	(0.671)
Const.	1.768 ***	2.141	1.537 **
	(0.157)	(1.294)	(0.542)
Controls (Age, Height, Student status, Income, Religion, Residence)	–	Yes	–
Controls (TCS, STT)	–	–	Yes
N	234	234	234
Adj. R2	0.032	0.029	0.027
H_0: FEMININE on Cismen	0.902	0.851	0.840
H_0: MASCULINE on Cismen	0.913	0.886	0.951
H_0: FEMININE on Ciswomen	0.633	0.597	0.580
H_0: MASCULINE on Ciswomen	0.054	0.121	0.049
H_0: FEMININE on Transmen	0.826	0.730	0.843
H_0: MASCULINE on Transmen	0.142	0.072	0.145
H_0: FEMININE on Transwomen	0.047	0.081	0.024
H_0: MASCULINE on Transwomen	0.505	0.624	0.478

Note: Standard errors in parentheses are heteroskedasticity robust. In the second last column from the right, the baseline is a non-student, non-religious cisman, who earns more than 20K GBP, and lives in continental Europe. In the last column from the right, the baseline is a cisman. *** $p < 0.001$; ** $p < 0.01$; * $p < 0.05$. Rows starting with H_0 report the p-values of a joint coefficient test that the coefficients' sum equals 0. For example, "H_0: FEMININE on Ciswomen" tests the effect of the treatment (FEMININE) on the subject group (Ciswomen).

12.3 Altruism

Supplementary Table 42 OLS regression for all treatments. Dependent variable: Donations. The participants remembered less than 4 words in Part 1.

	(1)	(2)	(3)
Ciswomen	-0.095	0.081	-0.126
	(0.547)	(0.625)	(0.554)
Transmen	-0.505	-0.169	-0.034
	(0.479)	(0.482)	(0.695)
Transwomen	0.155	0.193	0.628
	(0.539)	(0.557)	(0.705)
Treatment: FEMININE	-0.252	-0.076	-0.287
	(0.500)	(0.545)	(0.506)
Treatment: MASCULINE	-0.421	0.009	-0.440
	(0.519)	(0.559)	(0.520)
FEMININE x Ciswomen	0.360	0.231	0.449
	(0.849)	(0.879)	(0.867)
MASCULINE x Ciswomen	0.578	0.133	0.632
	(0.818)	(0.873)	(0.820)
FEMININE x Transmen	0.675	0.462	0.700
	(0.810)	(0.797)	(0.807)
MASCULINE x Transmen	1.335	0.723	1.342
	(0.775)	(0.765)	(0.775)
FEMININE x Transwomen	-0.055	-0.093	-0.171
	(0.828)	(0.857)	(0.816)
MASCULINE x Transwomen	0.416	0.291	0.394
	(1.016)	(0.969)	(1.014)
Const.	2.791 ***	0.629	2.396 **
	(0.322)	(2.381)	(0.915)
Controls (Age, Height, Student status, Income, Religion, Residence)	–	Yes	–
Controls (TCS, STT)	–	–	Yes
N	234	234	234
Adj. R2	-0.033	0.006	-0.039
H_0: FEMININE on Cismen	0.614	0.889	0.571
H_0: MASCULINE on Cismen	0.418	0.987	0.399
H_0: FEMININE on Ciswomen	0.875	0.825	0.817
H_0: MASCULINE on Ciswomen	0.804	0.831	0.762
H_0: FEMININE on Transmen	0.508	0.514	0.515
H_0: MASCULINE on Transmen	0.114	0.184	0.118
H_0: FEMININE on Transwomen	0.642	0.796	0.480
H_0: MASCULINE on Transwomen	0.995	0.710	0.959

Note: Standard errors in parentheses are heteroskedasticity robust. In the second last column from the right, the baseline is a non-student, non-religious cisman, who earns more than 20K GBP, and lives in continental Europe. In the last column from the right, the baseline is a cisman. *** $p < 0.001$; ** $p < 0.01$; * $p < 0.05$. Rows starting with H_0 report the p-values of a joint coefficient test that the coefficients' sum equals 0. For example, "H_0: FEMININE on Ciswomen" tests the effect of the treatment (FEMININE) on the subject group (Ciswomen).

13 Gender congruent upbringing

13.1 Competitiveness

Supplementary Table 43 Probit regression for NEUTRAL. Dependent variable: Competition. Only transgender participants are considered.

	(1)	(2)	(3)	(4)	(5)
Gender congruent upbringing	0.054	0.004	-0.118	-0.279	-0.187
	(0.295)	(0.301)	(0.353)	(0.427)	(0.375)
Perf. tournament		0.017	-0.103	-0.095	-0.100
		(0.039)	(0.056)	(0.062)	(0.058)
Delta perf.		0.101	0.060	0.033	0.056
		(0.056)	(0.066)	(0.080)	(0.068)
Belief tournament			-0.819 ***	-0.958 ***	-0.793 ***
			(0.220)	(0.272)	(0.228)
Risk			0.252 *	0.298 *	0.269 *
			(0.111)	(0.127)	(0.108)
Age				0.022	
				(0.018)	
Height				-0.001	
				(0.012)	
Student status				-0.292	
				(0.356)	
Income: < 20,000 GBP				0.230	
				(0.422)	
Religion: Religious				0.327	
				(0.361)	
Religion: Not say				6.768 ***	
				(0.499)	
Residence: US				0.027	
				(0.501)	
Residence: UK				0.104	
				(0.450)	
Residence: Other				0.226	
				(0.519)	
TCS					0.168
					(0.200)
STT					-0.028
					(0.042)
Const.	-0.557 ***	-0.735 *	1.713	1.172	1.385
	(0.141)	(0.349)	(0.938)	(2.643)	(1.063)
N	116	116	116	116	116
Pseudo R2 (McFadden)	0.000	0.048	0.196	0.306	0.202

Note: Competition is a binary variable equal to 1 if the participant enters the tournament in Part 4 and 0 otherwise. Delta perf. is the difference in performance between Part 3 (tournament) and Part 2 (piece-rate). Belief tournament is the participants' belief of their performance rank within their group in Part 3, where the value 1 represents the rank with the highest performance. Gender congruent upbringing is a binary variable equal to 1 if the way the participant's parents treated the participant matches the reported gender of the participant or the parents treated their child neutrally. Standard errors in parentheses are heteroskedasticity robust. In the second last column from the right, the baseline is a non-student, non-religious person, who earns more than 20K GBP, and lives in continental Europe. *** $p < 0.001$; ** $p < 0.01$; * $p < 0.05$.

Supplementary Table 44 Probit regression for all treatments. Dependent variable: Competition. Only transgender participants are considered.

	(1)	(2)	(3)	(4)	(5)
Gender congruent upbringing	0.054	0.005	-0.108	-0.120	-0.279
	(0.295)	(0.318)	(0.344)	(0.381)	(0.358)
Treatment: FEMININE	0.035	0.056	0.182	0.204	0.143
	(0.197)	(0.209)	(0.217)	(0.217)	(0.221)
Treatment: MASCULINE	-0.099	-0.161	-0.083	-0.081	-0.056
	(0.207)	(0.212)	(0.233)	(0.248)	(0.237)
FEMININE x Gender congruent upbringing	-0.028	0.021	-0.102	-0.087	0.056
	(0.408)	(0.431)	(0.471)	(0.505)	(0.482)
MASCULINE x Gender congruent upbringing	0.035	0.075	0.155	0.192	0.161
	(0.402)	(0.421)	(0.450)	(0.490)	(0.468)
Perf. tournament		-0.022	-0.114 ***	-0.117 ***	-0.111 ***
		(0.022)	(0.029)	(0.029)	(0.030)
Delta perf.		0.162 ***	0.132 ***	0.141 ***	0.135 ***
		(0.032)	(0.036)	(0.038)	(0.035)
Belief tournament			-0.727 ***	-0.730 ***	-0.705 ***
			(0.112)	(0.120)	(0.114)
Risk			0.196 **	0.222 **	0.194 **
			(0.065)	(0.069)	(0.065)
Const.	-0.557 ***	-0.430	1.669 ***	-0.365	1.139 *
	(0.141)	(0.229)	(0.482)	(1.433)	(0.549)
Controls (Age, Height, Student status, Income, Religion, Residence)	–	–	–	Yes	–
Controls (TCS, STT)	–	–	–	–	Yes
N	355	355	355	355	355
Pseudo R2 (McFadden)	0.001	0.079	0.198	0.233	0.209
H_0: FEMININE on GCU	0.983	0.833	0.840	0.778	0.624
H_0: MASCULINE on GCU	0.852	0.809	0.852	0.781	0.791

Note: Competition is a binary variable equal to 1 if the participant enters the tournament in Part 4 and 0 otherwise. Delta perf. is the difference in performance between Part 3 (tournament) and Part 2 (piece-rate). Belief tournament is the participants' belief of their performance rank within their group in Part 3, where the value 1 represents the rank with the highest performance. Gender congruent upbringing is a binary variable equal to 1 if the way the participant's parents treated the participant matches the reported gender of the participant or the parents treated their child neutrally. Standard errors in parentheses are heteroskedasticity robust. Rows starting with H_0 report the p-values of a joint coefficient test that the coefficients' sum equals 0. For example, "H0: FEMININE on GCU" tests the effect of the treatment (FEMININE) on the group of participants that were brought up gender congruently (Gender congruent upbringing=1).

13.2 Risk

Supplementary Table 45 OLS regression for NEUTRAL. Dependent variable: Risk. Only transgender participants are considered.

	(1)	(2)	(3)
Gender congruent upbringing	-0.529 *	-0.485 *	-0.445
	(0.236)	(0.236)	(0.285)
Age		-0.006	
		(0.016)	
Height		0.000	
		(0.014)	
Student status		0.121	
		(0.260)	
Income: < 20,000 GBP		-0.008	
		(0.266)	
Religion: Religious		0.244	
		(0.228)	
Religion: Not say		-0.597	
		(0.559)	
Residence: US		-0.091	
		(0.341)	
Residence: UK		0.026	
		(0.312)	
Residence: Other		0.845 *	
		(0.425)	
TCS			-0.066
			(0.150)
STT			-0.019
			(0.037)
Const.	2.008 ***	1.894	2.343 ***
	(0.126)	(2.359)	(0.415)
N	116	116	116
Adj. R2	0.027	0.032	0.019

Note: Gender congruent upbringing is a binary variable equal to 1 if the way the participant's parents treated the participant matches the reported gender of the participant or the parents treated their child neutrally. Standard errors in parentheses are heteroskedasticity robust. In the second last column from the right, the baseline is a non-student, non-religious person, who earns more than 20K GBP, and lives in continental Europe. *** $p < 0.001$; ** $p < 0.01$; * $p < 0.05$.

Supplementary Table 46 OLS regression for all treatments. Dependent variable: Risk. Only transgender participants are considered.

	(1)	(2)	(3)
Gender congruent upbringing	-0.529 *	-0.535 *	-0.588 *
	(0.236)	(0.236)	(0.255)
Treatment: FEMININE	-0.229	-0.201	-0.239
	(0.166)	(0.163)	(0.169)
Treatment: MASCULINE	-0.144	-0.116	-0.138
	(0.184)	(0.184)	(0.184)
FEMININE x Gender congruent upbringing	0.726 *	0.737 *	0.773 *
	(0.323)	(0.321)	(0.335)
MASCULINE x Gender congruent upbringing	0.583	0.556	0.582
	(0.333)	(0.334)	(0.335)
Const.	2.008 ***	0.453	1.825 ***
	(0.126)	(1.201)	(0.238)
Controls (Age, Height, Student status, Income, Religion, Residence)	–	Yes	–
Controls (TCS, STT)	–	–	Yes
N	355	355	355
Adj. R2	0.001	0.014	-0.001
H_0: FEMININE on GCU	0.074	0.055	0.063
H_0: MASCULINE on GCU	0.116	0.115	0.115

Note: Gender congruent upbringing is a binary variable equal to 1 if the way the participant's parents treated the participant matches the reported gender of the participant or the parents treated their child neutrally. Standard errors in parentheses are heteroskedasticity robust. Rows starting with H_0 report the p-values of a joint coefficient test that the coefficients' sum equals 0. For example, "H0: FEMININE on GCU" tests the effect of the treatment (FEMININE) on the group of participants that were brought up gender congruently (Gender congruent upbringing=1).

13.3 Altruism

Supplementary Table 47 OLS regression for NEUTRAL. Dependent variable: Donations. Only transgender participants are considered.

	(1)	(2)	(3)
Gender congruent upbringing	0.141	0.019	0.248
	(0.428)	(0.423)	(0.486)
Age		0.056 *	
		(0.024)	
Height		-0.012	
		(0.016)	
Student status		0.917 *	
		(0.392)	
Income: < 20,000 GBP		0.540	
		(0.419)	
Religion: Religious		1.100 **	
		(0.362)	
Religion: Not say		1.344	
		(1.498)	
Residence: US		-0.735	
		(0.538)	
Residence: UK		-0.335	
		(0.553)	
Residence: Other		-0.303	
		(0.663)	
TCS			-0.174
			(0.259)
STT			0.013
			(0.054)
Const.	2.675 ***	2.480	3.013 ***
	(0.214)	(2.900)	(0.668)
N	116	116	116
Adj. R2	-0.008	0.087	-0.021

Note: Gender congruent upbringing is a binary variable equal to 1 if the way the participant's parents treated the participant matches the reported gender of the participant or the parents treated their child neutrally. Standard errors in parentheses are heteroskedasticity robust. In the second last column from the right, the baseline is a non-student, non-religious person, who earns more than 20K GBP, and lives in continental Europe. *** $p < 0.001$; ** $p < 0.01$; * $p < 0.05$.

Supplementary Table 48 OLS regression for all treatments. Dependent variable: Donations. Only transgender participants are considered.

	(1)	(2)	(3)
Gender congruent upbringing	0.141	0.069	0.229
	(0.428)	(0.410)	(0.449)
Treatment: FEMININE	-0.046	-0.004	-0.074
	(0.293)	(0.284)	(0.293)
Treatment: MASCULINE	-0.337	-0.343	-0.355
	(0.304)	(0.301)	(0.305)
FEMININE x Gender congruent upbringing	0.213	0.203	0.197
	(0.587)	(0.551)	(0.598)
MASCULINE x Gender congruent upbringing	1.029	1.158 *	1.038
	(0.568)	(0.547)	(0.568)
Const.	2.675 ***	2.464	3.038 ***
	(0.214)	(1.690)	(0.395)
Controls (Age, Height, Student status, Income, Religion, Residence)	–	Yes	–
Controls (TCS, STT)	–	–	Yes
N	355	355	355
Adj. R2	0.013	0.076	0.012
H_0: FEMININE on GCU	0.742	0.677	0.812
H_0: MASCULINE on GCU	0.150	0.076	0.155

Note: Gender congruent upbringing is a binary variable equal to 1 if the way the participant's parents treated the participant matches the reported gender of the participant or the parents treated their child neutrally. Standard errors in parentheses are heteroskedasticity robust. Rows starting with H_0 report the p-values of a joint coefficient test that the coefficients' sum equals 0. For example, "H0: FEMININE on GCU" tests the effect of the treatment (FEMININE) on the group of participants that were brought up gender congruently (Gender congruent upbringing=1).

14 Detailed literature summary

14.1 Competitiveness

Differences in competitiveness have become an essential explanation for labor market outcomes like variations in wages[2], and different demands in wage negotiations[3]. Pinning down the causes and consequences of the willingness to compete is important as it correlates with several relevant choices and characteristics for education and labor market outcomes[4]. For example, subjects who are more competitive have been found to be more likely to choose competitive educational programs[1,5–7], to have a higher income[8–10] and to become entrepreneurs[11]. But what role does one of the main human characteristics - being a man or a woman - play for competitiveness?

During the last decades, an impressive amount of scientific evidence showed that women are generally less competitive than men[12–18]. This gender gap in competitiveness (henceforth GGC) is robust when using different scientific methods. Studies report that men are more likely to compete when using classical lab[18], lab-in-the-field[19], field[20], and online experiments[1]. The findings also replicate when using subjects from different age groups like children[16], students[18], and non-students[21].

Recently some evidence has been collected on the lack of a GGC in certain circumstances. For example, for the matriarchy of Masai in Kenya, adult women are reported to be even more competitive than men[19]. Similarly, children living in the Khasi matrilineal society in northeast India are equally competitive[21]. Without the need to go afar, it has been shown that the type of school children attend influences competitiveness with female students from girl's schools being as competitive as boys[22]. Moreover, for children from families with lower socioeconomic backgrounds, no GGC is reported[15]. Also, cultural differences play a role in competitiveness, as shown by[23]. They found that children are equally competitive in Columbia, but boys in Sweden are more competitive than girls. These mentioned studies suggest that women's lower willingness to compete is not something that they are born with, but rather a behavioral preference that can be influenced by different factors and can thus be addressed to nurture rather than nature.

Support for this perspective is provided by research showing that the GGC can be closed or reversed when using interventions, which do not influence participants' biological makeup. For example, some studies change the institutional environment to resemble different affirmative action policies and obtain gender balance in competitive environments[18,24–26]. Others use the easy-to-implement intervention of priming ([27] and[28]) which encourages women to enter competitions more often. Moreover, giving feedback about relative performance[29] and the earnings implications related to competition avoidance[30] successfully increases women's entry rates, as well as when more experienced people advise strong-performing women to compete[31]. Besides, when the price of the competition benefits not the participants themselves, but their offspring, again no GGC has been observed[32].

However, it is also plausible that biological factors like genes and hormones may lead to different decisions of women and men and are also a primary driver of behavior. Thus, a new and still developing field of research focuses on competitiveness from a more elementary perspective by taking hormones into account. Up to now, there is only one study by[33], which causally analyses the effect of estrogen and progestin (by administrating oral contraceptives) on competitiveness. The authors find no impact of the two hormones on the willingness to compete. All other studies use self-reported hormonal measures by asking female participants about their menstrual cycle day and taking hormonal contraceptives to infer their hormonal level. Using self-reports is noisy (for a detailed discussion why this is the case see,[34]) and leads to mixed findings whether hormones play a role for competitiveness or not[8,35].

The existing evidence already provides results on what factors correlate with competitive behavior and how differences in competitiveness between men and women can be closed. However, this paper will be the first to test the robustness of the GGC when priming subjects with a specific gender identity. Moreover, we contribute to the literature by investigating the willingness to compete of transgender subjects. To the best of our knowledge, no economic experiments have been done using transgender participants. According to our review of the literature, considering the behavior of LGBTQ+ individuals is extremely rare in experimental economics. We only found one paper on homosexuality and competitiveness by[8]. These aspects point out our study's potential to expand the knowledge in the domain of competitive behavior.

14.2 Risk

Risk-taking is considered a fundamental determinant of individual behavior in different domains like health[36,37], stock market participation[38], saving decisions[39], occupational and self-employment choices[40], personal and household finance[41,42], education[43] and environmental decision making[44]. One strand of the literature in Behavioral Economics reports seemingly strong evidence for women preferring to take less risk compared to men[45]. This difference in risk-taking is robust when using different experimental methods to measure risk, such as lotteries[46], investment games[47] or card games[48]. It is also reported for subjects varying from children[23], to students[49], to non-students[50].

Moreover, the difference is not influenced by conducting the experiment in the lab or in other environments like on online platforms[50].

Another strand of the literature does not support that risk-taking differs by gender. Those papers mainly concentrate on different underlying methodologies than those used by the studies mentioned above. First, they claim that it is important to clearly distinguish between differences on the individual level (categorical differences between men and women) and patterns that appear only at the aggregate level (such as, e.g., statistically detectable different means)[51]. Second, using quantitative measures of substantive differences that are not yet that common in economic studies (such as Cohen's d) or measures of substantive overlap (like, e.g., the *Index of Similarity*) also results in not having a substantially large gender gap in risk-taking[51,52]. [52], e.g., claims that standardized differences in means across gender mostly amount to less than one standard deviation, and that the degree of overlap in distributions of risk-taking behavior of men and women is generally exceeding 80%. On-average differences between (cis-) men and women in behavior are smaller than sex differences in, e.g., height or throwing ability[53] and pale next to the effects of aspects such as cultural manipulations or gender priming (e.g.,[52]). These papers align with the so-called gender similarities hypothesis from the psychological literature which argues that males and females are similar on most, but not all, psychological dimensions[53]. [54] claims that one explanation for gender differences in risk-taking still being such a prominently repeated finding is that science is biased towards these results because of, e.g., exiting stereotypes or confirmation bias for existing publications.

Several studies analyze gender differences in risk preferences for sub-populations of managers[49,55,56] and find that females are similar or even less risk-averse than men. The reasons could be a selection or social learning and adaptive behavior to the job demands. To disentangle these different factors,[57] uses an online experiment with scientists. They vary the salience of either the private or the professional identity of the subjects. They report that priming the professional identity reduces the gender gap in risk-taking. Besides, the gender gap decreases with increasing age as female senior scientists choose riskier options in the treatment where the profession is made salient.

Also, attempts to explore the connection between biological factors and risk-taking are taken for the domain of risky behavior. First, studies are exploring the causal effect of hormones on behavior.[1] For example,[58] test for administered testosterone or estrogen affecting women's risk-taking. No effect of either testosterone or estrogen on risk-taking could be detected. In line, the study by[59] and[60] find no effect of testosterone on risk aversion.[33] take a comparative approach and administer an oral contraceptive or not. Again, no connection between hormones and behavior is reported. Second, studies test for the correlation between the variation in risk-taking and genes. On the one hand, for example,[61] find no relationship between the dopamine and the serotonin gene and risk-taking. On the other hand, studies using, for example, the twin methodology and genome-wide association techniques (GWAS) report genetic foundations for the willingness to take risk[62–64]. Third, a recent study by[65] showed that the intake of a small dose of Acetaminophen, a very popular pain killer, increases risk-taking.

Several researchers prime subjects and study the effect on risk-taking[66–70]. The study closest to our research is[71] which finds that making the subject's gender salient with a short questionnaire does not impact risk preferences. Also,[72] report an effect of gender priming through questions and stereotypical pictures only on male risk preferences.[73] prime financial professionals with their professional salience, which leads to a decrease in risk-taking in a high stakes investment game. With a similar subject pool,[74] find that individuals primed with a bust scenario are more risk-averse compared to those primed with a boom scenario.[75] test the robustness of the results of[74] with an Amazon Mechanical Turk subject pool. They report no evidence of priming influencing risk-taking.[76] primed individuals who were exposed to violence by asking them to either recall happy, fearful or neutral moments. They find that remembering frightening experiences leads to a higher preference for certainty.

The only related study we are aware of that investigates the risk-taking behavior of LGBTQ+ individuals is[8]. It analyzes risk preferences by asking the subjects about their risk perception (survey question). It finds no significant differences between homosexual and heterosexual men and homosexual and heterosexual women.

14.3 Altruism

To what extend someone is pro-social, i.e., altruistic, is argued to explain behavior in the labor market, how individuals vote, if they take up volunteer work or not, and how willing someone is to give to a charity[77]. Altruistic behavior is typically measured with a dictator game, where participants are asked how much they want to transfer to an anonymous other participant[78,79], or how much they wish to donate to a charity[80]. It is a robust finding that participants in experiments transfer quite a substantial part of their endowment in dictator games, thus act altruisticly[81]. The literature reports mixed findings on the external validity of those experiments. One strand of the

[1]For our literature review, we summarize only studies that concentrate on pharmacological testosterone administration with double-blind placebo-controlled designs, which allows us to interpret results causally.

literature finds that individuals behave in donation experiments similar as in naturally occurring decision situations on charitable giving[82,83]. Other research contradicts these findings, as recently summarized by[84].

Concerning the level of altruism exhibited by men and women, a wide range of studies shows that women are generally more generous in dictator games. See, e.g.,[77] for an up-to-date meta-analysis of the existing literature on gender differences in charitable giving. These authors report that the magnitude of the gender differences in altruism is sensible to the experimental context. For example, the difference is more prominent if the dictator decides to donate to a charity than giving to an anonymous recipient. However, the difference is more minor if the dictator chooses between giving all or nothing compared to deciding on a continuous scale.

Turning to studies attempting to link hormones to altruism causally,[60] and[85] found no impact of administered testosterone on dictators' giving.[58] used another approach and administered testosterone, estrogen, or a placebo to the experimental participants. Again, no connection between either hormone or altruism is reported. Moreover, administering an oral contraceptive containing synthetic progesterone as the main ingredient suggests no hormonal impact on altruism levels. However, there is evidence that the underlying genes influence altruism. See for example[86] who used twins for their study.

Multiple studies explore if different priming influences altruistic behavior. For example, subsequent donations are affected by religious primes[87–90], by reminding subjects of secular, moral institutions[88], and by priming with subtle cues of observability[91–93].[94] report an increased gender gap in altruism when making gender more salient by requiring participants to specify their gender before the dictator game and informing them about the gender of the recipient. Again, we have found no published studies of altruism of LGBTQ+ individuals in economics.

References

1. Buser, T., Niederle, M. & Oosterbeek, H. Can competitiveness predict education and labor market outcomes? Evidence from incentivized choice and survey measures. Working Paper 28916, National Bureau of Economic Research (2021). DOI: 10.3386/w28916.
2. Card, D., Cardoso, A. R. & Kline, P. Bargaining, sorting, and the gender wage gap: Quantifying the impact of firms on the relative pay of women. *The Q. J. Econ.* **131**, 633–686 (2016).
3. Leibbrandt, A. & List, J. A. Do women avoid salary negotiations? evidence from a large-scale natural field experiment. *Manag. Sci.* **61**, 2016–2024 (2015).
4. Shurchkov, O. & Eckel, C. C. *Gender differences in behavioral traits and labor market outcomes* (Oxford, UK: Oxford University Press, 2018).
5. Reuben, E., Wiswall, M. & Zafar, B. Preferences and biases in educational choices and labour market expectations: Shrinking the black box of gender. *The Econ. J.* **127**, 2153–2186 (2017).
6. Almås, I., Cappelen, A. W., Salvanes, K. G., Sørensen, E. Ø. & Tungodden, B. What explains the gender gap in college track dropout? Experimental and administrative evidence. *Am. Econ. Rev.* **106**, 296–302 (2016).
7. Buser, T., Niederle, M. & Oosterbeek, H. Gender, competitiveness, and career choices. *The Q. J. Econ.* **129**, 1409–1447 (2014).
8. Buser, T., Geijtenbeek, L. & Plug, E. Sexual orientation, competitiveness and income. *J. Econ. Behav. & Organ.* **151**, 191–198 (2018).
9. Kamas, L. & Preston, A. Can social preferences explain gender differences in economic behavior? *J. Econ. Behav. & Organ.* **116**, 525–539 (2015).
10. Reuben, E., Sapienza, P. & Zingales, L. Taste for competition and the gender gap among young business professionals. Working Paper 21695, National Bureau of Economic Research (2015).
11. Berge, L. I. O., Bjorvatn, K., Pires, A. J. G. & Tungodden, B. Competitive in the lab, successful in the field? *J. Econ. Behav. & Organ.* **118**, 303–317 (2015).
12. Balafoutas, L. & Sutter, M. How uncertainty and ambiguity in tournaments affect gender differences in competitive behavior. *Eur. Econ. Rev.* **118**, 1–13 (2019).
13. Saccardo, S., Pietrasz, A. & Gneezy, U. On the size of the gender difference in competitiveness. *Manag. Sci.* **64**, 1541–1554 (2018).
14. Niederle, M. A gender agenda: A progress report on competitiveness. *Am. Econ. Rev.* **107**, 115–119 (2017).
15. Almås, I., Cappelen, A. W., Salvanes, K. G., Sørensen, E. Ø. & Tungodden, B. Willingness to compete: Family matters. *Manag. Sci.* **62**, 2149–2162 (2016).
16. Sutter, M. & Glätzle-Rützler, D. Gender differences in the willingness to compete emerge early in life and persist. *Manag. Sci.* **61**, 2339–2354 (2015).
17. Datta Gupta, N., Poulsen, A. & Villeval, M. C. Gender matching and competitiveness: Experimental evidence. *Econ. Inq.* **51**, 816–835 (2013).

18. Niederle, M. & Vesterlund, L. Do women shy away from competition? Do men compete too much? *The Q. J. Econ.* **122**, 1067–1101 (2007).
19. Gneezy, U., Leonard, K. L. & List, J. A. Gender differences in competition: Evidence from a matrilineal and a patriarchal society. *Econometrica* **77**, 1637–1664 (2009).
20. Hogarth, R. M., Karelaia, N. & Trujillo, C. A. When should i quit? gender differences in exiting competitions. *J. Econ. Behav. & Organ.* **83**, 136–150 (2012).
21. Andersen, S., Ertac, S., Gneezy, U., List, J. A. & Maximiano, S. Gender, competitiveness, and socialization at a young age: Evidence from a matrilineal and a patriarchal society. *Rev. Econ. Stat.* **95**, 1438–1443 (2013).
22. Booth, A. & Nolen, P. Choosing to compete: How different are girls and boys? *J. Econ. Behav. & Organ.* **81**, 542–555 (2012).
23. Cárdenas, J. C., Dreber, A., von Essen, E. & Ranehill, E. Cooperativeness and competitiveness in children. *J. Behav. Exp. Econ.* **59**, 32–41 (2015).
24. Baldiga, N. R. & Coffman, K. B. Laboratory evidence on the effects of sponsorship on the competitive preferences of men and women. *Manag. Sci.* **64**, 888–901 (2018).
25. Leibbrandt, A., Wang, L. C. & Foo, C. Gender quotas, competitions, and peer review: Experimental evidence on the backlash against women. *Manag. Sci.* **64**, 3501–3516 (2018).
26. Balafoutas, L. & Sutter, M. Affirmative action policies promote women and do not harm efficiency in the laboratory. *Science* **335**, 579–582 (2012).
27. Balafoutas, L., Fornwagner, H. & Sutter, M. Closing the gender gap in competitiveness through priming. *Nat. Commun.* **9**, 1–6 (2018).
28. Cadsby, C. B., Servátka, M. & Song, F. How competitive are female professionals? A tale of identity conflict. *J. Econ. Behav. & Organ.* **92**, 284–303 (2013).
29. Wozniak, D., Harbaugh, W. T. & Mayr, U. The effect of feedback on gender differences in competitive choices. Working Paper 1976073, SSRN (2016). DOI: http://dx.doi.org/10.2139/ssrn.1976073.
30. Kessel, D., Mollerstrom, J. & van Veldhuizen, R. Can simple advice eliminate the gender gap in willingness to compete? *Eur. Econ. Rev.* **138**, 103777, DOI: https://doi.org/10.1016/j.euroecorev.2021.103777 (2021).
31. Brandts, J., Groenert, V. & Rott, C. The impact of advice on women's and men's selection into competition. *Manag. Sci.* **61**, 1018–1035 (2015).
32. Cassar, A., Wordofa, F. & Zhang, Y. J. Competing for the benefit of offspring eliminates the gender gap in competitiveness. *Proc. Natl. Acad. Sci.* **113**, 5201–5205 (2016).
33. Ranehill, E. *et al.* Hormonal contraceptives do not impact economic preferences: Evidence from a randomized trial. *Manag. Sci.* **64**, 4515–4532 (2018).
34. Dreber, A. & Johannesson, M. *Sex hormones and economic decision making in the lab: A review of the causal evidence* (Routledge, 2018).
35. Wozniak, D., Harbaugh, W. T. & Mayr, U. The menstrual cycle and performance feedback alter gender differences in competitive choices. *J. Labor Econ.* **32**, 161–198 (2014).
36. Barsky, R. B., Juster, F. T., Kimball, M. S. & Shapiro, M. D. Preference parameters and behavioral heterogeneity: An experimental approach in the health and retirement study. *The Q. J. Econ.* **112**, 537–579 (1997).
37. Anderson, L. R. & Mellor, J. M. Predicting health behaviors with an experimental measure of risk preference. *J. Heal. Econ.* **27**, 1260–1274 (2008).
38. Almenberg, J. & Dreber, A. Gender, stock market participation and financial literacy. *Econ. Lett.* **137**, 140–142 (2015).
39. Sutter, M., Kocher, M. G., Glätzle-Rützler, D. & Trautmann, S. T. Impatience and uncertainty: Experimental decisions predict adolescents' field behavior. *Am. Econ. Rev.* **103**, 510–31 (2013).
40. Bonin, H., Dohmen, T., Falk, A., Huffman, D. & Sunde, U. Cross-sectional earnings risk and occupational sorting: The role of risk attitudes. *Labour Econ.* **14**, 926–937 (2007).
41. Guiso, L. & Paiella, M. Risk aversion, wealth, and background risk. *J. Eur. Econ. Assoc.* **6**, 1109–1150 (2008).
42. Bucciol, A. & Miniaci, R. Household portfolios and implicit risk preference. *Rev. Econ. Stat.* **93**, 1235–1250 (2011).
43. Von Gaudecker, H.-M., Van Soest, A. & Wengstrom, E. Heterogeneity in risky choice behavior in a broad population. *Am. Econ. Rev.* **101**, 664–94 (2011).
44. Gollier, C. *The economics of risk and time* (MIT press, 2001).
45. Charness, G. & Gneezy, U. Strong evidence for gender differences in risk taking. *J. Econ. Behav. & Organ.* **83**, 50–58 (2012).
46. Holt, C. A. & Laury, S. K. Risk aversion and incentive effects. *Am. Econ. Rev.* **92**, 1644–1655 (2002).

47. Gneezy, U. & Potters, J. An experiment on risk taking and evaluation periods. *The Q. J. Econ.* **112**, 631–645 (1997).
48. Czibor, E., Claussen, J. & Van Praag, M. Women in a men's world: Risk taking in an online card game community. *J. Econ. Behav. & Organ.* **158**, 62–89 (2019).
49. Croson, R. & Gneezy, U. Gender differences in preferences. *J. Econ. Lit.* **47**, 448–474 (2009).
50. Hardies, K., Breesch, D. & Branson, J. Gender differences in overconfidence and risk taking: Do self-selection and socialization matter? *Econ. Lett.* **118**, 442–444 (2013).
51. Nelson, J. A. Not-so-strong evidence for gender differences in risk taking. *Fem. Econ.* **22**, 114–142 (2016).
52. Nelson, J. A. Are women really more risk-averse than men? a re-analysis of the literature using expanded methods. *J. economic surveys* **29**, 566–585 (2015).
53. Hyde, J. S. The gender similarities hypothesis. *Am. psychologist* **60**, 581 (2005).
54. Nelson, J. A. The power of stereotyping and confirmation bias to overwhelm accurate assessment: the case of economics, gender, and risk aversion. *J. Econ. Methodol.* **21**, 211–231 (2014).
55. Atkinson, S. M., Baird, S. B. & Frye, M. B. Do female mutual fund managers manage differently? *J. Financial Res.* **26**, 1–18 (2003).
56. Adams, R. B. & Funk, P. Beyond the glass ceiling: Does gender matter? *Manag. Sci.* **58**, 219–235 (2012).
57. Drupp, M. A., Khadjavi, M., Riekhof, M.-C. & Voss, R. Professional identity and the gender gap in risk-taking. evidence from field experiments with scientists. *J. Econ. Behav. & Organ.* **170**, 418–432 (2020).
58. Zethraeus, N. *et al.* A randomized trial of the effect of estrogen and testosterone on economic behavior. *Proc. Natl. Acad. Sci.* **106**, 6535–6538 (2009).
59. Boksem, M. A. *et al.* Testosterone inhibits trust but promotes reciprocity. *Psychol. Sci.* **24**, 2306–2314 (2013).
60. Buskens, V., Raub, W., Van Miltenburg, N., Montoya, E. R. & Van Honk, J. Testosterone administration moderates effect of social environment on trust in women depending on second-to-fourth digit ratio. *Sci. Reports* **6**, 1–8 (2016).
61. Anderson, A., Dreber, A. & Vestman, R. Risk taking, behavioral biases and genes: Results from 149 active investors. *J. Behav. Exp. Finance* **6**, 93–100 (2015).
62. Cesarini, D., Dawes, C. T., Johannesson, M., Lichtenstein, P. & Wallace, B. Genetic variation in preferences for giving and risk taking. *The Q. J. Econ.* **124**, 809–842 (2009).
63. Cesarini, D., Johannesson, M., Lichtenstein, P., Sandewall, Ö. & Wallace, B. Genetic variation in financial decision-making. *The J. Finance* **65**, 1725–1754 (2010).
64. Cesarini, D., Johannesson, M., Magnusson, P. K. & Wallace, B. The behavioral genetics of behavioral anomalies. *Manag. Sci.* **58**, 21–34 (2012).
65. Keaveney, A., Peters, E. & Way, B. Effects of acetaminophen on risk taking. *Soc. Cogn. Affect. Neurosci.* **15**, 725–732 (2020).
66. Erb, H.-P., Bioy, A. & Hilton, D. J. Choice preferences without inferences: Subconscious priming of risk attitudes. *J. Behav. Decis. Mak.* **15**, 251–262 (2002).
67. Gilad, D. & Kliger, D. Priming the risk attitudes of professionals in financial decision making. *Rev. Finance* **12**, 567–586 (2008).
68. Guiso, L., Sapienza, P. & Zingales, L. Time varying risk aversion. *J. Financial Econ.* **128**, 403–421 (2018).
69. König-Kersting, C. & Trautmann, S. T. Countercyclical risk aversion: Beyond financial professionals. *J. Behav. Exp. Finance* **18**, 94–101 (2018).
70. Newell, B. R. & Shaw, B. Priming risky choice: Do risk preferences need inferences? *J. Behav. Decis. Mak.* **30**, 332–346 (2017).
71. Benjamin, D. J., Choi, J. J. & Strickland, A. J. Social identity and preferences. *Am. Econ. Rev.* **100**, 1913–28 (2010).
72. Meier-Pesti, K. & Penz, E. Sex or gender? expanding the sex-based view by introducing masculinity and femininity as predictors of financial risk taking. *J. Econ. Psychol.* **29**, 180–196 (2008).
73. Cohn, A., Fehr, E. & Maréchal, M. A. Do professional norms in the banking industry favor risk-taking? *The Rev. Financial Stud.* **30**, 3801–3823 (2017).
74. Cohn, A., Engelmann, J., Fehr, E. & Maréchal, M. A. Evidence for countercyclical risk aversion: An experiment with financial professionals. *Am. Econ. Rev.* **105**, 860–85 (2015).
75. Alempaki, D., Starmer, C. & Tufano, F. On the priming of risk preferences: The role of fear and general affect. *J. Econ. Psychol.* **75**, 102137 (2019).
76. Callen, M., Isaqzadeh, M., Long, J. D. & Sprenger, C. Violence and risk preference: Experimental evidence from afghanistan. *Am. Econ. Rev.* **104**, 123–48 (2014).

77. Bilén, D., Dreber, A. & Johannesson, M. Are women more generous than men? A meta-analysis. *J. Econ. Sci. Assoc.* **7**, 1–18 (2021).
78. Kahneman, D., Knetsch, J. L. & Thaler, R. H. Fairness and the assumptions of economics. *J. Bus.* **59**, S285–S300 (1986).
79. Forsythe, R., Horowitz, J. L., Savin, N. E. & Sefton, M. Fairness in simple bargaining experiments. *Games Econ. Behav.* **6**, 347–369 (1994).
80. Eckel, C. C. & Grossman, P. J. Altruism in anonymous dictator games. *Games Econ. Behav.* **16**, 181–191 (1996).
81. Carpenter, J., Connolly, C. & Myers, C. K. Altruistic behavior in a representative dictator experiment. *Exp. Econ.* **11**, 282–298 (2008).
82. Benz, M. & Meier, S. Do people behave in experiments as in the field?—evidence from donations. *Exp. Econ.* **11**, 268–281 (2008).
83. Franzen, A. & Pointner, S. The external validity of giving in the dictator game. *Exp. Econ.* **16**, 155–169 (2013).
84. Galizzi, M. M. & Navarro-Martinez, D. On the external validity of social preference games: a systematic lab-field study. *Manag. Sci.* **65**, 976–1002 (2019).
85. Zak, P. J. *et al.* Testosterone administration decreases generosity in the ultimatum game. *PLOS ONE* **4**, e8330 (2009).
86. Reuter, M., Frenzel, C., Walter, N. T., Markett, S. & Montag, C. Investigating the genetic basis of altruism: the role of the comt val158met polymorphism. *Soc. Cogn. Affect. Neurosci.* **6**, 662–668 (2011).
87. Benjamin, D. J., Choi, J. J. & Fisher, G. Religious identity and economic behavior. *Rev. Econ. Stat.* **98**, 617–637 (2016).
88. Shariff, A. F. & Norenzayan, A. God is watching you: Priming god concepts increases prosocial behavior in an anonymous economic game. *Psychol. Sci.* **18**, 803–809 (2007).
89. McKay, R., Efferson, C., Whitehouse, H. & Fehr, E. Wrath of god: Religious primes and punishment. *Proc. Royal Soc. B: Biol. Sci.* **278**, 1858–1863 (2011).
90. Ahmed, A. M. & Salas, O. Implicit influences of christian religious representations on dictator and prisoner's dilemma game decisions. *The J. Socio-Economics* **40**, 242–246 (2011).
91. Rigdon, M., Ishii, K., Watabe, M. & Kitayama, S. Minimal social cues in the dictator game. *J. Econ. Psychol.* **30**, 358–367 (2009).
92. Haley, K. J. & Fessler, D. M. Nobody's watching?: Subtle cues affect generosity in an anonymous economic game. *Evol. Hum. Behav.* **26**, 245–256 (2005).
93. Bateson, M., Nettle, D. & Roberts, G. Cues of being watched enhance cooperation in a real-world setting. *Biol. Lett.* **2**, 412–414 (2006).
94. Boschini, A., Dreber, A., von Essen, E., Muren, A. & Ranehill, E. Gender and altruism in a random sample. *J. Behav. Exp. Econ.* **77**, 72–77, DOI: https://doi.org/10.1016/j.socec.2018.09.005 (2018).

15 Additional information

15.1 Study sample

We recruited a total of 798 participants. Please note that due to a technical problem on how the participant's performance was shown to them on their screen, we exclude $n = 3$ cisgender and $n = 6$ transgender observations. We tested with our debriefing questionnaire whether the participants had an idea about the aim of the study, the study topic, etc. Eight cisgender and one transgender participant(s) wrote to think that s/he were primed. These $n = 9$ observations are also excluded from our analysis. Thus, the final number of subjects by subject groups and treatment is 780 as summarized in Table 49.

To have comparable transgender and cisgender observations, we first collected the major part of the transgender observations, including their main demographic characteristics (age, student status, education, income, religious affiliation, and residence) of the transgender participants. We then used Prolific's sorting tool to recruit a similar cisgender sample based on those criteria.

Supplementary Table 49 Distribution of subject groups across treatments

	Treatment			
	NEUTRAL	FEMININE	MASCULINE	Total
Cismen	72	71	71	214
Ciswomen	71	70	70	211
Transmen	72	72	71	215
Transwomen	44	50	46	140
Total	259	263	258	780

15.2 Datasets

gender_data.csv S1 is the main data set. The file contains n=798 observations and 103 variables. Details on the variables can be found in the second dataset S2 (codebook.csv).

codebook.csv This S2 file provides the details on the variables of the main data set S1. Each row includes the explanations for one of the 103 variables. Additionally, the third column summarizes the response options the subjects had.

The collected data and additional material is available at OSF using `https://osf.io/tyzjh/?view_only=66a8abca5f6a4aeead68f6fef19a0ee9`

15.3 Instructions

The following pages contain screenshots of the online study conducted on the platform Prolific. Please note that one participant was randomly allocated to just one treatment. Thus, one participant saw one out of the three different treatment pages. In addition, depending on the choice made in Part 4, the system showed one of two options pages. A blue headline marks the varying screens. All other pages were identical.

Welcome!

Please read the following.

Dear participant,
The following will provide you with information about the experiment that will help you decide whether you wish to participate. The study received two certificates of good standing (ethical approvals). If you agree to participate, please be aware that you are free to withdraw at any point throughout the study. All data collected for this scientific study will remain confidential and anonymous. If you have any further questions concerning this study, please feel free to contact us via phone or email:

Dr. Silvio Städter at silvio.staedter@ur.de or +49 941 9433259, or the team at econ.study.research@gmail.com.

Please indicate that by clicking the following box on the space below, you understand your rights and agree to participate in the experiment. You can revoke the consent to the collection and processing of the data at any time by just closing the internet browser via which you participate. After your revocation, no further data will be collected. However, the data collected up to the point of cancellation can continue to be used in this study. Your participation is solicited yet strictly voluntary. All information will be kept confidential, and your name will not be associated with any research findings.

☐ *I understand my rights and agree to participate in the experiment.*

Continue

Welcome!

Thank you very much for participating in our study!

This is a project of researchers from the University of Exeter (United Kingdom), the Chulalongkorn University (Thailand), and the University of Regensburg (Germany). The study is conducted by Prof. Brit Grosskopf, Chanalak Chaisrilak, Dr. Helena Fornwagner, Alexander Lauf, Vanessa Schöller, and Dr. Silvio Städter.

The study received two certificates of good standing (ethical approvals).

The study consists of **six parts** and a **questionnaire**.

Depending on the decisions you take in the different parts, you can earn money.

Please note that all decisions you take, as well as all data that is collected with the survey is anonymous and only used for this study.

Before each part starts, you will get detailed instructions, what you have to do and how the decisions you take influence your payment.

In each part, you can earn money. At the end of the study, you can see how much you earned in each part. We will then randomly select one out of the six parts and pay you the money you have earned in this part.

For completing the study you will receive a compensation of **£3.75** for sure.

So your total payment will be your earnings in one randomly chosen part plus the compensation for completing the study. At the end of the study, you will receive your personal unique **8-digit Code** as proof that you have completed the study.

If you need any further details or have problems when conducting the study, please contact Dr. Silvio Städter at silvio.staedter@ur.de or +49 941 9433259, or the team at econ.study.research@gmail.com.

Please enter your personal Code in Prolific. Then, you get **£3.75** and the additional money you earned in the randomly selected part via Prolific.

Thank you again for taking your time to participate,
Prof. Brit Grosskopf,
Chanalak Chaisrilaky, MSc,
Dr. Helena Fornwagner,
Alexander Lauf, MSc,
Vanessa Schöller, MSc, and
Dr. Silvio Städter.

Start the Study

Part 1: Instructions

In this game you have to solve a **Word Search Puzzle**.

On the next page, you will see a table with **10** rows and **10** columns, a word puzzle. Make sure you can see the entire table.

Within this table **8 words** are hidden. They are either **vertically**, **horizontally** or **diagonally** placed in the table.

These 8 words are revealed on the next page, just above the table. Your task is to find these 8 hidden words and mark them. To mark a word, you have to keep the left mouse button down and go over the word's letters.

Marked words are highlighted in blue. If you mark a word, it will be crossed out in the list of words. You have to mark one word in one go.

If this part is randomly selected for payment at the end of the study and you have found **all 8 words**, you get **£5.00**.
If you didn't find all 8 words, you get nothing in this part.

You cannot go back to this page and please do not reload the page.

You have **120 seconds** to find the 8 words.

If you are ready, click on the button 'Start this part' below.

Start this part

Treatment: NEUTRAL

Part 1: Word Search

Time left to complete this page: **1:56**

Please find the following words in the word puzzle.

neuter	its
person	child
it	theirs
people	individual

E	G	J	U	R	D	I	T	S	A
S	N	E	U	T	E	R	S	B	O
A	C	P	E	O	P	L	E	I	T
U	H	S	V	V	J	R	B	U	Y
N	I	K	O	W	R	B	Y	U	W
C	L	C	D	L	F	O	M	K	M
V	D	K	F	L	U	L	D	Y	E
J	L	R	C	T	H	E	I	R	S
J	G	P	E	R	S	O	N	E	L
I	N	D	I	V	I	D	U	A	L

Continue

Treatment: FEMININE

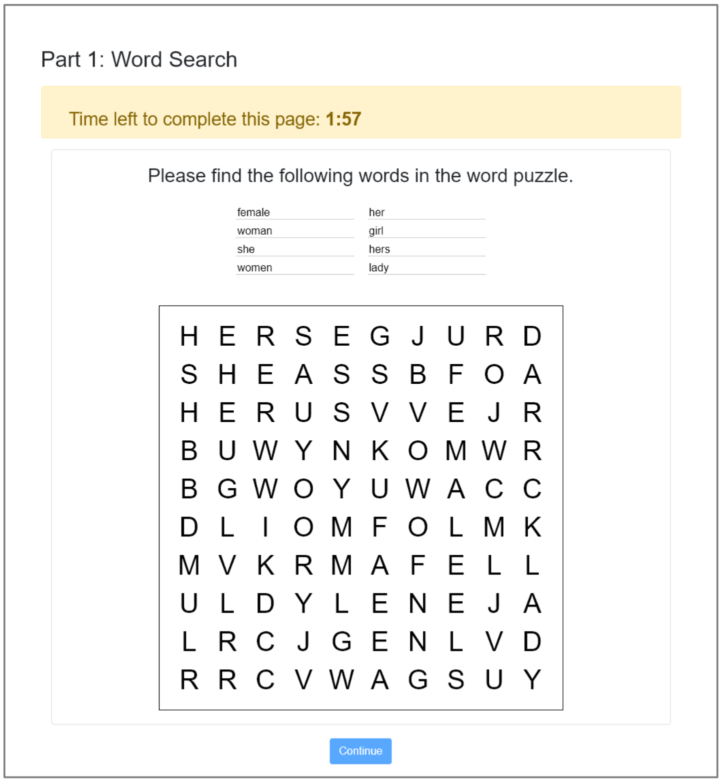
Part 1: Word Search

Time left to complete this page: **1:57**

Please find the following words in the word puzzle.

female	her
woman	girl
she	hers
women	lady

H	E	R	S	E	G	J	U	R	D
S	H	E	A	S	S	B	F	O	A
H	E	R	U	S	V	V	E	J	R
B	U	W	Y	N	K	O	M	W	R
B	G	W	O	Y	U	W	A	C	C
D	L	I	O	M	F	O	L	M	K
M	V	K	R	M	A	F	E	L	L
U	L	D	Y	L	E	N	E	J	A
L	R	C	J	G	E	N	L	V	D
R	R	C	V	W	A	G	S	U	Y

Continue

Treatment: MASCULINE

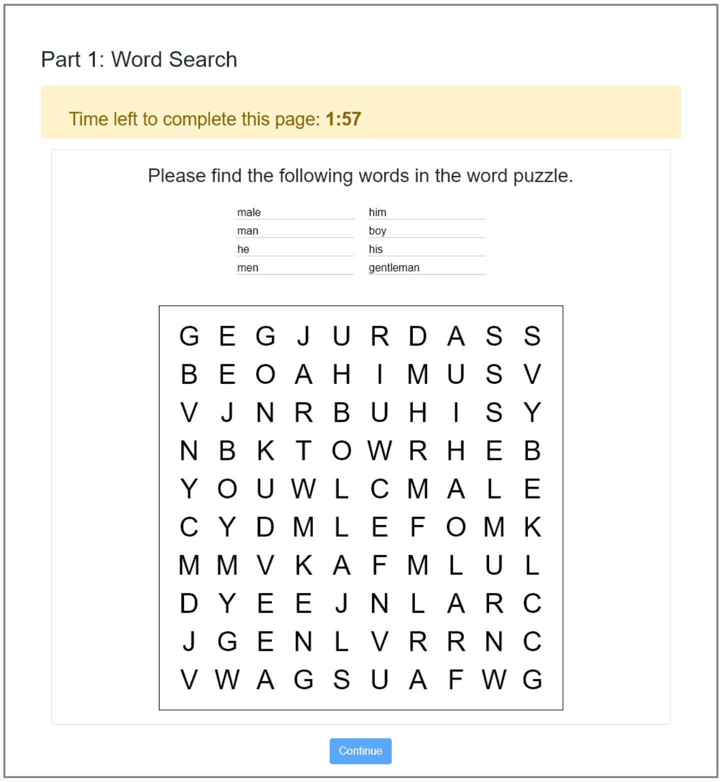
Part 1: Word Search

Time left to complete this page: **1:57**

Please find the following words in the word puzzle.

male	him
man	boy
he	his
men	gentleman

G	E	G	J	U	R	D	A	S	S
B	E	O	A	H	I	M	U	S	V
V	J	N	R	B	U	H	I	S	Y
N	B	K	T	O	W	R	H	E	B
Y	O	U	W	L	C	M	A	L	E
C	Y	D	M	L	E	F	O	M	K
M	M	V	K	A	F	M	L	U	L
D	Y	E	E	J	N	L	A	R	C
J	G	E	N	L	V	R	R	N	C
V	W	A	G	S	U	A	F	W	G

Continue

Part 1: Summary

You have finished this part.

You found **8 of 8** words.

Continue to next part

Part 2, Part 3, and Part 4

Please complete Part 2, Part 3 and Part 4 now.

In each part, you have to perform the **same task**. In this task, you have to solve as many short math quizzes as you can within 120 seconds in each part. The number of correctly solved quizzes determines your payment in each part.

Before you can start to earn money in each part, you get detailed instructions on how your payment is determined in the following part.

After the instructions for the first part, you get to practice the task for 90 seconds. The quizzes solved for practice purposes are not payoff relevant.

Continue

Part 2: Instructions

Instructions: Math Quizzes

In this task, you have to solve as many quizzes as possible within 120 seconds. For each correctly solved quiz you can earn money as explained under '*Payment*' after the practice round.

Each quiz consists of 9 two-digit numbers. You can see an example of a quiz below.

Quiz 1

You solved 0 of 0 correctly.

Please select 2 numbers that add up to 100.

10	15	56
87	75	25
20	31	14

Submit

In each quiz, you have to select two numbers that add up to 100 by clicking on them. If you have selected two numbers, you can submit your selection by clicking on the button 'Submit'.

You cannot select more than two numbers. So if you want to change your selection, unselect one of your choices by clicking a second time on your choice and select the one you want.

After submitting an answer, you will get a new quiz. Below the quiz number, you can see how many quizzes you have already solved correctly.

You have a time limit of **120 seconds** to complete as many quizzes as possible in each part.

You can continue to the practice round, where you can practice the exercise.

Continue to Practice Round

Part 2: Practice Round

Here, you have **90 seconds** to get to know the task and practice.

The math quizzes solved in the practice round have no impact on your payment.

If you have selected two numbers, you can click the "Submit" button.
The computer will tell you, whether you were right or not.

Please, solve at least 1 quiz without reloading this page.

Practice Quiz 1

You solved 0 of 0 correctly.

Please select 2 numbers that add up to 100.

14	10	34
84	98	95
66	21	24

Submit

If you are ready, click on the button 'Start the Practice' below.

Start the Practice

Part 2: Practice Round

Time left to practice: **1:21**

Here, you have **90 seconds** to get to know the task and practice.

The math quizzes solved in the practice round have no impact on your payment.

If you have selected two numbers, you can click the "Submit" button.
The computer will tell you, whether you were right or not.

Please, solve at least 1 quiz without reloading this page.

Practice Quiz 2

You solved 1 of 1 correctly.

Please select 2 numbers that add up to 100.

20	13	55
80	92	12
22	54	40

Submit

If you solved 1 quiz, you can continue.

Continue

Part 2: Payment Details

Payment PIECE RATE

- The money you can earn in this part depends only on your own performance.
- You receive **1 point** for each correctly solved quiz.
- You do not loose points if you submit a wrong answer.
- If this part is randomly selected for payment at the end of the study, you get **£0.50** for each collected point.

If you are ready, click on the button 'Start this part' below.
You have 120 seconds and your submissions are relevant for your payment.

Start this part

Part 2: Math Quizzes

Time left to solve quizzes: **1:58**

Quiz 1

You solved 0 of 0 correctly.

Please select 2 numbers that add up to 100.

38	34	15
96	58	31
85	36	63

Submit

Part 2: Bonus Question

Please answer the following bonus question

You now have the chance to earn additional money. If this part is randomly chosen for payment, you will be paid an extra £1.00 on top of your final payoff if your following answer is correct.

Suppose we group all participants of this study into 4 groups by their achieved points and assume we have 100 participants for simplicity. First, we put the 25 participants with the highest amounts of points in Group 1. The next 25 participants with the second highest amounts of points in Group 2 and the following 25 in Group 3. The 25 participants with the fewest amounts of points are in Group 4.

With the points you collected, to which group do you think you belong?

- ○ I would be in Group 1.
- ○ I would be in Group 2.
- ○ I would be in Group 3.
- ○ I would be in Group 4.

Continue to Summary

Part 2: Summary

Result

You solved **1** of **1** quizzes correctly.

Continue to next part

Part 3: Payment Details

Payment TOURNAMENT

- For this part, you are now randomly matched with **three other participants** of this study. You are **one group**. You will not get to know who the other participants are and the other participants will not get to know who you are.
- The money you can earn in this part depends on your performance and the performance of the other group members. Thus, you are in a **tournament** with the other group members.
- You get again **1 point** for each correctly solved quiz and you do not loose points if you submit a wrong answer.
- If this part is randomly selected for payment at the end of the study, your payment is determined as follows:
    - If you collect *more points* than all other members of your group, you are the winner of this tournament and get **£2.00** for each point.
    - If you collect *less points* than the best player in your group, you get **nothing** in this part.
    - If there are ties, the winner will be randomly determined.

If you are ready, click on the button 'Start this part' below.
You have 120 seconds and your submissions are relevant for your payment.

Start this part

Part 3: Math Quizzes

Time left to solve quizzes: **1:40**

Quiz 3

You solved 2 of 2 correctly.

Please select 2 numbers that add up to 100.

14	20	72
24	39	81
98	82	19

Submit

Part 3: Bonus Question

Please answer the following bonus question

You now have the chance to earn additional money. If this part is randomly chosen for payment, you will be paid an extra £1.00 on top of your final payoff if your following answer is correct.

How well did you perform in this part relative to your group members?

- ○ I was the best.
- ○ I was the second best.
- ○ I was the third best.
- ○ I was last.

Continue to Summary

Part 3: Summary

Result

You solved **2** of **2** quizzes correctly.

Continue to next part

Part 4: Your Choice

Please choose one of the two options

In this part, you can choose which payment scheme from the previous two parts should apply to your performance.

You can choose **Option A: PIECE RATE** from Part 2 or **Option B: TOURNAMENT** from Part 3.

Below you can see both options, and the respective resulting payment rules.

Option A: PIECE RATE

Payment

- The money you can earn in this part depends only on your own performance.
- You receive **1 point** for each correctly solved quiz.
- You do not loose points if you submit a wrong answer.
- If this part is randomly selected for payment at the end of the study, you get **£0.50** for each collected point.

Option B: TOURNAMENT

If you choose Option B, we compare your points to the points of your group members from the previous part. Hence, we take your points from this part and compare them with the other group members' points in Part 3. This means that your choice in this part has no impact on the payment of the other group members.

Payment

- You get again **1 point** for each correctly solved quiz and you do not loose points if you submit a wrong answer.
- If this part is randomly selected for payment at the end of the study, your payment is determined as follows:
    - If you collect *more points* than all other members of your group in Part 3, you are the winner of this tournament and get **£2.00** for each point.
    - If you collect *less points* than the best player in your group in Part 3, you get **nothing** in this part.
    - If there are ties, the winner will be randomly determined.

Please choose:

Option A or Option B

Choice: Option A

Part 4: Payment Details

You have chosen **Option A**.

Payment

- The money you can earn in this part depends only on your own performance.
- You receive **1 point** for each correctly solved quiz.
- You do not loose points if you submit a wrong answer.
- If this part is randomly selected for payment at the end of the study, you get **£0.50** for each collected point.

If you are ready, click on the button 'Start this part' below.
You have 120 seconds and your submissions are relevant for your payment.

Start this part

Choice: Option B

Part 4: Payment Details

You have chosen **Option B**.

Payment

- You get again **1 point** for each correctly solved quiz and you do not loose points if you submit a wrong answer.
- If this part is randomly selected for payment at the end of the study, your payment is determined as follows:
  - If you collect *more points* than all other members of your group in Part 3, you are the winner of this tournament and get **£2.00** for each point.
  - If you collect *less points* than the best player in your group in Part 3, you get **nothing** in this part.
  - If there are ties, the winner will be randomly determined.

If you are ready, click on the button 'Start this part' below.
You have 120 seconds and your submissions are relevant for your payment.

Start this part

Part 4: Math Quizzes

Time left to solve quizzes: **1:18**

Quiz 4

You solved 3 of 3 correctly.

Please select 2 numbers that add up to 100.

15	26	31
18	85	79
55	30	57

Submit

Part 4: Summary

Result

You solved **3** of **3** quizzes correctly.

Continue to next part

Part 5: Instructions

In this part, you get a start capital of **£4.00** and you have to decide how much of it you want to invest into a risky lottery.

The success of this investment is decided by *flipping a virtual coin*. You can get 'heads' or 'tails' with an equal probability. That means you have a 50% chance of success.

If you get **'tails'**, your investment was successful. In this case, the amount you have invested is multiplied by **2.5**. Your earnings are then the leftover of your start capital and the money from the successful investment.

But if you get **'heads'**, your investment was not successful, and you have only the leftover of your start capital.

There are no restrictions: you can put everything of your start capital in the investment, or nothing, or anything in between.

On the next page, you can decide how much to invest and see the potential outcomes of your investment.

Continue

Part 5: Your Decision

Your Decision

How much of your £4.00 do you want to invest?

4 £

If you have set the amount you want to invest, click on the button "Invest" below.

Under "Potential Outcome" to the right, you can see what can happen when you click "Invest".

Potential Outcome

You want to invest: £4.00

You want to keep: £0.00

If the coin shows 'tails', you additionally get £10.00.
So in total you get: £0.00 + £10.00 = **£10.00**

If the coin shows 'heads', you get £0.
So in total you get: £0.00 + £0 = **£0.00**

If you have made your decision, please click on the button 'Invest' below.

Invest

Part 5: Summary

You started with a capital of **£4.00**.

You invested **£4.00**.

We are flipping the virtual coin to see if your investment is a success or not. You will see the result at the end of the experiment.

Continue to next Part

Part 6: Instructions

You will receive an initial endowment of **£5.00** for this part.

On the next page, you can decide how much of this endowment you want to keep for yourself and how much you would like to donate to five different charities.

You can freely set the share you want to keep and the share you want to transfer to one or more respective charities. Any amount between 0 and £5.00 is possible, as long as the sum of all options equals your endowment.

Once we have finished the study, we will send the respective donations of all participants to the charities.

Continue

Part 6: Your Donation

Please indicate the amount you want to donate.

You have received an initial endowment of £5.00.

Remember that the sum must be equal to your endowment.

Amnesty International:

1 £

Doctors without Borders:

1 £

International Red Cross:

1 £

UNICEF:

1 £

WWF:

1 £

Your share:

0.00 £

Submit

Part 6: Summary

You started with an endowment of **£5.00**.

You kept for yourself **£0.00**.

You donated **£5.00**.

Continue to next Part

Questionnaire

You have finished the six parts.

Now, we kindly ask you to answer the following questions.

At the end, you will get your personal unique **8-digit Code** for the completion of the study.

Start Questionnaire

Questionnaire

Below you find several personality traits that may or may not apply to you.

Please indicate for each trait the extent to which it applies to you on a scale from 1 (*Never true*) to 6 (*Always true*).

**Trait**	*Never true*	*Sometimes true*	*Occasionally true*	*Often true*	*Usually true*	*Always true*
Defends own beliefs	○ 1	○ 2	○ 3	○ 4	○ 5	○ 6
Tender	○ 1	○ 2	○ 3	○ 4	○ 5	○ 6
Conscientious	○ 1	○ 2	○ 3	○ 4	○ 5	○ 6
Independent	○ 1	○ 2	○ 3	○ 4	○ 5	○ 6
Sympathetic	○ 1	○ 2	○ 3	○ 4	○ 5	○ 6
Moody	○ 1	○ 2	○ 3	○ 4	○ 5	○ 6
Assertive	○ 1	○ 2	○ 3	○ 4	○ 5	○ 6
Sensitive to needs of other	○ 1	○ 2	○ 3	○ 4	○ 5	○ 6
Reliable	○ 1	○ 2	○ 3	○ 4	○ 5	○ 6
Strong personality	○ 1	○ 2	○ 3	○ 4	○ 5	○ 6
Understanding	○ 1	○ 2	○ 3	○ 4	○ 5	○ 6
Jealous	○ 1	○ 2	○ 3	○ 4	○ 5	○ 6
Forceful	○ 1	○ 2	○ 3	○ 4	○ 5	○ 6
Compassionate	○ 1	○ 2	○ 3	○ 4	○ 5	○ 6
Truthful	○ 1	○ 2	○ 3	○ 4	○ 5	○ 6

Continue

Questionnaire

Below you find several personality traits that may or may not apply to you.

Please indicate for each trait the extent to which it applies to you on a scale from 1 (*Never true*) to 6 (*Always true*).

**Trait**	*Never true*	*Sometimes true*	*Occasionally true*	*Often true*	*Usually true*	*Always true*
Has leader abilities	○ 1	○ 2	○ 3	○ 4	○ 5	○ 6
Eager to soothe hurt feelings	○ 1	○ 2	○ 3	○ 4	○ 5	○ 6
Secretive	○ 1	○ 2	○ 3	○ 4	○ 5	○ 6
Willing to take risk	○ 1	○ 2	○ 3	○ 4	○ 5	○ 6
Warm	○ 1	○ 2	○ 3	○ 4	○ 5	○ 6
Adaptable	○ 1	○ 2	○ 3	○ 4	○ 5	○ 6
Dominant	○ 1	○ 2	○ 3	○ 4	○ 5	○ 6
Affectionate	○ 1	○ 2	○ 3	○ 4	○ 5	○ 6
Conceited	○ 1	○ 2	○ 3	○ 4	○ 5	○ 6
Willing to take a stand	○ 1	○ 2	○ 3	○ 4	○ 5	○ 6
Loves children	○ 1	○ 2	○ 3	○ 4	○ 5	○ 6
Tactful	○ 1	○ 2	○ 3	○ 4	○ 5	○ 6
Aggressive	○ 1	○ 2	○ 3	○ 4	○ 5	○ 6
Gentle	○ 1	○ 2	○ 3	○ 4	○ 5	○ 6
Conventional	○ 1	○ 2	○ 3	○ 4	○ 5	○ 6

Continue

Questionnaire

Gender identity is defined as the gender that you identify yourself with. It is not necessarily related to your assigned sex at birth.

For the following statements, please indicate the response that best describes your experience over the past two weeks on a scale from 1 (*Strongly disagree*) to 5 (*Strongly agree*).

My outward appearance represents my gender identity.						
Strongly disagree	○ 1	○ 2	○ 3	○ 4	○ 5	*Strongly agree*
I experience a sense of unity between my gender identity and my body.						
Strongly disagree	○ 1	○ 2	○ 3	○ 4	○ 5	*Strongly agree*
My physical appearance adequately expresses my gender identity.						
Strongly disagree	○ 1	○ 2	○ 3	○ 4	○ 5	*Strongly agree*
I am generally comfortable with how others perceive my gender identity when they look at me.						
Strongly disagree	○ 1	○ 2	○ 3	○ 4	○ 5	*Strongly agree*
My physical body represents my gender identity.						
Strongly disagree	○ 1	○ 2	○ 3	○ 4	○ 5	*Strongly agree*
The way my body currently looks does not represent my gender identity.						
Strongly disagree	○ 1	○ 2	○ 3	○ 4	○ 5	*Strongly agree*

Continue

Questionnaire

Gender identity is defined as the gender that you identify yourself with. It is not necessarily related to your assigned sex at birth.

For the following statements, please indicate the response that best describes your experience over the past two weeks on a scale from 1 (*Strongly disagree*) to 5 (*Strongly agree*).

I am happy with the way my appearance expresses my gender identity.

Strongly disagree	○ 1	○ 2	○ 3	○ 4	○ 5	*Strongly agree*

I do not feel that my appearance reflects my gender identity.

Strongly disagree	○ 1	○ 2	○ 3	○ 4	○ 5	*Strongly agree*

I feel that my mind and body are consistent with one another.

Strongly disagree	○ 1	○ 2	○ 3	○ 4	○ 5	*Strongly agree*

I am not proud of my gender identity.

Strongly disagree	○ 1	○ 2	○ 3	○ 4	○ 5	*Strongly agree*

I am happy that I have the gender identity that I do.

Strongly disagree	○ 1	○ 2	○ 3	○ 4	○ 5	*Strongly agree*

I have accepted my gender identity.

Strongly disagree	○ 1	○ 2	○ 3	○ 4	○ 5	*Strongly agree*

Continue

Questionnaire

Please answer the following questions.

Please indicate your age.

What is you body height in cm (for a converter see: https://www.unitconverters.net/length/feet-to-cm.htm)?

Which is the highest level of education you have completed?

What is your personal income per year (after tax) in GBP?

Are you a student?

- Yes
- No

Where are you currently living?

What is your religious affiliation?

What is your biological sex?

What is your gender?

If you selected "Other", please specify:

Who do you feel attracted to: men, women, both or neither?

Are you currently in a relationship and if yes with a man or a woman?

Do you consider yourself transgender?

- Yes
- No

In what kind of accommodation are you currently living?

Apartment House Dormitory Other

Do you currently live with your partner in the same accommodation?

- Yes
- No

Do you currently live in the same city as your parents?

- Yes
- No

Do you currently live with your parents in the same house/apartment?

- Yes
- No

Do you think your parents treated you mostly as a boy or as girl?

Boy Girl They treated me mostly gender neutral.

On which device do you participate in this study?

Tablet Smartphone PC Laptop Other device

Send and Continue

Questionnaire

Please answer the following questions.

What do you think was the purpose of this study? What do you think the study tried to find out?

Did you think any of the previous tasks were related?

○ Yes ○ No

Did anything you do in one task affect what you did in the other tasks?

○ Yes ○ No

Did you ever see or complete a word-search puzzle in another study?

○ Yes ○ No

Do you remember any of the words from the word-search puzzle? If not leave empty.

Did any of the words in the word-search puzzle seem strange or suspicious to you? If not leave empty.

Send and Continue

Questionnaire

Please indicate whether you have taken any of the following actions in order to transition to your gender identity or if it does not apply.

Come out as transgender to family.

○ Yes ○ No ○ Does not apply

Come out as transgender to friends.

○ Yes ○ No ○ Does not apply

Come out as transgender to coworkers or fellow students.

○ Yes ○ No ○ Does not apply

Adopted a name not given at birth that better represents gender identity.

○ Yes ○ No ○ Does not apply

Currently called adopted name by family.

○ Yes ○ No ○ Does not apply

Currently called adopted name by friends.

○ Yes ○ No ○ Does not apply

Currently called adopted name by coworkers or fellow students.

○ Yes ○ No ○ Does not apply

Legally had name change to adopted name.

○ Yes ○ No ○ Does not apply

Wear clothing that matches gender identity in social situations.

○ Yes ○ No ○ Does not apply

Wear clothing that matches gender identity in work/school.

○ Yes ○ No ○ Does not apply

Legally changed sex on birth certificate (if live in state where this is possible).

○ Yes ○ No ○ Does not apply

Driver's license changed to reflect gender identity.

○ Yes ○ No ○ Does not apply

Had surgery to alter genitalia.

○ Yes ○ No ○ Does not apply

Undergoing hormone replacement therapy.

○ Yes ○ No ○ Does not apply

Used or had a nonsurgical cosmetic procedure (e.g., electrolysis) to alter physical appearance in order to make it more congruent with gender identity.

○ Yes ○ No ○ Does not apply

Had non-genital surgery (e.g., breast removal, breast implants, facial femininization surgery, vocal cord surgery) to alter appearance (or presence) in order to make it more congruent with gender identity.

○ Yes ○ No ○ Does not apply

Send and Continue

Selection of your Payment

Your results in each part

Here you can see the outcomes of all six parts.

The computer will randomly select one of these parts on the next screen. Each part has the same probability to be selected.

You will get **£3.75** and the additional money in the column 'payoff' from this part.

	Short summary of the parts		
**Part 1**	You found:	Payment:	Payoff
	8 words	£5.00 if all found	£5.00
**Part 2**	You solved:	Payment:	Payoff
	1 quizzes	£0.50 per quiz	£0.50
**Part 3**	You solved:	Payment:	Payoff
	2 quizzes	£2.00 per quiz<br>only if you are the best	£4.00
**Part 4**	You solved:	Payment:	Payoff
	3 quizzes	*Option A*<br>£0.50 per quiz	£1.50
**Part 5**	You invested:	Success?	Payoff
	£4.00	Yes	£10.00
**Part 6**	You kept:	You donated:	Payoff
	£0.00	£5.00	£0.00

Continue

Thank you very much for participating!

Please write down or take a screenshot of the following information and the 8-digit code below.

The computer selected:

Part 2

Payment	Part payment	**Total payment**
£3.75	£0.50	£4.25

Your personal code:

4588DDFC

Important: Click on the link below to be redirected to Prolific. Clicking is necessary to claim your reward for participating in this study.
https://app.prolific.co/submissions/complete?cc=4588DDFC
